# Regioselective Enzymatic Carboxylation of Bioactive (Poly)phenols

**DOI:** 10.1002/adsc.201601046

**Published:** 2017-01-18

**Authors:** Katharina Plasch, Verena Resch, Julien Hitce, Jarosław Popłoński, Kurt Faber, Silvia M. Glueck

**Affiliations:** ^1^ Department of Chemistry, Organic & Bioorganic ChemistryUniversity of GrazHeinrichstrasse 28A-8010GrazAustria; ^2^ L'Oréal Research & Innovation30 bis rue Maurice Berteaux95500Le ThillayFrance; ^3^ Department of ChemistryWrocław University of Environmental and Life Sciencesul. C. K. Norwida 2550-375WrocławPoland; ^4^ Austrian Centre of Industrial Biotechnology (ACIB)University of GrazHeinrichstrasse 28A-8010GrazAustria

**Keywords:** *ortho*-benzoic acid decarboxylases, bioactive (poly)phenols, biocatalysis, carboxylation, regioselectivity

## Abstract

In order to extend the applicability of the regioselective enzymatic carboxylation of phenols, the substrate scope of *o*‐benzoic acid (de)carboxylases has been investigated towards complex molecules with an emphasis on flavouring agents and polyphenols possessing antioxidant properties. *o*‐Hydroxycarboxylic acid products were obtained with perfect regioselectivity, in moderate to excellent yields. The applicability of this method was proven by the regioselective bio‐carboxylation of resveratrol on a preparative scale with 95% yield.

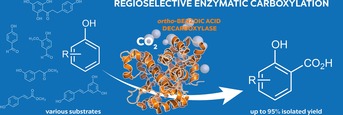

The carboxylation of (hetero)aromatic and phenolic compounds is a convenient method to obtain aromatic carboxylic acids used as pharmaceuticals^1^,^2^ (e.g., salicylic and *m*‐aminosalicylic acid) as well as building blocks for organic synthesis. The traditional chemical (Kolbe–Schmitt) carboxylation process performed on an industrial scale requires high pressure and temperature (∼90 bar, 120–300 °C) and often suffers from incomplete regioselectivities resulting in product mixtures.^3^ In order to circumvent these limitations, various chemical CO_2_ fixation concepts using heterogeneous, (transition) metal and organic catalysts have been established.^4^ A novel attractive biocatalytic alternative is the use of (de)carboxylases, which act at ambient reaction conditions and show perfect regioselectivities.^5^,^6^,^7^

To date, a biocatalytic toolbox for the regioselective *ortho*‐,^1^,^2^,^8^ *para*‐9 and β‐carboxylation^10^ of phenols and hydroxystyrenes, respectively, has been established, which employs decarboxylases acting in the (reverse) carboxylation direction using bicarbonate as CO_2_ source. In addition, electron‐rich heteroaromatics, such as pyrrole and indole were successfully carboxylated.^11^ Enzymatic carboxylation of phenolic substances is a common detoxification pathway in an anaerobic environment^[,12]^ and hence the corresponding enzymes are expected to possess a relaxed substrate tolerance, which is indeed true for the *o*‐carboxylation of phenols^5^,8f and the β‐carboxylation of hydroxystyrenes.^10^ However, the substrates reported so far are predominantly small or medium‐sized phenol derivatives, which were converted at low substrate loading, except for a recently reported study on hydroxystilbenes and the naturally occurring polyphenol resveratrol.8g

Based on our previous studies on the enzymatic *ortho*‐carboxylation of phenols, we aimed to expand the scope of this method towards more complex (poly)phenolic substrates (Scheme 1, Figure 1). The latter compounds are well known for their biological activities such as antioxidant, anti‐inflammatory and antimicrobial properties, which promote them as promising targets for the pharmaceutical industry as well as for the cosmetic and food fields.^13^,^14^,^15^ Enzymatic carboxylation of these compounds would provide an efficient access to more polar derivatives with enhanced water solubility thereby facilitating their formulation and modulating their bioavailability. In addition, the soft electron‐withdrawing effect of the newly introduced carboxylate group should render these products more stable towards autoxidation in analogy to the beneficial effect of the e^−^‐withdrawing carbonyl group of green tea polyphenols.^16^ Furthermore, phenolic acids are expected to impede light‐induced degradation as demonstrated in the case of photolabile substances in food or feed, such as vitamins17a or nucleic acids.17b

**Scheme 1:**
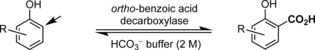
Enzymatic *ortho*‐carboxylation of bioactive (poly)phenols.

**Figure 1:**
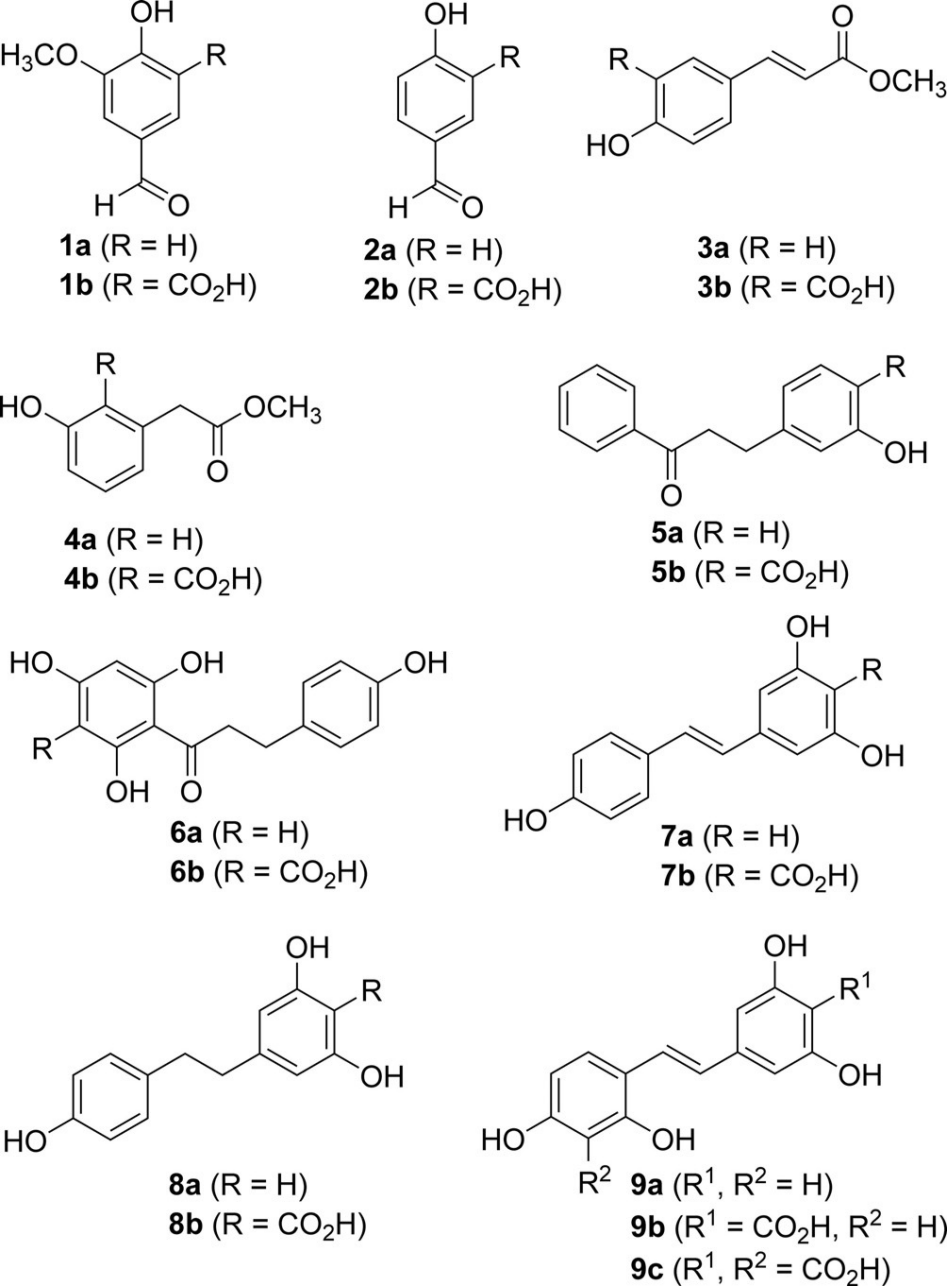
Set of substrates (**1a**–**9a**) and corresponding carboxylated products (**1b**–**9b** and **9c**) obtained from bio‐carboxylation.

The substrate scope of this study ranges from the flavouring agent vanillin (**1a**) to secondary plant metabolites, such as *p*‐hydroxybenzaldehyde (**2a**), esters of *p*‐hydroxycinnamic (*p*‐coumaric) (**3a**) and *m*‐hydroxyphenylacetic acid (**4a**), as well as representatives of flavonoids such as dihydrochalcones (**5a**, **6a**) and hydroxystilbenes, such as resveratrol (**7a**) and resveratrol‐like polyphenols (**8a**, **9a**). Phloretin (**6a**) which is abundantly present in apples is described to possess antioxidant properties and to act as a peroxynitrite scavenger and an inhibitor of lipid peroxidation.13b,^18^ The most famous example among this group of substrates is the natural product resveratrol (**7a**) which has fostered research and development towards cosmetic, food, nutraceutical and pharmaceutical applications, due to its unique ability to modulate physiological as well as pathological pathways.^13^,^19^ In particular, resveratrol (**7a**) exhibits antimicrobial13c and antioxidant13d properties that are expected to be retained in the more soluble carboxylated form **7b**. Furthermore, the γ‐resorcylic acid moiety found in carboxylation products **7b**–**9b** would bring about new biological activities that are not displayed by the corresponding parent compounds **7a**–**9a**. For instance, γ‐resorcylic acid derivatives bearing a lipophilic substituent are known as thrombolytic^20^ and as anti‐inflammatory agents through uncoupling of phosphorylation.^21^ In particular, γ‐resorcylic acids with an alkylaryl substituent in the 4‐position were shown to reduce inflammation *in vivo* in a mice model.^22^

In order to examine the synthetic potential of the method, the most promising substrate candidates were subjected to preparative‐scale carboxylation.

A set of suitable enzyme candidates which have been shown to regioselectively catalyze the *o*‐carboxylation of phenolic compounds was applied: (i) 2,3‐dihydroxybenzoic acid decarboxylase from *Aspergillus oryzae* (2,3‐DHBD_Ao),8f,^23^ (ii) 2,6‐dihydroxybenzoic acid decarboxylases from *Rhizobium* sp. (2,6‐DHBD_Rs)8f,^24^ and (iii) salicylic acid decarboxylase from *Trichosporon moniliiforme* (SAD_Tm).^1^,^2^,8e,8f To simplify handling by avoiding protein purification, the biocatalysts were employed as lyophilized whole‐cell preparations of recombinant overexpressed decarboxylases in *E. coli*. Independent control experiments ensured that the empty *E. coli* host was devoid of competing carboxylase activities.

The results are summarized in Table 1. Hydroxylated benzaldehydes, such as vanillin (**1a**) and *p*‐hydroxybenzaldehyde (**2a**) were regioselectively carboxylated by 2,3‐DHBD_Ao with 40% and 62% conversion, respectively, whereas SAD_Tm was active only on vanillin with 33% conversion (entries 1 and 2). These activities were unexpected, because carboxylation represents an electrophilic aromatic substitution, which is impeded by electron‐withdrawing substituents, such as an aldehyde.8f For instance, *o*‐ and *p*‐nitrophenol, *o*‐hydroxybenzaldehyde (see the supporting information of ref.8f) as well as isovanillin (**10a**, Supporting Information, Table S2, entry 1) were unreactive with these enzymes.

**Table 1:** Regioselective enzymatic carboxylation of (poly)phenolic substrates.

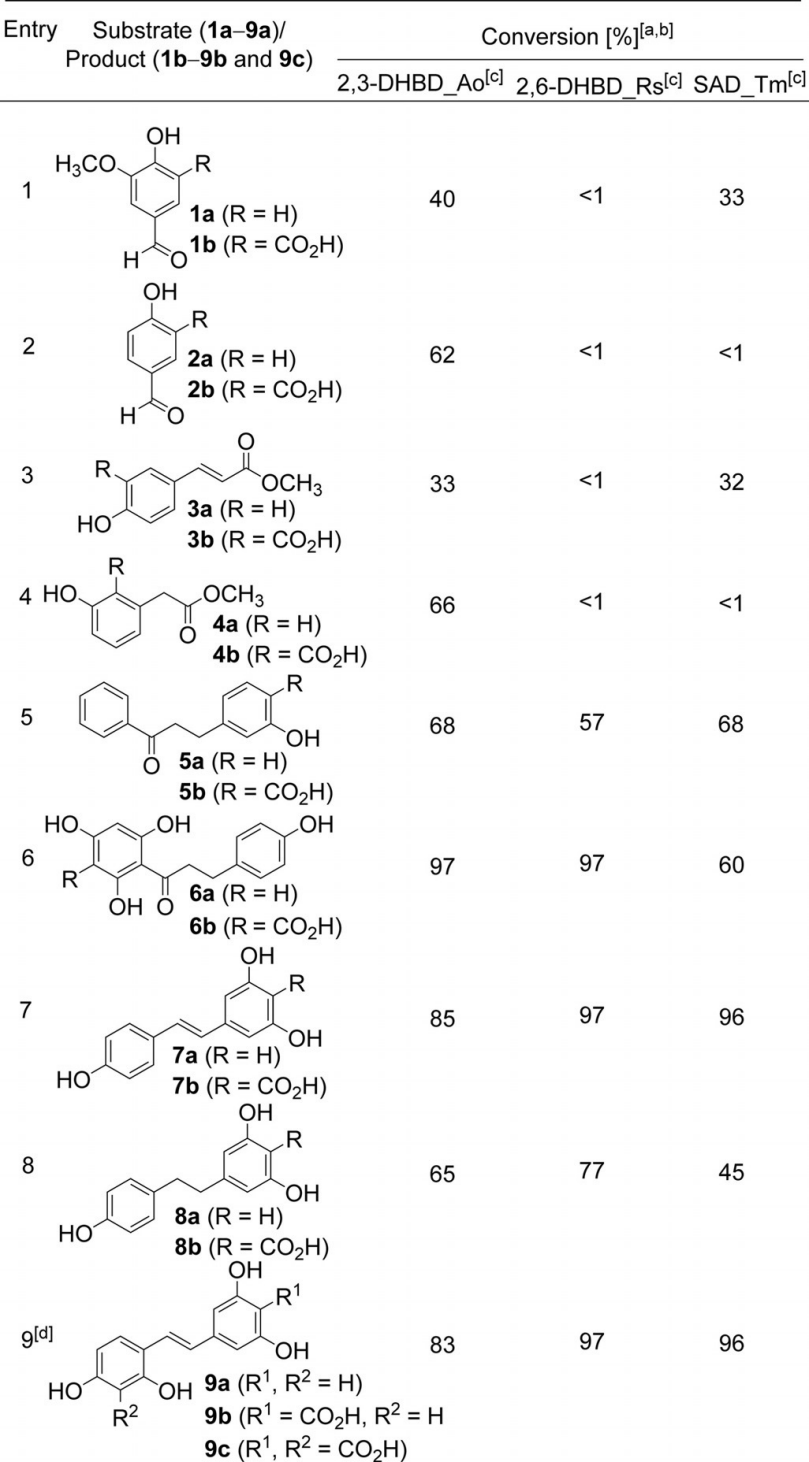

^[a]^ *Reaction conditions*: phosphate buffer (pH 8.5, 100 mM), whole lyophilized cells of *E. coli* containing the corresponding overexpressed enzyme (30 mg mL^−1^), substrate (10 mM), KHCO_3_ (3 M), 30 °C, 120 rpm, 24 h. ^[b]^ Conversions were determined by reversed‐phase HPLC, side products not detected (<2%). ^[c]^ 2,3‐DHBD_Ao=2,3‐dihydroxybenzoic acid decarboxylase from *Aspergillus oryzae*, 2,6‐DHBD_Rs=2,6‐dihydroxybenzoic acid decarboxylase from *Rhizobium* sp. and SAD_Tm=salicylic acid decarboxylase from *Trichosporon moniliiforme*. ^[d]^ Conversion corresponds to the doubly carboxylated product **9c**.

In order to convert substrates carrying a free carboxylic acid group, where *de*carboxylation would be favoured, the corresponding methyl esters were applied as masking groups. This strategy proved to be successful, as cinnamic and phenylacetic acid esters **3a** and **4a** were carboxylated by 2,3‐DHBD_Ao and SAD_Tm with up to 66% conversion (entries 3 and 4).

Surprisingly, all enzyme candidates showed significantly enhanced conversion by further expanding the complexity of the substrates containing two aromatic moieties (entries 5–9).

Substrates **5a** and **6a** of the dihydrochalcone‐type family were regioselectively carboxylated at the most electron‐rich position^25^ – even when it corresponds to a sterically hindered position like in the phloroglucinol moiety (**6a**, entry 6) – with moderate to excellent conversions, regardless of the enzyme used (conversion 57–97%, entries 5 and 6). The deactivating (−M) effect of the electron‐withdrawing carbonyl group was compensated in the presence of three hydroxy groups exerting a +M effect (**6a**, entry 6), whereas no carboxylation took place in presence of a single (**31a**, **35a**, Supporting Information, Table S2, entries 22 and 26) or two (**36a**, Supporting Information, Table S2, entry 27) OH moieties due to insufficient activation.

The well‐known phytoalexin resveratrol (**7a**) and derivatives (**8a**, **9a**) were accepted as substrates by all three enzymes (entries 7–9). Even though the steric requirements of **7a** and **8a** are almost identical, the conjugated system of **7a** enabled additional electronic activation towards carboxylation by the second aromatic moiety leading to considerably better conversions (85–97%, respectively, entry 7) compared to the non‐conjugated (saturated) resveratrol derivative **8a** (45–77 % conversion, entry 8). This is in line with the results obtained with the well accepted oxyresveratrol (**9a**), which underwent unique double carboxylation on both resorcinol units, which are similarly electronically activated. Detailed investigation over time (see the Supporting Information, Figure S7) revealed that oxyresveratrol was first *mono*‐carboxylated at the 4′ position to yield the mono‐carboxylic acid **9b**, which was subsequently converted to diacid **9c** with almost full conversion by 2,6‐DHBD_Rs and SAD_Tm (96% and 97%, respectively, entry 9). Data from a recent study8g showed that γ‐resorcylic acid decarboxylase from *Rhizobium radiobacter* WU‐0108 (95% sequence similarity to 2,6‐DHBD_Rs, Supporting Information, Figure S8) was also able to carboxylate resveratrol (**8a**).

Products **1b**–**9b** and **9c** were characterized by 1‐D (^1^H and ^13^C) and 2‐D NMR (COSY, HSQC and HMBC, see the Supporting Information) and HR‐MS.

Various other classes of substrates (such as phenol ether, heteroaromatics, coumarins, etc.), which were not accepted by any of the three decarboxylases are summarized in the Supporting Information (Table S2).

In order to demonstrate the applicability of biocatalytic carboxylation on a preparative scale, substrates **3a**, **4a** and **6a**–**9a** were subjected to up‐scaling experiments after optimization of the reaction conditions regarding the following parameters: (i) concentration of co‐substrate bicarbonate, (ii) biocatalyst loading, (iii) addition of organic co‐solvents and (iv) substrate concentration using resveratrol (**7a**) as model substrate and 2,6‐DHBD_Rs as biocatalyst (for details see the Supporting Information). Optimal parameters to reach complete conversion (≥97%) within 18–24 h were identified as 2 M bicarbonate concentration, 2 mg mL^−1^ lyophilized whole‐cell biocatalyst and 20% (v/v) of methanol as water‐miscible organic co‐solvent. For cinnamic and phenylacetic acid esters (**3a**, **4a**) moderate yields were achieved (32% and 43%, respectively, Table 2, entries 1 and 2) employing 2,3‐DHBD_Ao as biocatalyst which is in accordance with the results obtained from the screening (Table 1, entries 3 and 4). In the case of the dihydrochalcone‐type substrate (**6a**) the isolated yield of **6b** was 67% (entry 3) with 2,3‐DHBD_Ao. For the resveratrol‐like substrates (**7a**–**9a**) 2,6‐DHBD_Rs was used as biocatalyst since the latter gave the best results in the screening experiments (Table 1). Whereas for saturated resveratrol (**8a**) and oxyresveratrol (**9a**) isolated yields were moderate (45% and 66%, respectively, entries 5 and 6), resveratrol (**7a**) was carboxylated to **7b** in 95% isolated yield (entry 4). For comparison, γ‐resorcylic acid decarboxylase from *Rhizobium radiobacter* WU‐0108 furnished **7b** with only 26% yield.8g

**Table 2:** Up‐scaling of various substrates.^[a]^

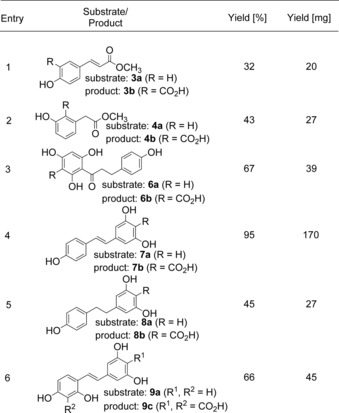

^[a]^ *Reaction conditions*: phosphate buffer (pH 8.5, 100 mM, 19 mL), whole lyophilized cells of *E. coli* containing the corresponding overexpressed enzyme [2,3‐DHBD_Ao (651 mg) for substrates **3a**, **4a** and **6a**; 2,6‐DHBD_Rs (651 mg) for substrates **7a**–**9a**], substrate (50 mg, 10–11 mM depending on the substrate) dissolved in MeOH (1 mL), KHCO_3_ (3 M), 30 °C, 400 rpm, 24 h.

In conclusion, our study shows that *o*‐benzoic acid decarboxylases are able to convert large (poly)phenolic natural products possessing antioxidant activity, such as phloretin and resveratrol, with up to quantitative conversion after optimization of the reaction conditions. On complex substrates possessing more than one potential carboxylation site, the relative electron‐density of the aromatic moieties determines the regioselectivity. Surprisingly, various electron‐withdrawing functional groups, such as carboxylic esters and aldehydes, were tolerated rather well. Overall, this study illustrates the versatility of bio‐carboxylation as a highly regioselective and synthetically useful biocatalytic alternative to the Kolbe–Schmitt reaction.

## Experimental Section

### General

Substrates **1a**, **2a**, **6a** and **8a** as well as reference material of **1b** and **2b** were purchased from Sigma Aldrich, while **7a** was obtained from Acros Chemicals. *p*‐Coumaric acid for the synthesis of substrate **4a** was provided by Roche, substrate **8a** was prepared by catalytic hydrogenation of resveratrol as previously described^26^ and provided by L'Oréal R&I. For the synthesis of substrates **3a**–**5a** and reference material for product **5b** see the Supporting Information. TLCs were run on silica plates (Merck, silica gel 60, F_254_), for column chromatography silica gel 60 Å (Merck) was used, compounds were visualized using UV (254 and 365 nm) and by spraying with cerium ammonium molybdate [5 g (CeSO_4_)_2_, 25 g (NH_4_)_6_Mo_7_O_24_⋅4 H_2_O, 50 mL concentrated H_2_SO_4_, 450 mL H_2_O]. 2,3‐Dihydroxybenzoic acid decarboxylase from *Aspergillus oryzae* (2,3‐DHBD_Ao), 2,6‐dihydroxybenzoic acid decarboxylase from *Rhizobium* species (2,6‐DHBD_Rs) and salicylic acid decarboxylase from *Trichosporon moniliiforme* (SAD_Tm) were cloned and overexpressed as previously described.^27^

### General Procedure for Biotransformations

Lyophilized whole cells (30 mg *E. coli* cells, containing the corresponding overexpressed enzyme) were rehydrated in phosphate buffer (900 μL, pH 5.5, 100 mM) for 30 min. The substrate [10 mM final concentration, dissolved in 100 μL organic co‐solvent (DMSO, MeOH and H_2_O/MeCN 50:50, respectively)] was added to the enzyme solution (1 mL final volume) which was transferred into a glass vial containing KHCO_3_ (3 M) to give a final pH of 8.5. The vials were tightly sealed with screw caps and were shaken for 18 h at 30 °C with 120 rpm. After 18 h the reaction was stopped by taking 100 μL of the reaction mixture and diluting it in 900 μL of H_2_O/MeCN/trifluoroacetic acid (TFA, 50:50:3) to precipitate the enzyme, which was removed by centrifugation (10 min, 14000 rpm). The resulting supernatant was directly used for measurements on a reversed‐phase HPLC system using H_2_O/MeCN (0.1% TFA) as eluent (gradient: MeCN/H_2_O 5%–100%) on an achiral C18 column (Phenomenex Luna, C18 100 Å, 250×4.6 mm, 5 μm). Anisole was used as an internal standard. All screening experiments were carried out at least in triplicate.

### Preparative‐Scale Biotransformation of Resveratrol (7a)

Lyophilized whole cells (651 mg *E. coli* cells, containing the corresponding expressed enzyme) were rehydrated in phosphate buffer (19 mL, pH 5.5, 100 mM) for 30 min. Substrate **7a** (50 mg in 1 mL MeOH) was added to the enzyme solution and this mixture was transferred into a glass reaction vessel containing KHCO_3_ (6.5 g, 3 M) to give a final pH of 8.5. The vessel was tightly closed with a rubber stopper (fixed with a clamp to avoid loss of CO_2_) and was allowed to react for 24 h at 30 °C with 400 rpm shaking. Overall, 150 mg of resveratrol were carboxylated in triplicate parallel experiments with 50 mg substrate each, which were combined before work‐up. After 24 h the reaction was stopped by slow addition of HCl (6 M) until a pH of around 1–2 was reached (caution: CO_2_ formation). The quenched reaction mixture was saturated with NaCl and extracted with EtOAc (4×, 10 mL) and the combined organic layers were dried with Na_2_SO_4_. EtOAc was removed under reduced pressure and remaining solids were purified using silica gel column chromatography. Silica gel was deactivated with aqueous ammonia solution, which was added to the solvent mixture used for packing of the column [CH_2_Cl_2_/MeOH 90:10 with 2% v/v of aqueous ammonia solution (30%)]. After column chromatography, solvents were removed under reduced pressure and the residue (containing the ammonium salt of **7b**) was redissolved in EtOAc (10 mL) containing HCl (6 M, 1 mL) and water (10 mL). The acidified aqueous phase was re‐extracted with EtOAc (3×10 mL), the combined organic phases were dried over Na_2_SO_4_ and the organic solvent was removed under reduced pressure to give free acid **7b** as a yellow solid; yield: 169 mg (94%).

Preparative‐scale experiments with **3a**, **4a**, **6a**, **8a** and **9a** were performed analogously with 50 mg substrate each.

### Analytics

***HPLC analysis***: HPLC/UV experiments were performed on an HPLC Agilent 1260 Infinity system with a diode array detector and a reversed phase Phenomenex Luna column C18 (100 Å, 250×4.6 mm, 5 μm, column temperature 24 °C). Conversions were determined by comparison with calibration curves for products and substrates prepared with authentic reference material. All compounds were spectrophotometrically detected at 254, 280 and 310 nm, respectively.

***Method A*** was run over 22 min with H_2_O/TFA (0.1%) as the mobile phase (flow rate 1 mL min^−1^) and a MeCN/TFA (0.1%) gradient (0–2 min 5%, 2–15 min 5–100%, 15–17 min 100%, 17–22 min 100–5%). ***Method B*** was run over 27 min with H_2_O/TFA (0.1%) as the mobile phase (flow rate 1 mL min^−1^) and a MeCN/TFA (0.1%) gradient (0–5 min 5%, 5–20 min 5–100%, 20–22 100%, 22–27 min 100–5%). ***Method C*** was run over 27 min with H_2_O/TFA (0.1%) as the mobile phase (flow rate 1 mL min^−1^) and a MeCN/TFA (0.1 %) gradient (0–2 min 5%, 2–15 min 5–60%, 15–21.5 min 60%, 21.5–23.5 min 60–100%, 23.5–27 min 100–5%). ***Method D*** was run over 22 min with MeOH/acetic acid (2%) as the mobile phase (flow rate 1 mL min^−1^) and a MeCN/acetic acid (2%) gradient (0–16 min 20–65%, 16–22 min 65–20%). Retention times for all substrates (**1a‐**‐**9a**) and products (**1b**–**9b** and **9c**) are listed in the Supporting Information (Table S1).

***NMR and HR‐MS analysis***: NMR spectra were recorded with a Bruker AVANCE III 300 MHz spectrometer using a 5 mm BBO probe at 300 K. Chemical shifts *δ* are expressed in ppm, coupling constants *J* are given in Hz. HR‐MS analysis was performed on an Agilent 1260 Infinity system coupled with an Agilent 6230 TOF LC/MS instrument with an APCI‐ionisation in positive mode.

**3a**: ^1^H NMR (300 MHz, DMSO‐*d_6_*): *δ*=10.02 (s, 1 H), 7.54–7.59 (m, 3 H), 6.80 (s, 1 H), 6.78 (s, 1 H), 6.40 (d, *J*=16.0 Hz, 1 H), 3.69 (s, 3 H);^28^ ^13^C NMR (75 MHz, DMSO‐*d*_6_): *δ*=167.1, 159.9, 144.8, 130.3, 125.1, 115.8, 113.9, 51.2.^29^

**3b**: ^1^H NMR (300 MHz, DMSO‐*d_6_*): *δ*=7.57 (d, *J*=7.8 Hz, 1 H), 7.53 (s, 1 H), 6.81 (s, 1 H), 6.78 (t, *J*=1.9 Hz, 1 H), 6.39 (d, *J*=16.0 Hz, 1 H), 3.69 (s, 3 H); ^13^C NMR (75 MHz, DMSO‐*d_6_*): *δ*=175.8, 167.1, 159.9, 144.8, 132.6, 130.4, 125.1, 115.8, 115.0, 113.9, 45.6.^30^

**4a**: ^1^H NMR (300 MHz, DMSO‐*d_6_*): *δ*=9.37 (bs, OH), 7.09 (t, *J*=7.09 Hz 1 H), 6.66 (s, 2 H), 6.63–6.66 (m, 1 H), 3.60 (s, 3 H), 3.56 (s, 2 H);^31^ ^13^C NMR (75 MHz, DMSO‐*d_6_*): *δ*=171.6, 157.3, 135.6, 129.3, 119.9, 116.2, 113.8, 51.7, 40.2.^32^

**4b**: ^1^H NMR (300 MHz, DMSO‐*d_6_*): *δ*=7.51 (d, *J*=7.7 Hz, 1 H), 6.52 (s, 1 H), 6.48 (dd, *J*=7.9, 1.2 Hz, 1 H), 3.19 (s, 3 H), (CH_2_ signal obscured by water peak 3.43 ppm); ^13^C NMR (75 MHz, DMSO‐*d_6_*): *δ*=172.1, 163.3, 162.3, 142.1, 129.3, 117.7, 117.5, 116.5, 67.3, 45.1.^33^

**5a**: ^1^H NMR (300 MHz, CDCl_3_) *δ*=7.92–7.99 (m, 2 H), 7.53–7.59 (m, 1 H), 7.41–7.49 (m, 2 H), 7.16 (t, *J*=7.8 Hz, 1 H), 6.81 (d, *J*=7.6 Hz, 1 H), 6.74–6.77 (m, 1 H), 6.70 (dd, *J*=8.0, 2.0 Hz, 1 H), 3.30 (dd, *J*=8.4, 6.9 Hz, 2 H), 3.02 (t, *J*=7.7 Hz, 2 H);^34^ ^13^C NMR (75 MHz, CDCl_3_) *δ*=199.9, 156.0, 143.2, 136.8, 133.4, 129.9, 128.8, 128.2, 120.8, 115.6, 113.3, 40.4, 30.1.

**5b**: ^1^H NMR (300 MHz, CDCl_3_) *δ*=7.99–7.94 (m, 2 H), 7.82 (d, *J*=8.1 Hz, 1 H), 7.58 (ddd, *J*=7.4, 3.9, 1.3 Hz, 1 H), 7.50–7.43 (m, 2 H), 6.87 (s, 1 H), 6.82 (dd, *J*=8.0, 1.2 Hz, 1 H), 3.32 (t, *J*=7.5 Hz, 2 H), 3.07 (t, *J*=7.6 Hz, 2 H); ^13^C NMR (75 MHz, CDCl_3_) *δ*=204.3, 168.4, 162.4, 145.3, 141.1, 133.4, 131.1, 128.8, 128.2, 120.1, 117.1, 117.0, 112.8, 30.3, 22.8. HR‐MS (ESI^+^): *m/z*=269.081932, calculated for C_16_H_14_O_4_ [M+H]^+^: 269.081739.

**6b**: ^1^H NMR (300 MHz, DMSO‐*d_6_*): *δ*=7.0 (d, *J*=8.4 Hz, 2 H), 6.7 (d, *J*=8.4 Hz, 2 H), 5.5 (s, 1 H), 3.3 (s, 2 H), 2.73–2.78 (m, 2 H); ^13^C NMR (75 MHz, DMSO‐*d_6_*): *δ*=204.1, 170.3, 169.5, 168.1, 155.3, 131.7, 129.2, 115.1, 103.1, 96.0, 92.6, 45.0, 29.4; HR‐MS (ESI^+^): *m/z*=317.066416, calculated for C_16_H_14_O_7_ [M+H]^+^: 317.066676.

**7b**: ^1^H NMR (300 MHz, DMSO‐*d_6_*): *δ*=7.45 (d, *J*=8.6 Hz, 2 H), 7.19 (d, *J*=16.4 Hz, 1 H), 6.87 (d, *J*=16.4 Hz, 1 H), 6.78 (d, *J*=8.6 Hz, 2 H), 6.55 (s, 2 H), broad signal from OH‐moieties overlapping with signals from 7.45–6.87); ^13^C NMR (75 MHz, DMSO‐*d_6_*): *δ*=172.9, 161.4, 158.3, 144.3, 131.9, 129.0, 128.0, 124.6, 116.0, 104.9, 100.8; HR‐MS (ESI^+^): *m/z*=271.061226, calculated for C_15_H_12_O_5_ [M+H]^+^: 271.061197.

**8b**: ^1^H NMR (300 MHz, DMSO‐*d_6_*): *δ*=6.99 (d, *J*=8.5 Hz, 2 H), 6.65 (d, *J*=8.4 Hz, 2 H), 6.23 (s, 2 H), 2.69–2.71 (m, 4 H); ^13^C NMR (75 MHz, DMSO‐*d_6_*): *δ*=172.8, 161.0, 155.8, 150.3, 131.7, 129.6, 115.4, 107.6, 100.0, 38.8, 35.7; HR‐MS (ESI^+^): *m/z*=275.09158, calculated for C_15_H_14_O_5_ [M+H]^+^: 275.09140.

**9b**: ^1^H NMR (300 MHz, DMSO‐*d_6_*): *δ*=7.37 (d, *J*=8.5 Hz, 1 H), 6.87 (d, *J*=8.9 Hz, 1 H), 6.80 (t, *J*=8.2 Hz, 1 H), 6.41 (s, 2 H), 6.33 (d, *J*=2.4 Hz, 1 H), 6.27–6.23 (m, 1 H). ^13^C NMR (75 MHz, DMSO‐*d_6_*): *δ*=172.9, 161.2, 160.8, 158.8, 145.9, 129.7, 126.4, 125.5, 115.0, 107.4, 104.0, 103.8, 103.7, 102.6.

**9c**: ^1^H NMR (300 MHz, DMSO‐*d_6_*): *δ*=7.58 (d, *J*=8.7 Hz, 1 H), 7.35 (d, *J*=16.5 Hz, 1 H), 6.95 (d, *J*=16.5 Hz, 1 H), 6.51 (s, 2 H), 6.30 (t, *J*=9.2 Hz, 1 H); ^13^C NMR (75 MHz, DMSO‐*d_6_*): *δ*=174.5, 172.5, 162.5, 161.2, 160.8, 145.3, 132.0, 126.9, 124.1, 114.6, 107.3, 104.7, 103.0, 100.3; HR‐MS (ESI^+^): *m/z*=333.060595, calculated for C_16_H_14_O_8_ [M+H]^+^: 333.060494.

## Supporting information

As a service to our authors and readers, this journal provides supporting information supplied by the authors. Such materials are peer reviewed and may be re‐organized for online delivery, but are not copy‐edited or typeset. Technical support issues arising from supporting information (other than missing files) should be addressed to the authors.

Supplementary
