# Supplementary material for: Regioselective Enzymatic Carboxylation of Bioactive (Poly)phenols
Source: Adv Synth Catal. 2017 Jan 18;359(6):959–65. doi: 10.1002/adsc.201601046 (PMC5396361; doi:10.1002/adsc.201601046)
Supplement: Supplementary file 1 — Supplementary [file ADSC-359-959-s001.pdf]

Advanced Synthesis & Catalysis

Supporting Information

Regioselective Enzymatic Carboxylation of Bioactive (Poly)phenols

Katharina Plasch,[a] Verena Resch,[a] Julien Hitce,[b] Jarosław Popłoński,[c] Kurt Faber,[a]* Silvia M. Glueck[a,d]*

[a] Department of Chemistry, Organic & Bioorganic Chemistry, University of Graz, Heinrichstrasse 28, A-8010 Graz, Austria

Fax: +43 (0)316 380 9840; Email: Si.Glueck@Uni-Graz.at, Kurt.Faber@Uni-Graz.at

[b] L'Oréal Research & Innovation, 30 bis rue Maurice Berteaux, 95500 Le Thillay, France

[c] Department of Chemistry, Wrocław University of Environmental and Life Sciences, ul. C. K. Norwida 25, 50-375 Wrocław, Poland

[d] Austrian Centre of Industrial Biotechnology (ACIB) c/o University of Graz, Heinrichstrasse 28, A-8010 Graz, Austria

Supplementary Material for Review

1. Enzymes 2

2. Engineering of reaction parameters 2

    2.1. General optimization procedure 2

    2.2 Bicarbonate concentration study 3

    2.3. Optimization of enzyme concentration 4

    2.4 Evaluation of organic co-solvents 4

    2.5. Substrate concentration study 5

    2.6. Time study 6

3. Time study of the bio-carboxylation of **9a** 6

4. Synthesis of substrates and reference material 7

5. Sequence alignment 9

6. HPLC analysis 10

7. NMR Spectra and HR-MS 13

8. References 52

1. Enzymes

Three different *ortho*-benzoic acid decarboxylases were applied in this study:

- 2,3-Dihydroxybenzoic acid decarboxylase from *Aspergillus oryzae* (2,3-DHBD_Ao)
- 2,6-Dihydroxybenzoic acid decarboxylase from *Rhizobium* sp. (2,6-DHBD_Rs) and
- Salicylic acid decarboxylase from *Trichosporon moniliiforme* (SAD_Tm)

The cloning and overexpression was performed as previously reported.[1] Successful overexpression of soluble and active enzymes was obtained for all decarboxylases (Fig. S1). The activity was tested with standard screening procedure using orcinol as substrate.

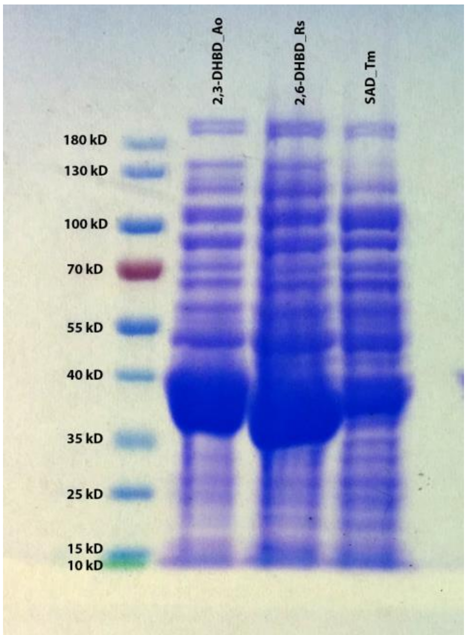

Fig. S1. SDS-PAGE analysis of heterologously expressed decarboxylases.

2. Engineering of reaction parameters

In order to optimize conversions in preparative-scale experiments, optimization studies of various reaction parameters were performed.

2.1. General optimization procedure

Lyophilized whole *E. coli* cells containing overexpressed 2,6-dihydroxybenzoic acid decarboxylase from *Rhizobium* sp. (2,6-DHBD_Rs, 30 mg mL^{-1}) were rehydrated in phosphate buffer (900 µL, pH 5.5, 100 mM) for 30 minutes. The model substrate [resveratrol (**7a**), 10 mM final concentration, dissolved in MeOH (100 µL, 10% v/v)] was added to the enzyme solution (1 mL final volume) which was afterwards transferred into a glass vial containing $KHCO_3$ (2 M) to give a final pH of 8.5. The vials were tightly sealed with screw caps and samples were shaken for 18 h at 30 °C with 120 rpm. After 18 h the reaction was stopped by taking 100 µL of the reaction mixture and diluting

it in 900 µL of H_2O/MeCN/TFA (50:50:3) to precipitate the enzyme, which was removed by centrifugation (10 min, 14000 rpm). The resulting supernatant was directly used for measurement. Samples were analyzed on a reversed-phase HPLC system using H_2O/MeCN (0.1% TFA) as eluent (gradient: MeCN:H_2O 5%–100%). Samples were measured on the achiral C18 column Phenomenex Luna C18 (100 Å, 250 x 4.6 mm, 5 µm, column temperature 24 °C). Anisole was used as an internal standard. For the optimization of bicarbonate concentration various concentration of $KHCO_3$ (0.5 M, 1 M, 2 M, 3 M) were applied (see Fig. S2).

For the evaluation of the required enzyme concentration various amounts of biocatalyst (30, 20, 10, 5, 2, 1, 0.5 mg mL^{-1}) were applied (see Fig. S3).

For the evaluation of co-solvents the standard conditions were supplemented by addition of various water-miscible and water-immiscible organic co-solvents (MeOH, EtOH, THF, MeCN, *iso*-propanol, *tert*-butanol, 1,4-dioxane, 1-butanol, acetone, butanone, toluene, *n*-heptane, cyclohexane, *tert*-butyl methyl ether and ethyl acetate) at various concentrations (10%, 20% 30% v/v, see Fig. S4).

In order to optimize the substrate concentration, the following concentrations were used (10, 30, 50, 100, 150, 200 mM, see Fig. S5).

In order to follow the biotransformation over time samples were taken after 1, 2, 4, 6, 10, 16, 20 and 24 h (see Fig. S6).

2.2 Bicarbonate concentration study

In order to achieve full conversion within 18 h, a bicarbonate concentration of at least 2 M is required. When lowering the amount, the reaction was slowed down. However, carboxylation was still observed even at a bicarbonate concentration of 0.5 M (see Fig. S2).

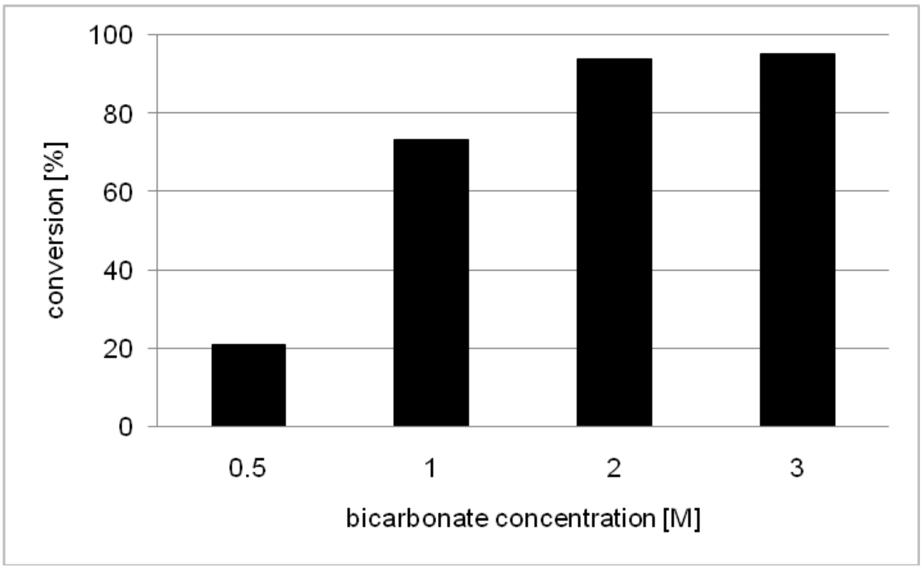

Fig. S2. Dependence of bicarbonate concentration on the *ortho*-carboxylation of resveratrol (**7a**) employing 2,6-DHBD_Rs as biocatalyst.

2.3. Optimization of enzyme concentration

The evaluation of the biocatalyst concentration showed that already 2 mg mL^{-1} of lyophilized whole cell preparation was enough to achieve full conversion within 18 h. The enzyme loading was decreased by a factor >10 in comparison to standard substrate screening conditions. (see Fig. S3).

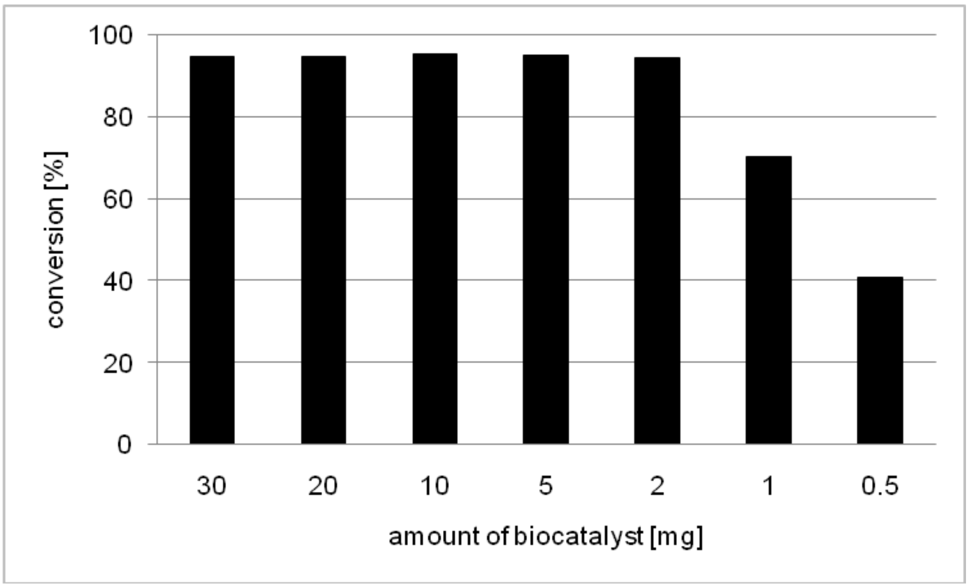

Fig. S3. Dependence of amount of biocatalyst on the *ortho*-carboxylation of resveratrol (**7a**) employing 2,6-DHBD_Rs as biocatalyst.

2.4 Evaluation of organic co-solvents

In order to enhance the solubility of the substrate a range of water-miscible and water-immiscible organic co-solvents were tested using 10%, 20% and 30% v/v, respectively (see Fig. S4). All water-miscible solvents were well accepted by the biocatalyst, the only exception being THF which caused enzyme deactivation already at a concentration of 10% v/v. In case of water-immiscible solvents only toluene had a beneficial effect compared to the buffer blank without co-solvent which was, however, significantly lower as in case of all water-miscible candidates (except THF). The low conversions when applying water-immiscible solvents is mainly attributed to the low solubility of resveratrol rather than enzyme inactivation. Best conversion was obtained with MeOH, which was thus employed in upscale experiments.

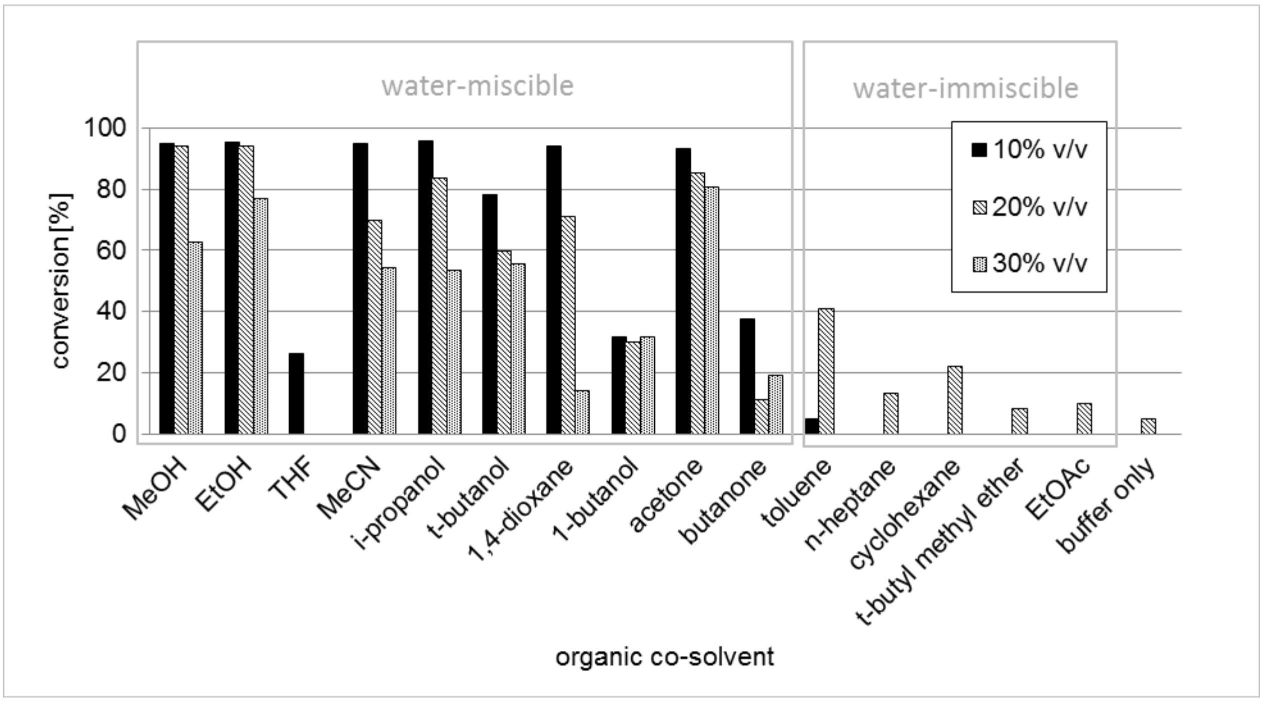

Fig. S4. Evaluation of organic co-solvents when applied in the *ortho*-carboxylation of resveratrol (**7a**) employing 2,6-DHBD_Rs as biocatalyst.

2.5. Substrate concentration study

The evaluation of the substrate concentration showed that a substrate loading of 50 mM still led to full conversion within 18 h which is a five-fold improvement compared to the standard substrate screening conditions. A further increase of substrate loading resulted in a continuous decrease in conversion. It is worth to mention that already 100 mM of resveratrol is not fully soluble in 20% v/v MeOH.

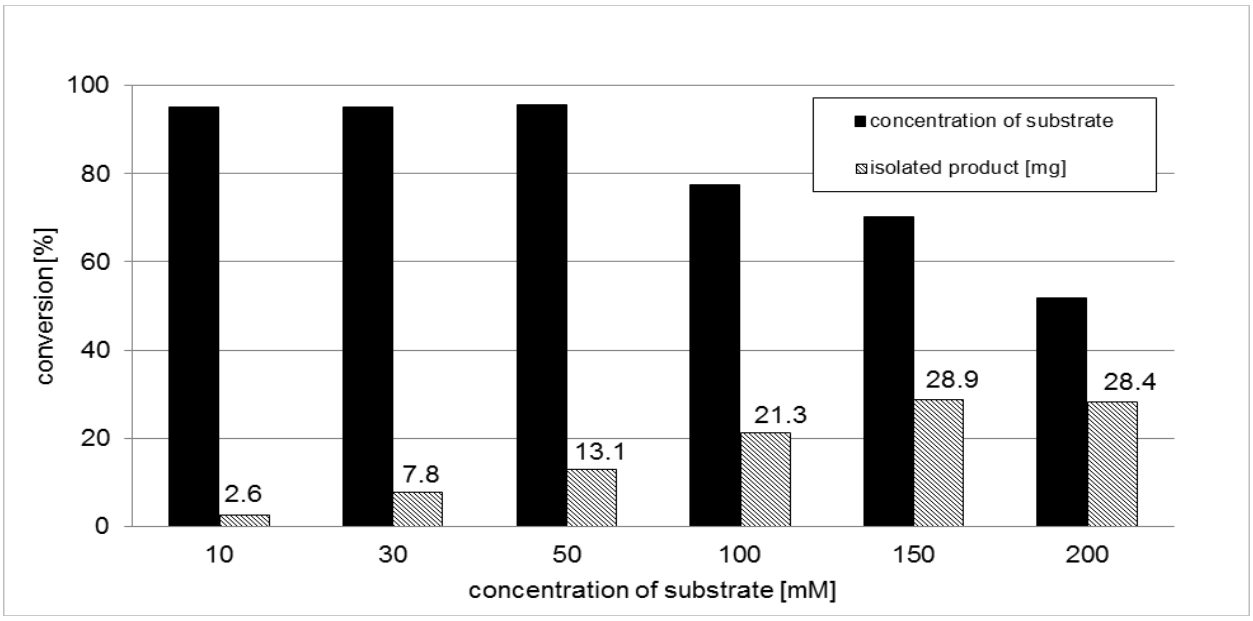

Fig. S5. Dependence of initial substrate concentration on the *ortho*-carboxylation of resveratrol (**7a**) employing 2,6-DHBD_Rs as biocatalyst.

2.6. Time study

The course of the biotransformation under optimized conditions (2 M bicarbonate, 2 mg mL^{-1} lyophilized whole cell biocatalyst, 20% (v/v) MeOH) was monitored over time showing a continuous increase of conversion with proceeding reaction time up to completion (97%) after 24 h.

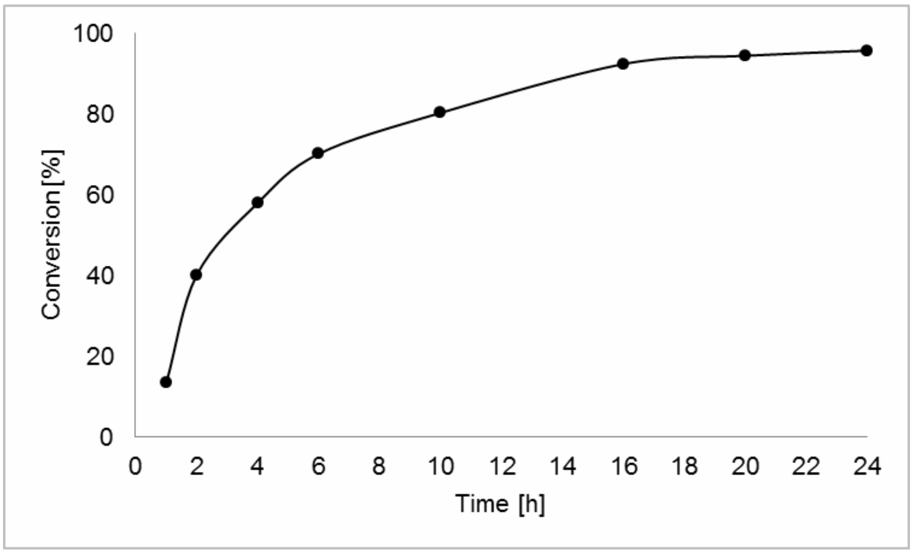

Fig. S6. Reaction progress of the bio-carboxylation over time using resveratrol (**7a**) as model substrate and 2,6-DHBD_Rs as biocatalyst under optimized conditions.

3. Time study of the bio-carboxylation of 9a

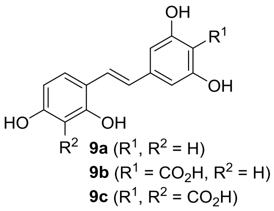

The bio-carboxylation of oxyresveratrol (**9a**) was monitored over time. While at the beginning of the reaction (first 15 minutes) the mono-carboxylated compound (**9b**) accumulated, a continuous decrease was observed during progress of the reaction whereas the doubly carboxylated compound (**9c**) occurred after 15 min and steadily increased until the reaction was stopped (after 24 h).

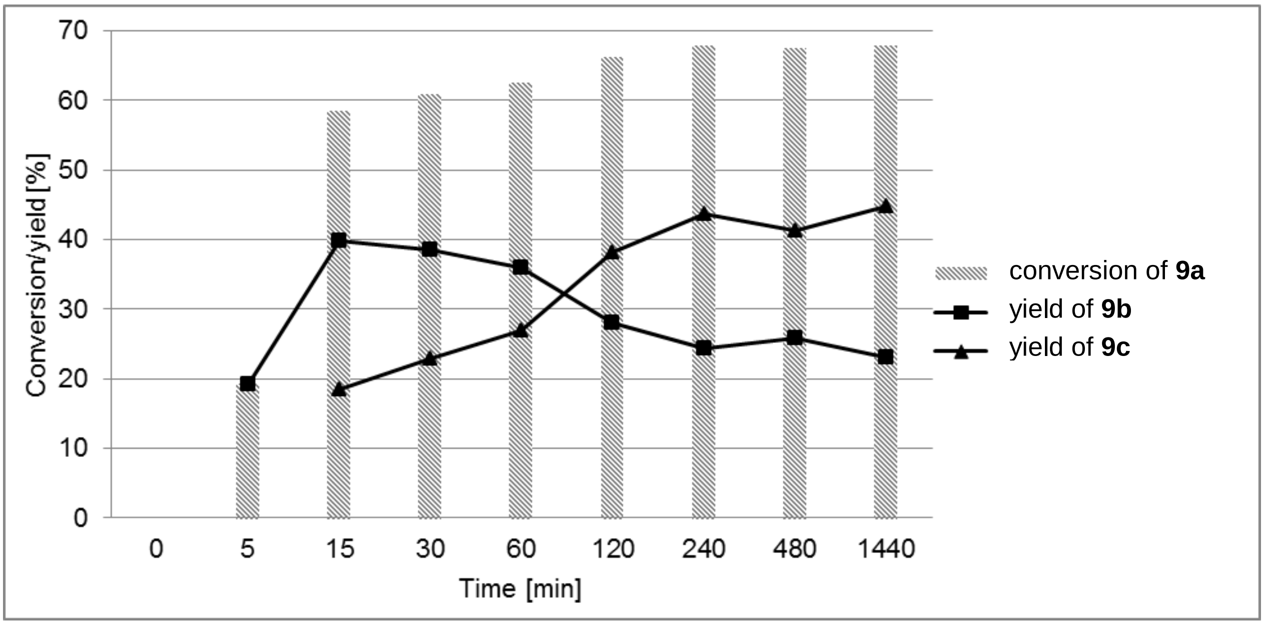

Fig. S7. Reaction process of the mono- and double carboxylation over time using oxyresveratrol (**9a**) as substrate (10 mM in 10% v/v MeOH) and 2,6-DHBD_Rs as biocatalyst (30 mg mL^{-1}) with standard screening conditions [$KHCO_3$ (3 M), phosphate buffer (pH 8.5, 100 mM), 30 °C, 120 rpm, 24 h].

4. Synthesis of substrates and reference material

Methyl (*E*)-3-(4-hydroxyphenyl)acrylate **3a**[2] and methyl 2-(3-hydroxyphenyl)acetate **4a**:[3] *p*-Coumaric acid **3** (1 g, 1.0 eq, 6.09 mmol) and 3-hydroxyphenylacetic acid **4** (1 g, 1.0 eq, 6.57 mmol), respectively were dissolved in MeOH (15 mL) supplemented with H_2SO_4 (conc., 2 drops). The reaction mixture was stirred at 60 °C. After the reaction was completed (after 3 days, monitored by TLC: cyclohexane/EtOAc 1:2, 254 nm), the solvent was removed under reduced pressure. The crude product was dissolved in EtOAc (10 mL) and washed with sat. $NaHCO_3$ (2 x 10 mL). The organic phases were combined, washed with brine (30 mL) and afterwards dried over $MgSO_4$ to yield either white crystals of **3a** (0.86 g, 79%,) or a clear yellowish solution of **4a** (0.70 g, 64%).

3a: ^{1}H NMR (300 MHz, DMSO-d_6): δ = 10.02 (s, 1H), 7.54-7.59 (m, 3H), 6.80 (s, 1H), 6.78 (s, 1H), 6.40 (d, *J* = 16.0 Hz, 1H), 3.69 (s, 3H); ^{13}C NMR (75 MHz, DMSO-d_6) δ = 167.1, 159.9, 144.8, 130.3, 125.1, 115.8, 113.9, 51.2.[2]

4a: ^{1}H NMR (300 MHz, DMSO-d_6): δ = 9.37 (bs, OH), 7.09 (t, J = 7.09 Hz 1H), 6.66 (s, 2H), 6.63-6.66 (m, 1H), 3.60 (s, 3H), 3.56 (s, 2H); ^{13}C NMR (75 MHz, DMSO-d_6) δ = 171.6, 157.3, 135.6, 129.3, 119.9, 116.2, 113.8, 51.7, 40.2.[3]

3-Hydroxydihydrochalcone **5a** was obtained in a two-step synthesis: *Step 1*: Acetophenone (0.6 g, 5 mmol) and *m*-hydroxybenzaldehyde (0.61 g, 5 mmol) were dissolved in MeOH (15 mL) followed

by the addition of methanolic KOH solution (10%, 10 mL). The reaction was stirred for 2 days at room temperature and afterwards poured into ice-cold HCl solution (10%, 100 mL). The precipitate formed was washed with a small portion of ice-cold EtOH and water, chromatographed over silica gel and recrystallized from aq EtOH to give 416 mg (37%) of 3-hydroxychalcone (**5**).[4]

5: ^{1}H NMR (601 MHz, Acetone-d$_6$): δ = 8.57 (s, 1H), 8.14 (dt, *J* = 8.4, 1.6 Hz, 2H), 7.79 (d, *J* = 15.6 Hz, 1H), 7.72 (d, *J* = 15.6 Hz, 1H), 7.62 – 7.66 (m, 1H), 7.54 – 7.58 (m, 2H), 7.27 – 7.32 (m, 3H), 6.95 (ddd, *J* = 7.5, 2.4, 1.6 Hz, 1H);[5] ^{13}C NMR (151 MHz, Acetone-d$_6$): δ = 190.0, 158.7, 145.0, 139.1, 137.4, 133.6, 130.8, 129.5, 129.4, 129.3, 128.8, 122.9,121.0, 118.5, 115.9.

Step 2: 3-Hydroxychalcone (112 mg, 0,5 mmol) was dissolved in MeOH (10 mL) and 5% Pd/C catalyst (56 mg) was added. The mixture was bubbled with N_2 for 5 min and 3 min with H_2. The catalyst was filtered off, the reaction was concentrated in vacuo and chromatographed over silica gel to give 71 mg (67 %) of the product **5a**.[4]

5a: ^{1}H NMR (300 MHz, $CDCl_3$): δ = 7.94 – 7.97 (m, 2H), 7.53 – 7.59 (m, 1H), 7.42 – 7.48 (m, 2H), 7.16 (t, *J* = 7.8 Hz, 1H), 6.81 (d, *J* = 7.6 Hz, 1H), 6.75 – 6.76 (m, 1H), 6.70 (dd, *J* = 8.0, 2.0 Hz, 1H), 5.48 (bs, 1H), 3.30 (dd, *J* = 8.4, 6.9 Hz, 2H), 3.02 (t, *J* = 7.7 Hz, 2H);[5] ^{13}C NMR (75 MHz, $CDCl_3$): δ = 199.6, 156.0, 143.2, 136.8, 133.4, 129.9, 128.8, 128.2, 120.8, 115.6, 113.3, 40.4, 30.1.

5. Sequence alignment

The sequence alignment of 2,6-dihydroxybenzoic acid decarboxylase from *Rhizobium* sp. (NCBI Reference GI: 116667102)[6,7,8] with γ-resorcylic acid decarboxylase from *Rhizobium radiobacter* WU-0108 (NCBI Reference GI: 54290091)[6,9] showed a similarity of 98%. The alignment was performed with BLAST NCBI Protein and CLC Main Workbench 7.03.

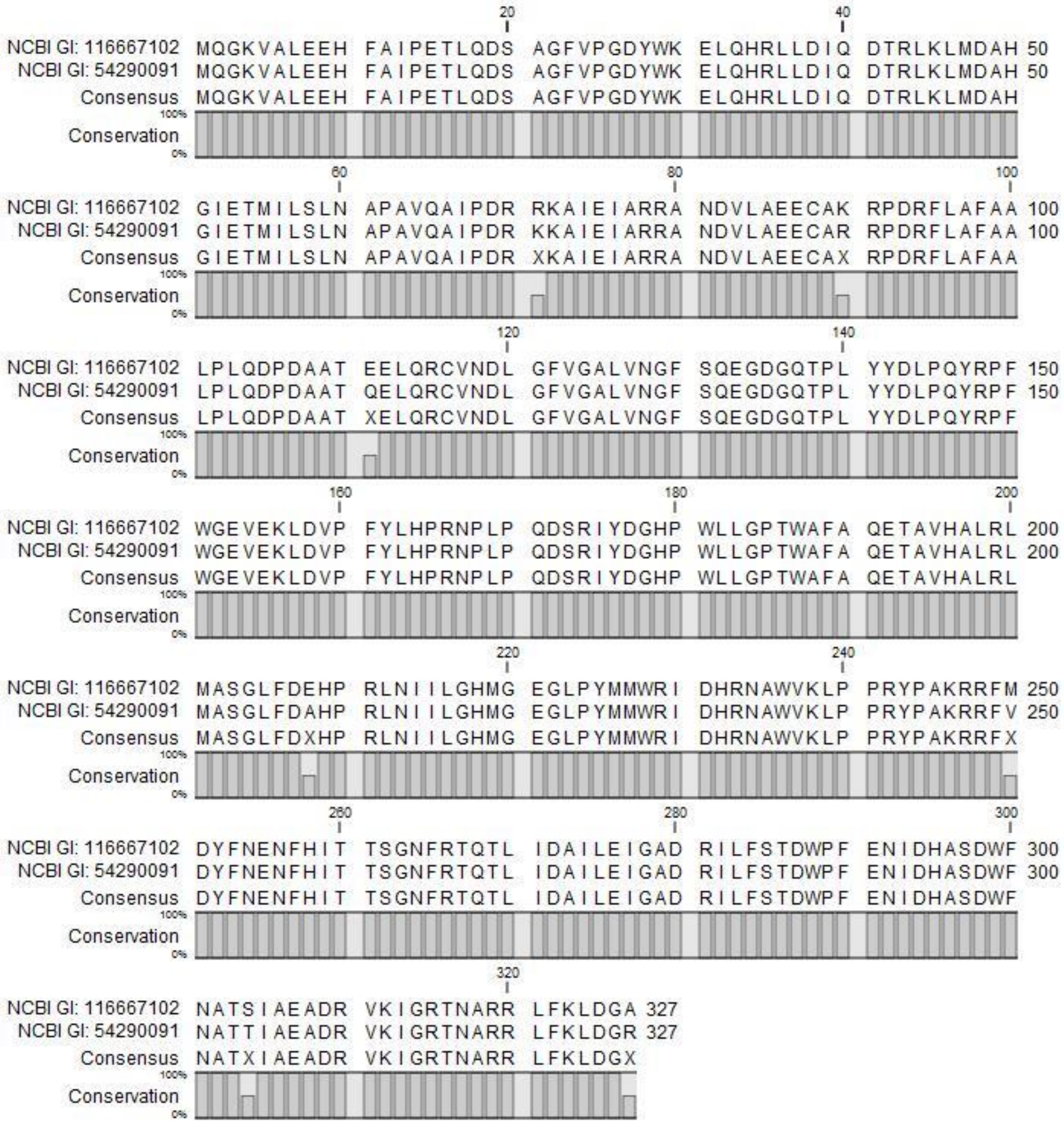

Fig. S8. Sequence alignment of enzymes.

6. HPLC analysis

Table S1. HPLC analysis of substrates (**1a - 9a**) and products (**1b** – **9b** and **9c)**.

substrates (**1a - 9a**)/ products (**1b - 9b**, **9c**)	HPLC method	retention time [min]	
		substrate (**1a** – **9a**)	product (**1b** – **9b**, **9c)**
**1a/1b**	A	10.6	10.0
**2a/2b**	A	10.3	11.1
**3a/3b**	A	12.6	12.9
**4a/4b**	A	10.1	12.1
**5a/5b**	C	15.8	16.2
**6a/6b**	C	13.6	14.0
**7a/7b**	B	13.2	14.3
**8a/8b**	B	13.3	14.4
**9a/9b, 9c**	B	14.3	**9b** = 15.2<br>**9c** = 15.7

Table S2. HPLC analysis of non-substrates **(10a - 39a)**.

entry	non-substrates (**10a – 39a**) conversion <1%		HPLC method	retention time [min]
1	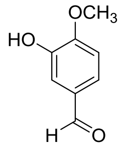	**10a**	A	10.5
2	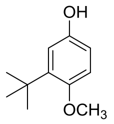	**11a**	A	15.1
3	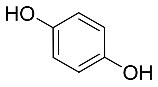	**12a**	A	7.1
4	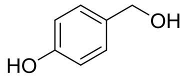	**13a**	A	9.2
5	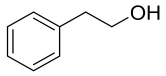	**14a**	A	11.7
6	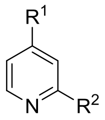	**15a** (R^1 = OH, R^2 = H)	A	14.6
7		**16a** (R^1 = H, R^2 = OH)	A	14.6
8	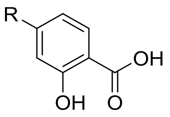	**17a** (R = H)	B	11.9
9		**18a** (R = OH)	B	10.0
10	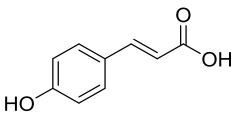	**19a**	A	10.5
11	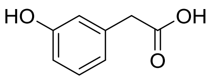	**20a**	A	10.5
12	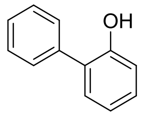	**21a**	A	14.5
13	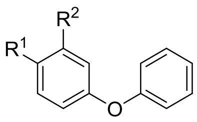	**22a** (R^1 = OH, R^2 = H)	A	11.4
14		**23a** (R^1 = H, R^2 = OH)	A	11.7
15	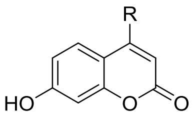	**24a** (R = H)	A	10.9
16		**25a** (R = CH_3)	A	11.6
17	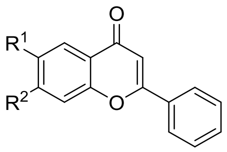	**26a** (R^1 = OH, R^2 = H)	A	13.5
18		**27a** (R^1 = H, R^2 = OH)	A	13.1

19	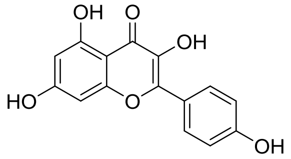	**28a**	B	12.6
20		**29a** (R^1 = OH, R^2, R^3 = H)	A	14.4
21	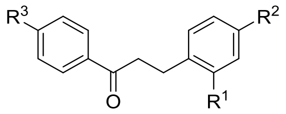	**30a** (R^1=H, R^2=OH; R^3=H)	A	13.8
22		**31a** (R^1, R^2 = H, R^3 = OH)	A	14.1
23		**32a** (R^1=OH, R^2, R^3, R^4=H)	A	13.5
24	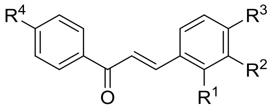	**33a** (R^1=H; R^2=OH, R^3,R^4=H)	A	14.1
25		**34a** (R^1,R^2=H, R^3=OH, R^4=H)	A	13.9
26		**35a** (R^1, R^2, R^3 = H, R^4 = OH)	A	14.0
27	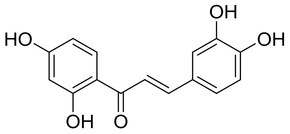	**36a**	C	13.5
28	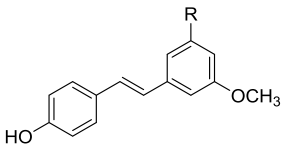	**37a** (R = OCH_3)	B	18.2
29		**38a** (R = OH)	B	14.3
30	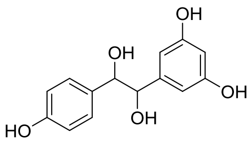	**39a**	B	8.3

7. NMR Spectra and HR-MS

Methyl (*E*)-3-(4-hydroxyphenyl)acrylate **3a**[2]

^{1}H NMR:

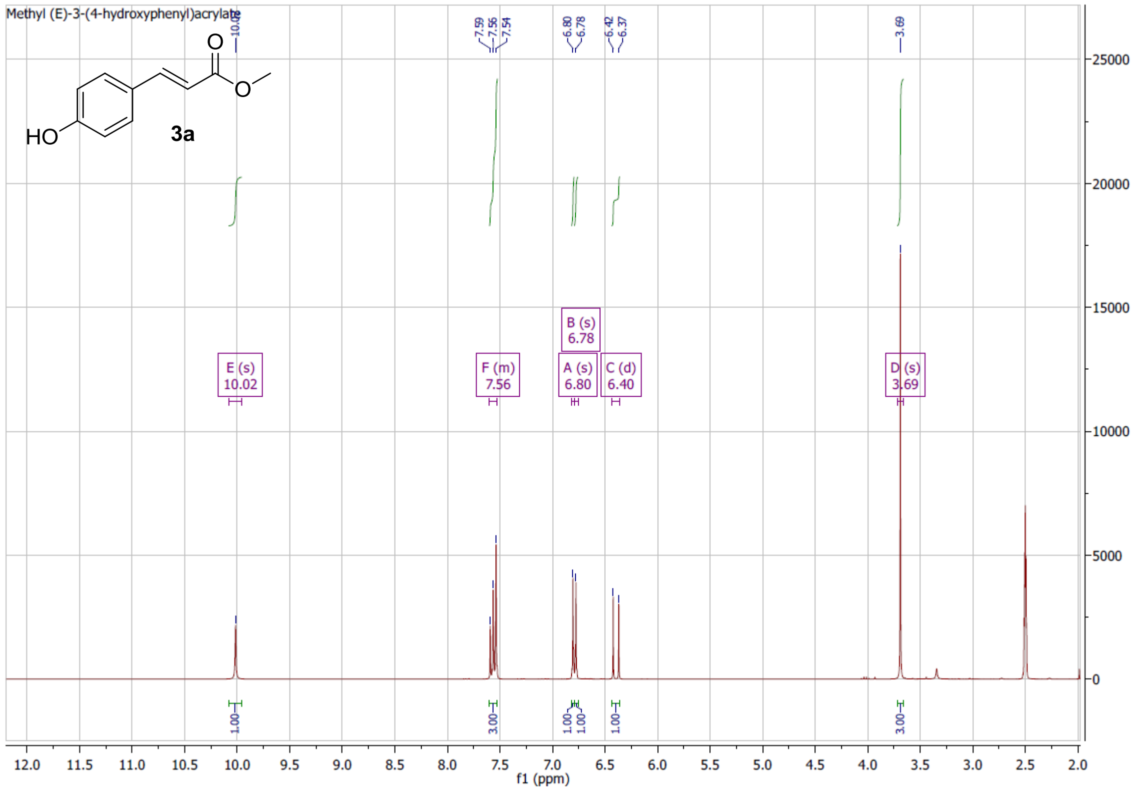

^{13}C NMR:

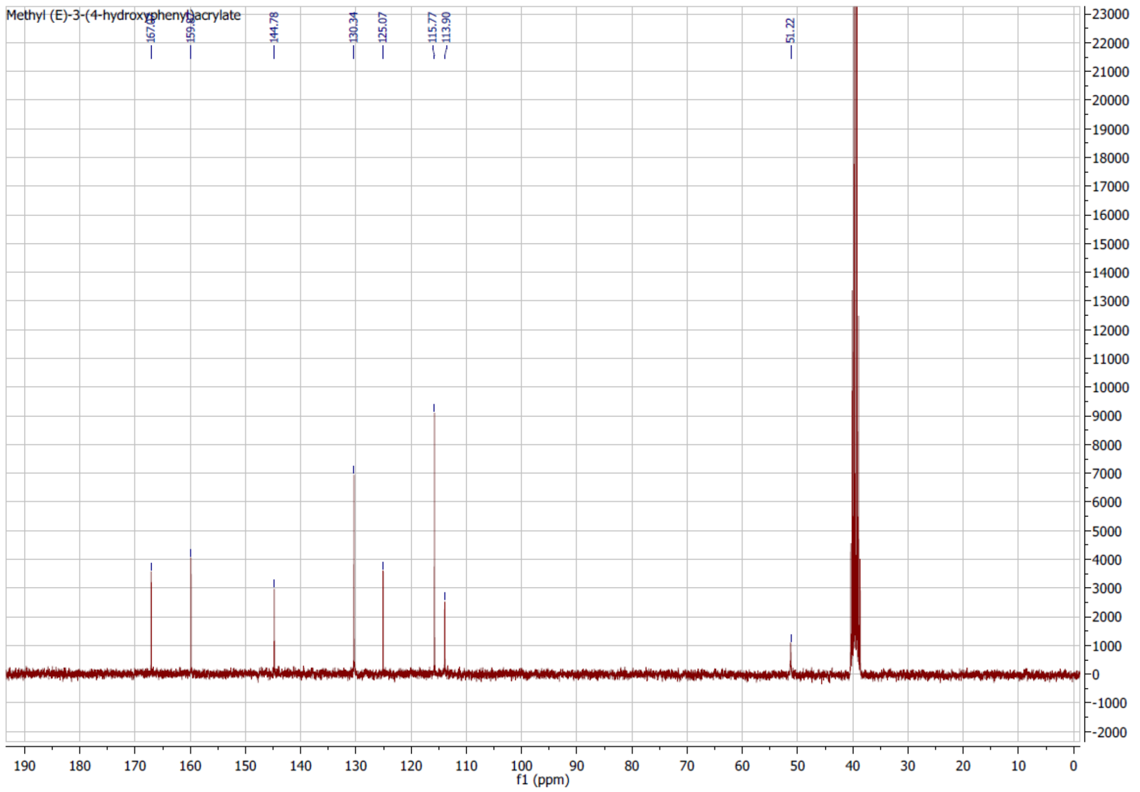

(*E*)-2-Hydroxy-5-(3-methoxy-3-oxoprop-1-en-1-yl)benzoic acid **3b**[10]

^{1}H NMR:

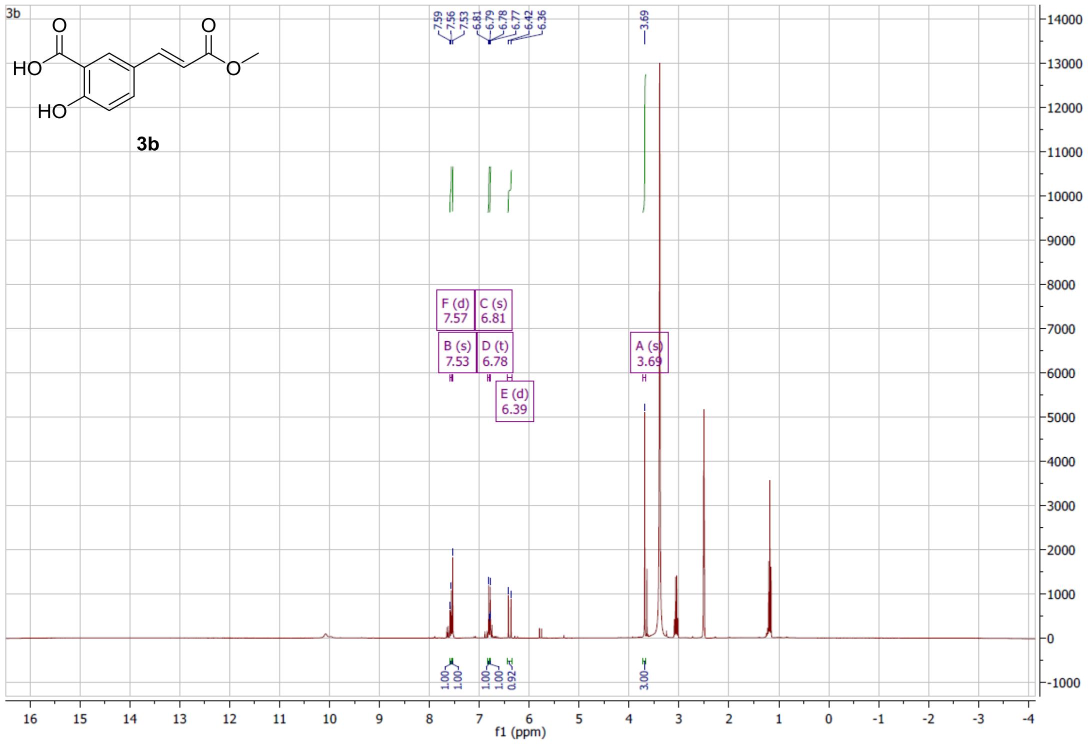

^{13}C NMR:

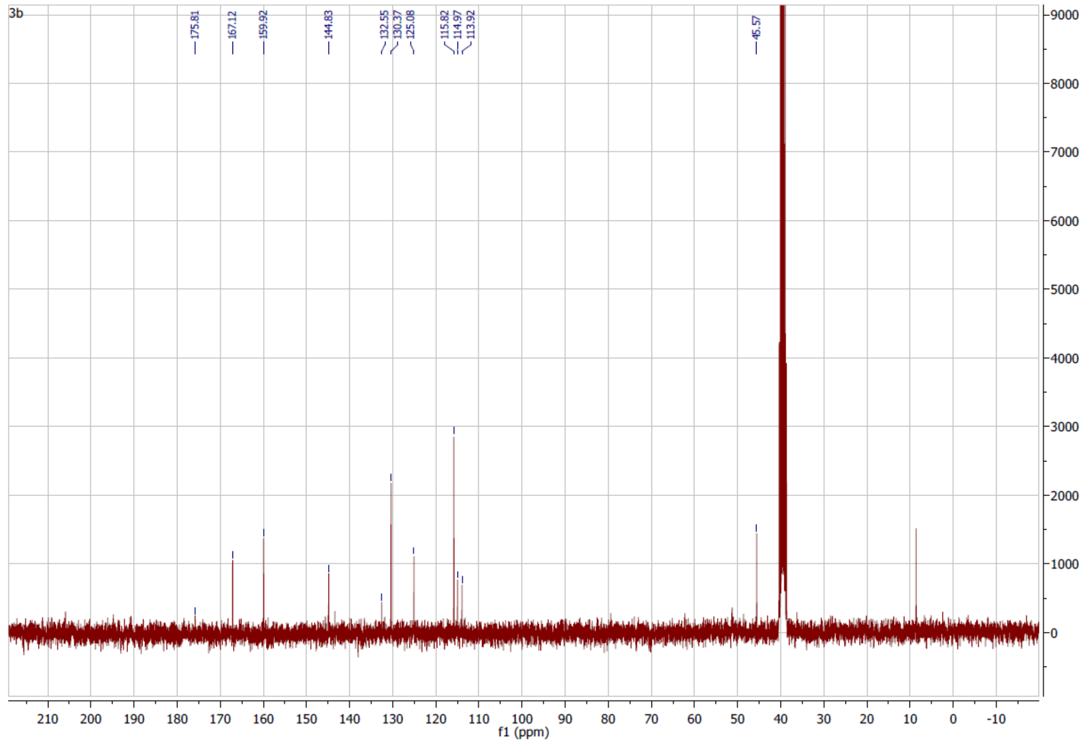

Methyl 2-(3-hydroxyphenyl)acetate **4a**[3]

^{1}H NMR:

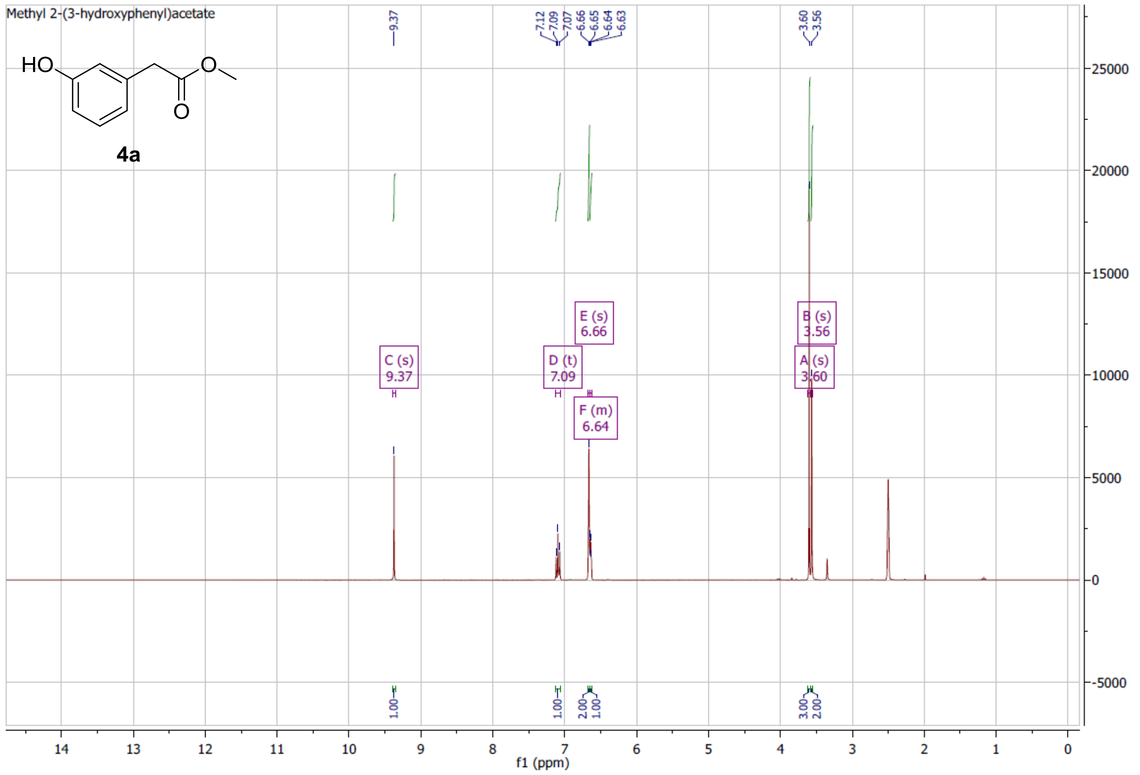

^{13}C NMR

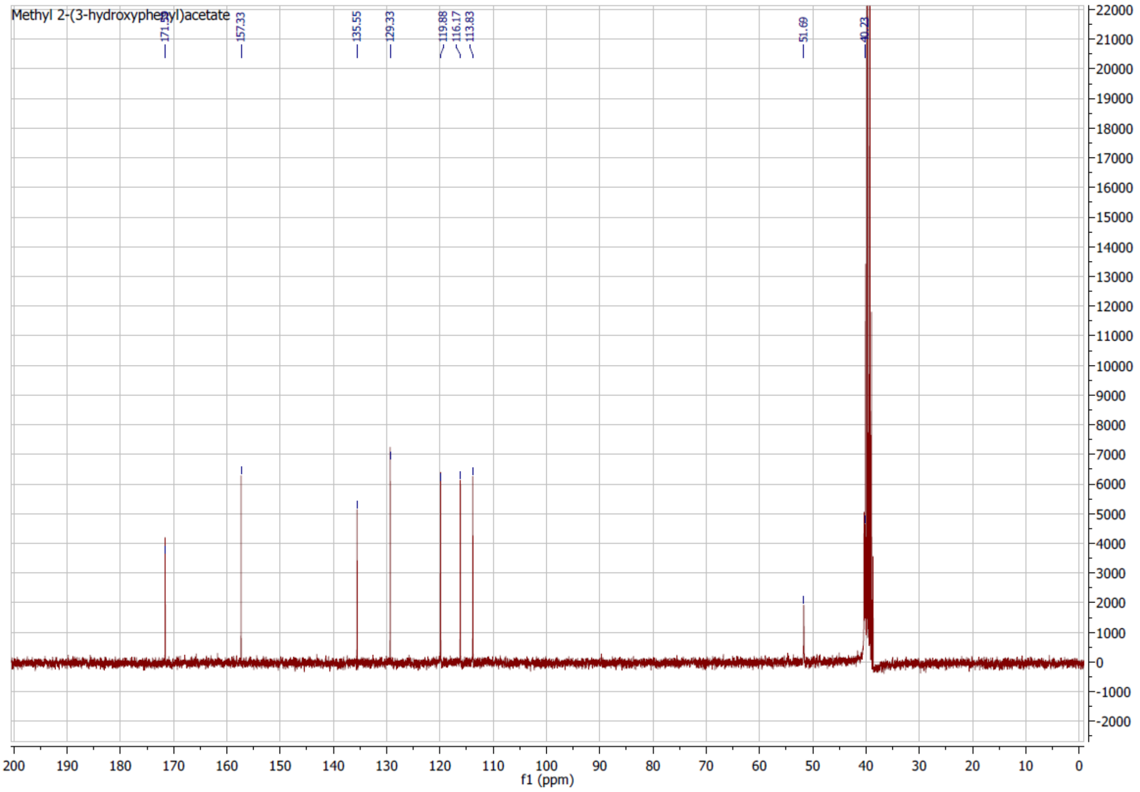

2-Hydroxy-6-(2-methoxy-2-oxoethyl)benzoic acid **4b**[11]

^{1}H NMR:

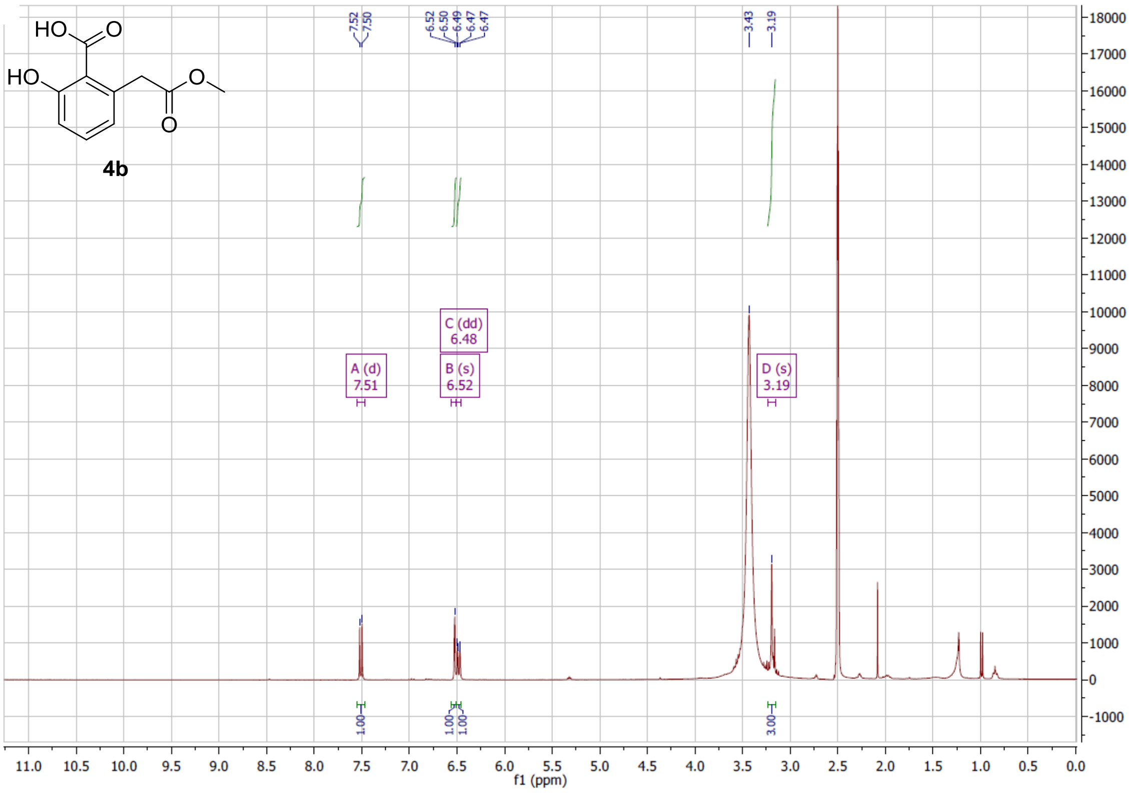

^{13}C NMR:

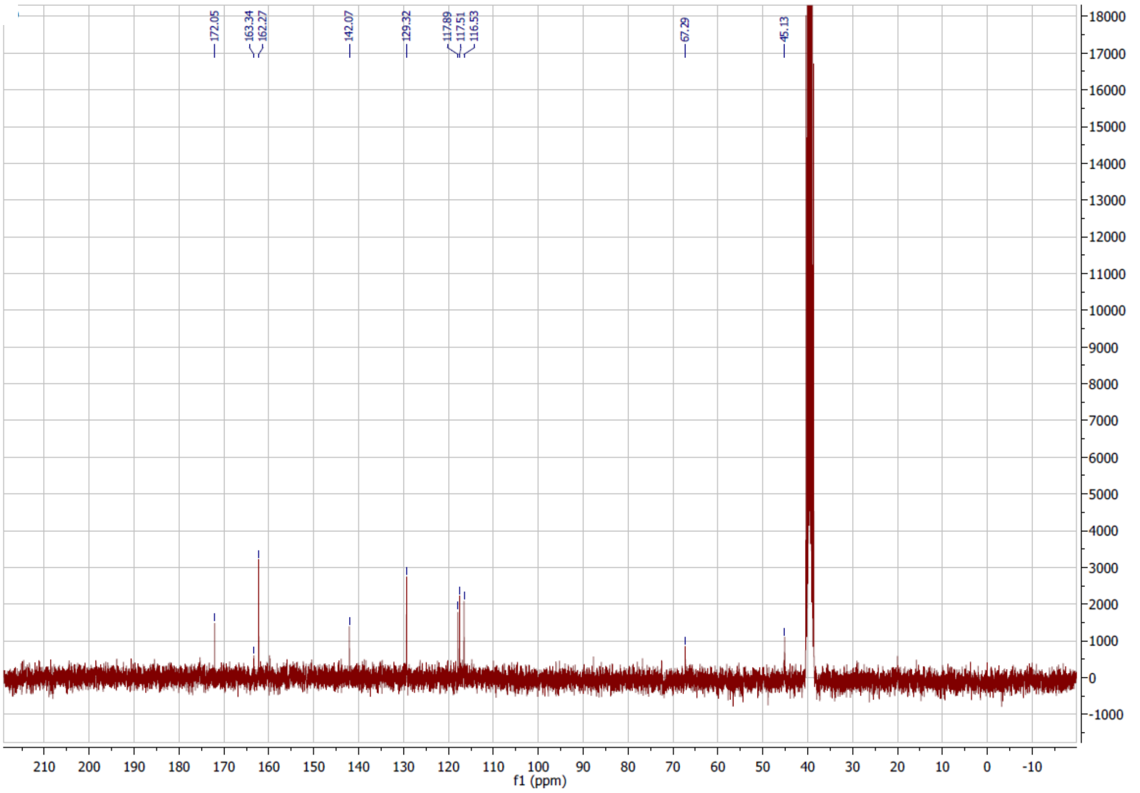

(*E*)-3-(3-Hydroxyphenyl)-1-phenylprop-2-en-1-one **5**

^{1}H NMR:

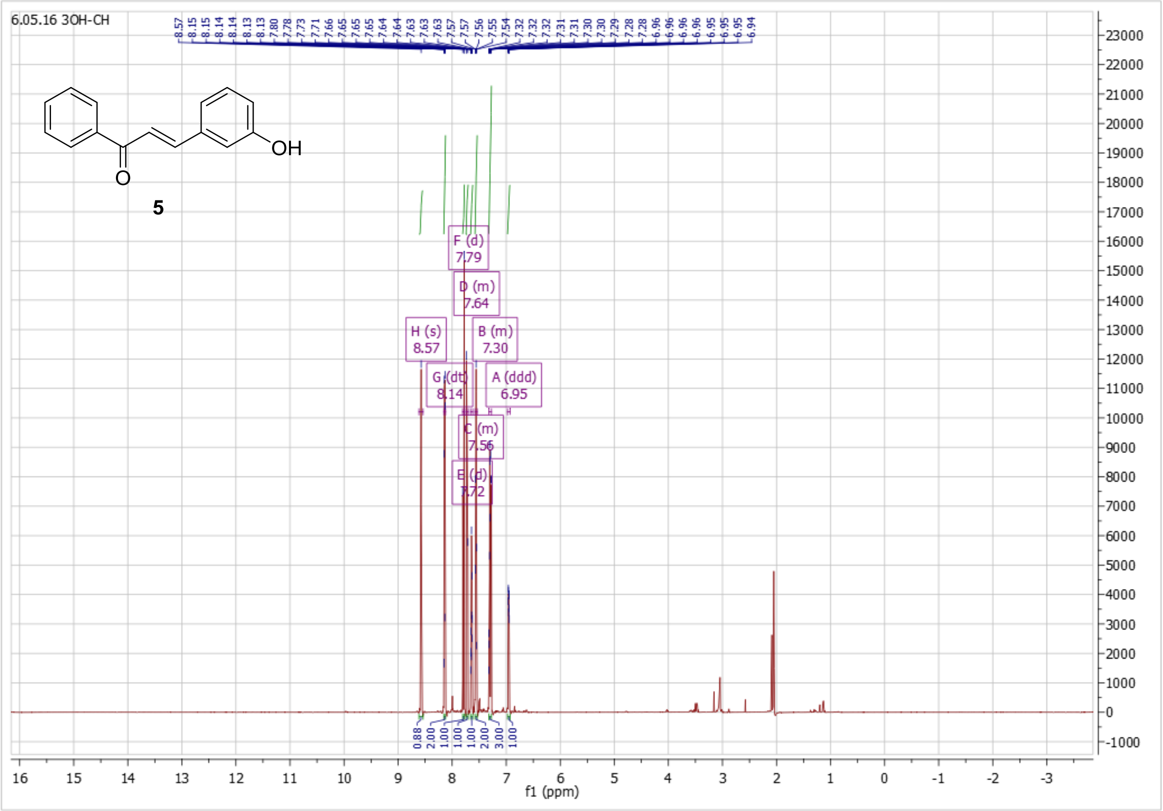

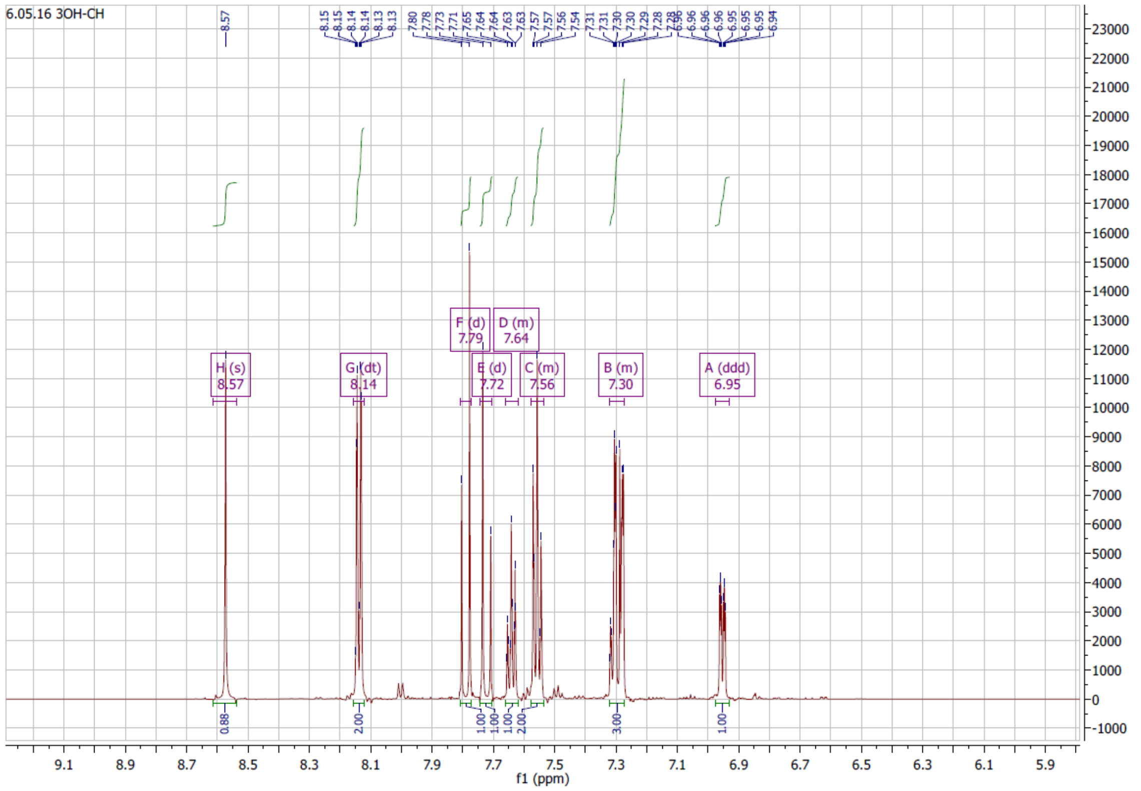

^{13}C NMR:

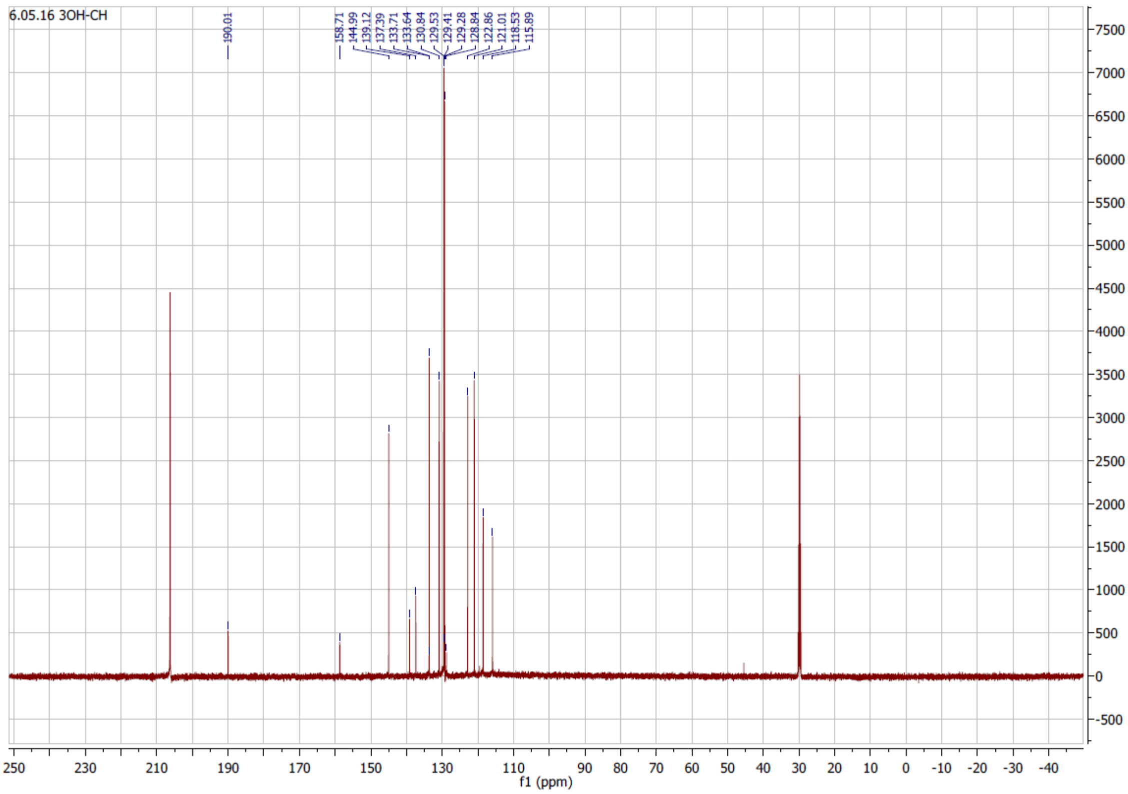

6.05.16 3OH-CH
190.01
158.71
144.99
139.12
137.39
133.71
133.64
130.84
129.53
129.41
129.28
128.84
122.86
121.01
118.53
115.89
f1 (ppm)

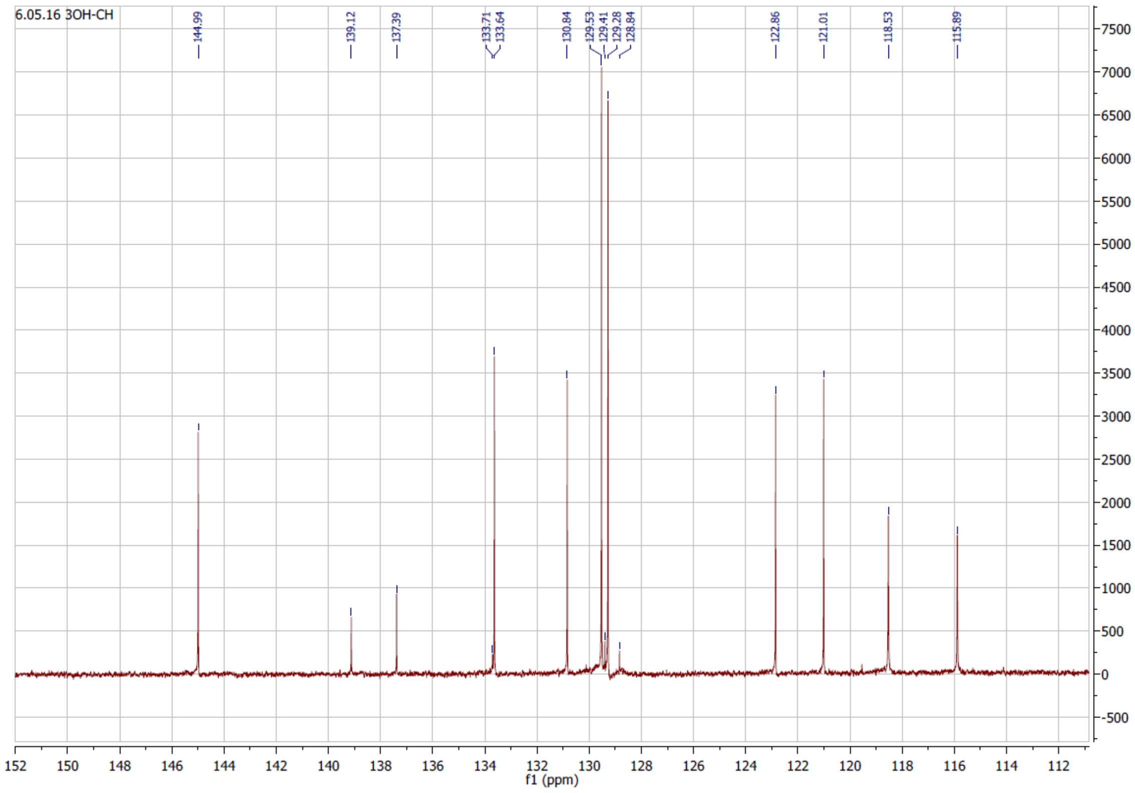

6.05.16 3OH-CH
144.99
139.12
137.39
133.71
133.64
130.84
129.53
129.41
129.28
128.84
122.86
121.01
118.53
115.89
f1 (ppm)

3-(3-Hydroxyphenyl)-1-phenylpropan-1-one **5a**

^{1}H NMR:

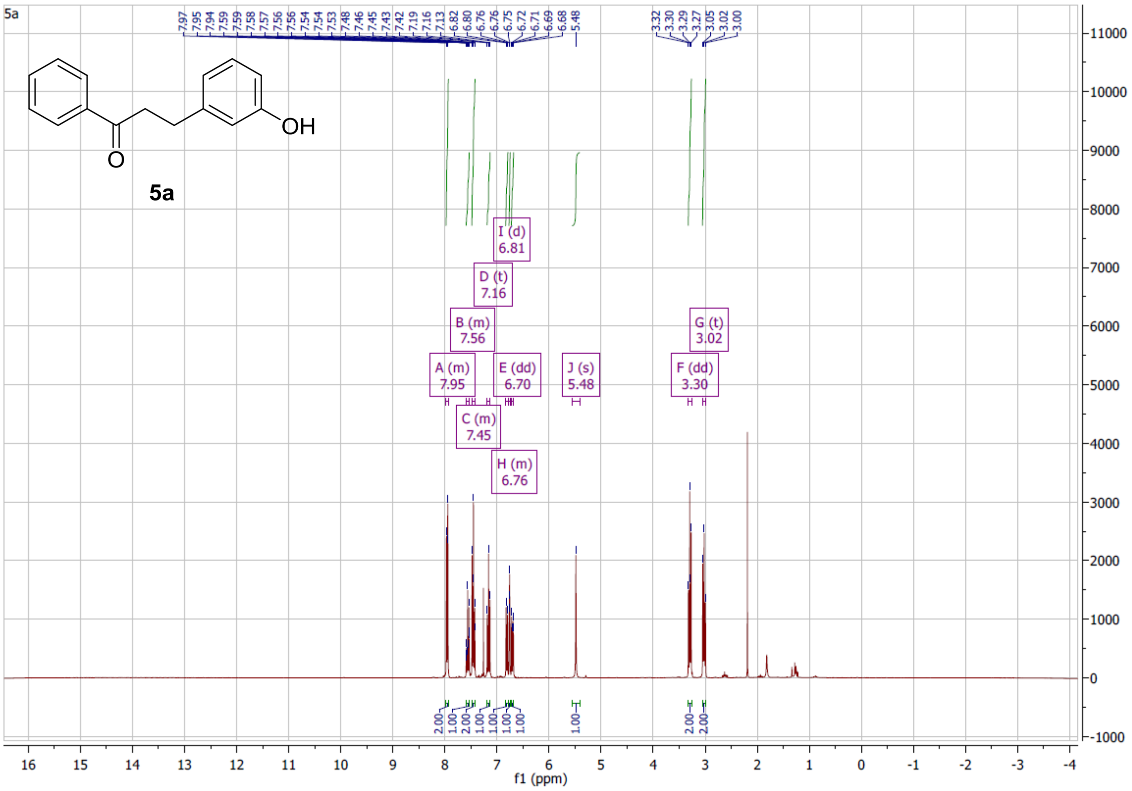

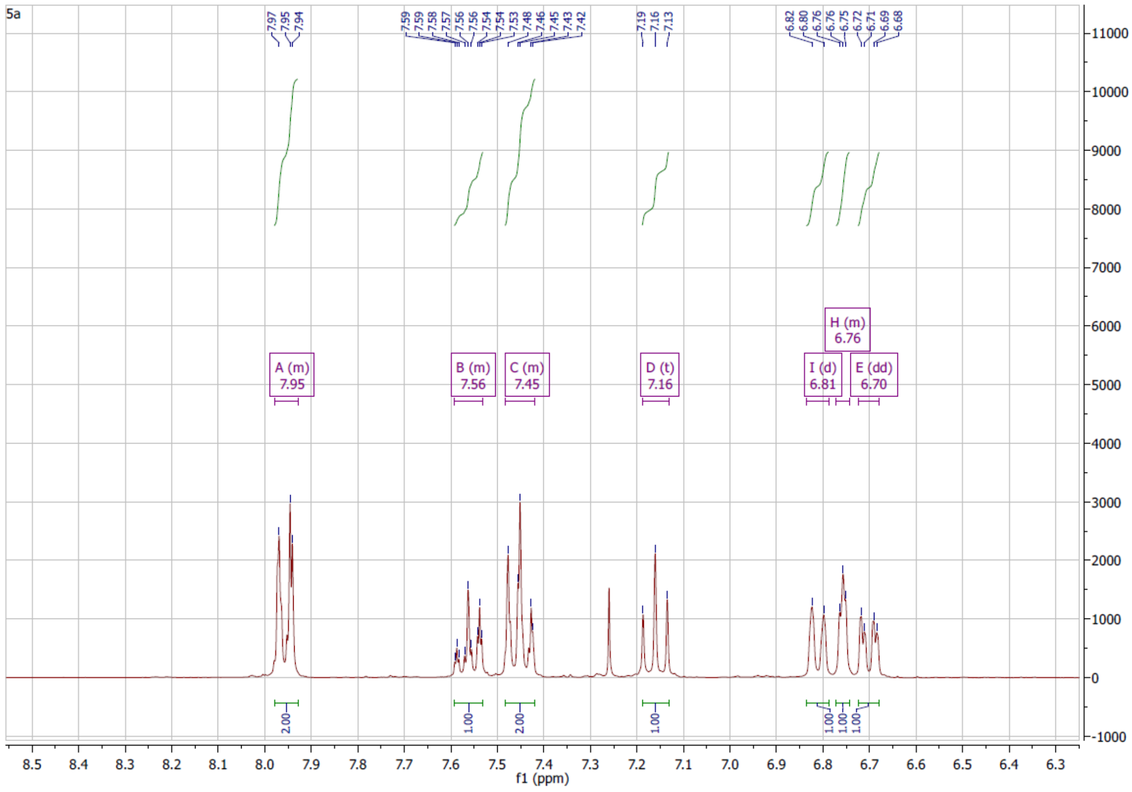

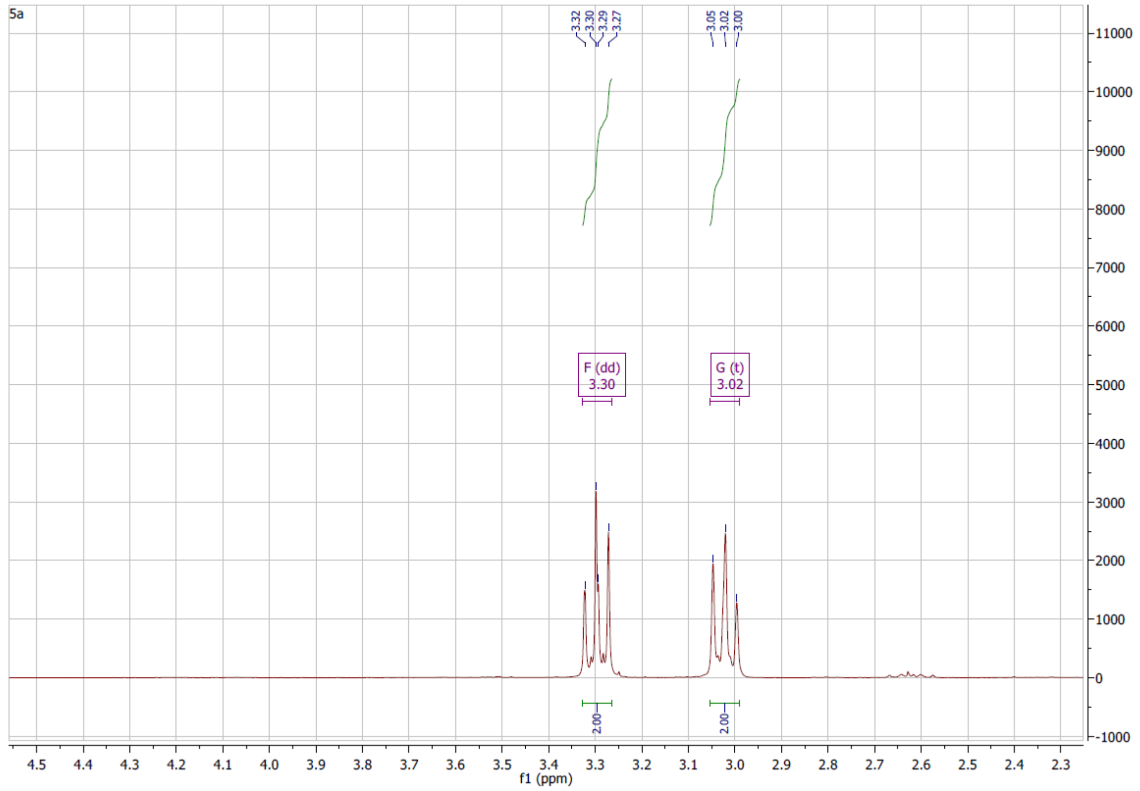
5a
3.32
3.30
3.29
3.27
3.05
3.02
3.00
F (dd)
3.30
G (t)
3.02
2.00
2.00
f1 (ppm)

^{13}C NMR:

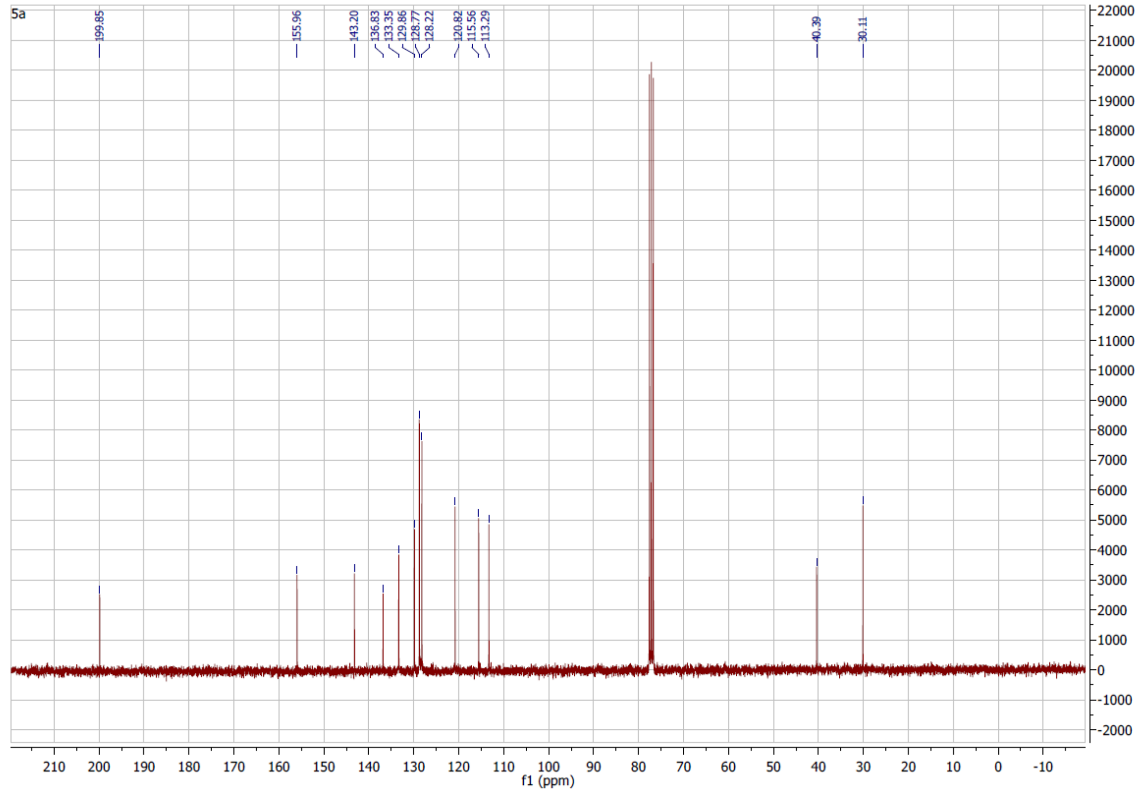
5a
199.85
155.96
143.20
136.83
133.35
129.86
128.77
128.22
120.82
115.56
113.29
40.39
30.11
f1 (ppm)

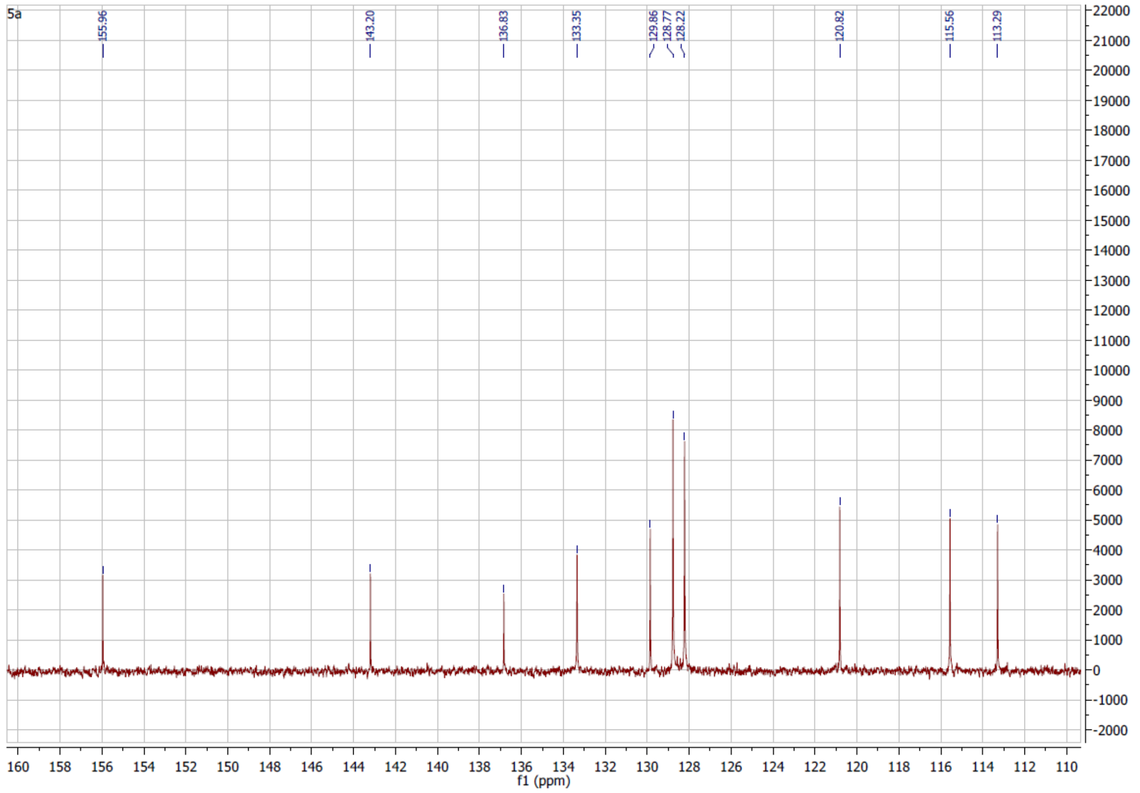
5a
155.96
143.20
136.83
133.35
129.86
128.77
128.22
120.82
115.56
113.29
f1 (ppm)

COSY:

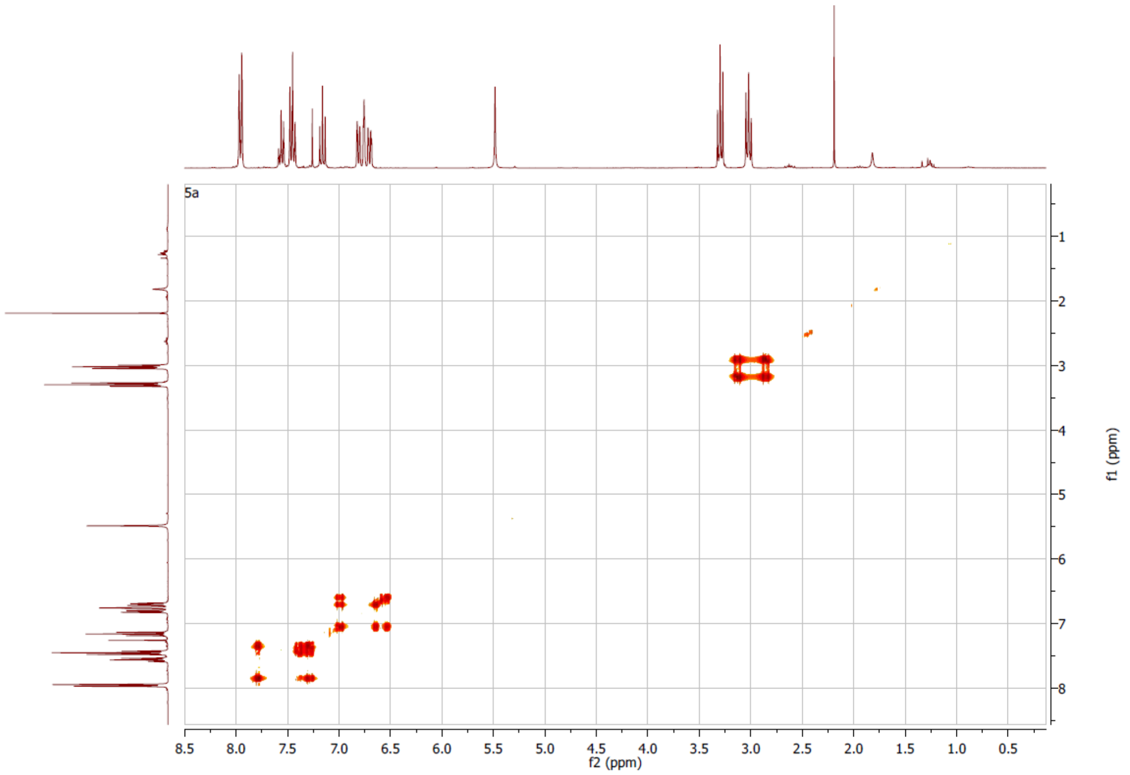
5a
f2 (ppm)
f1 (ppm)

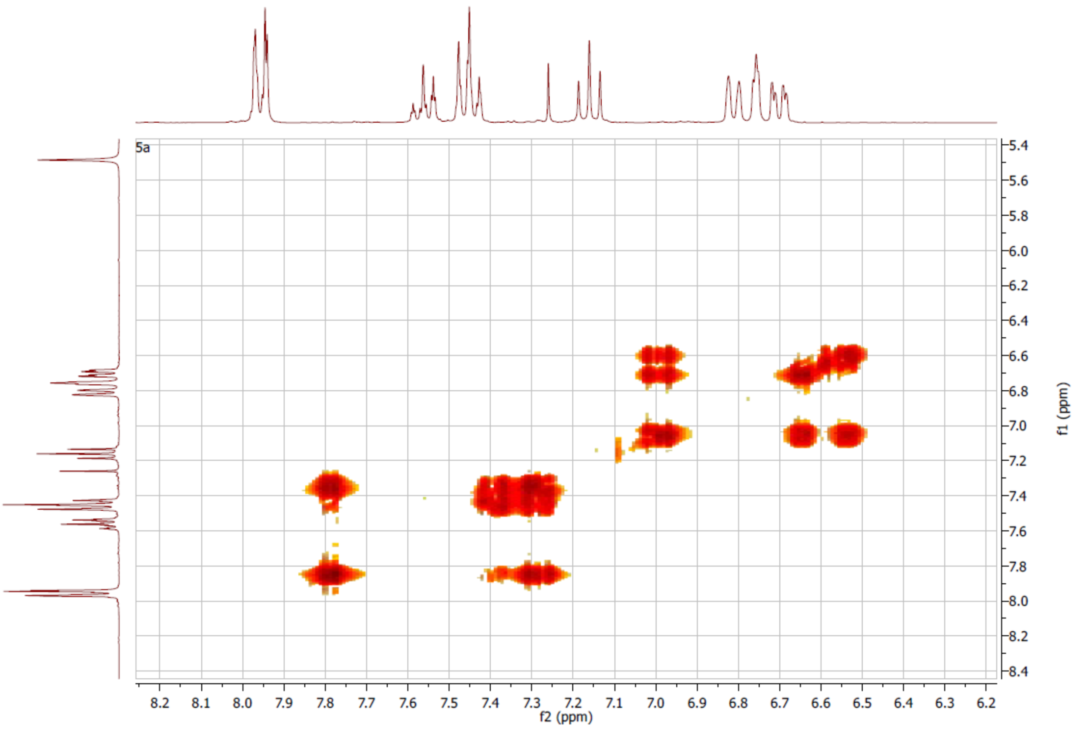
5a
f2 (ppm)
f1 (ppm)

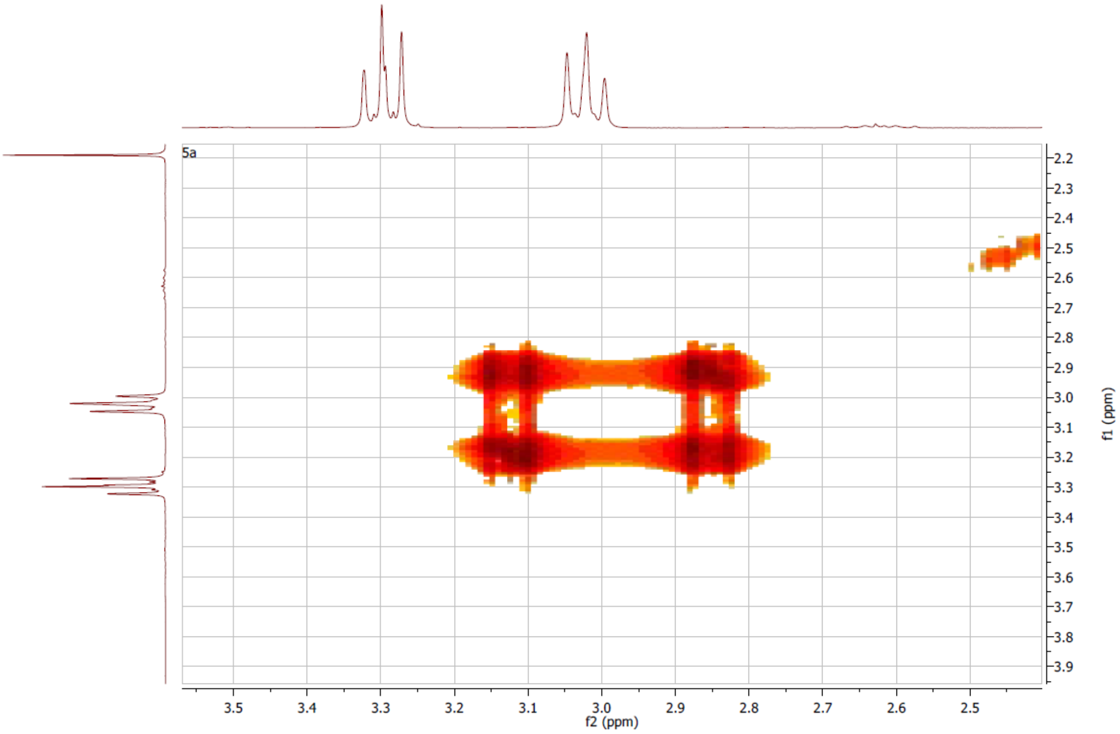
5a
f2 (ppm)
f1 (ppm)

HMBC:

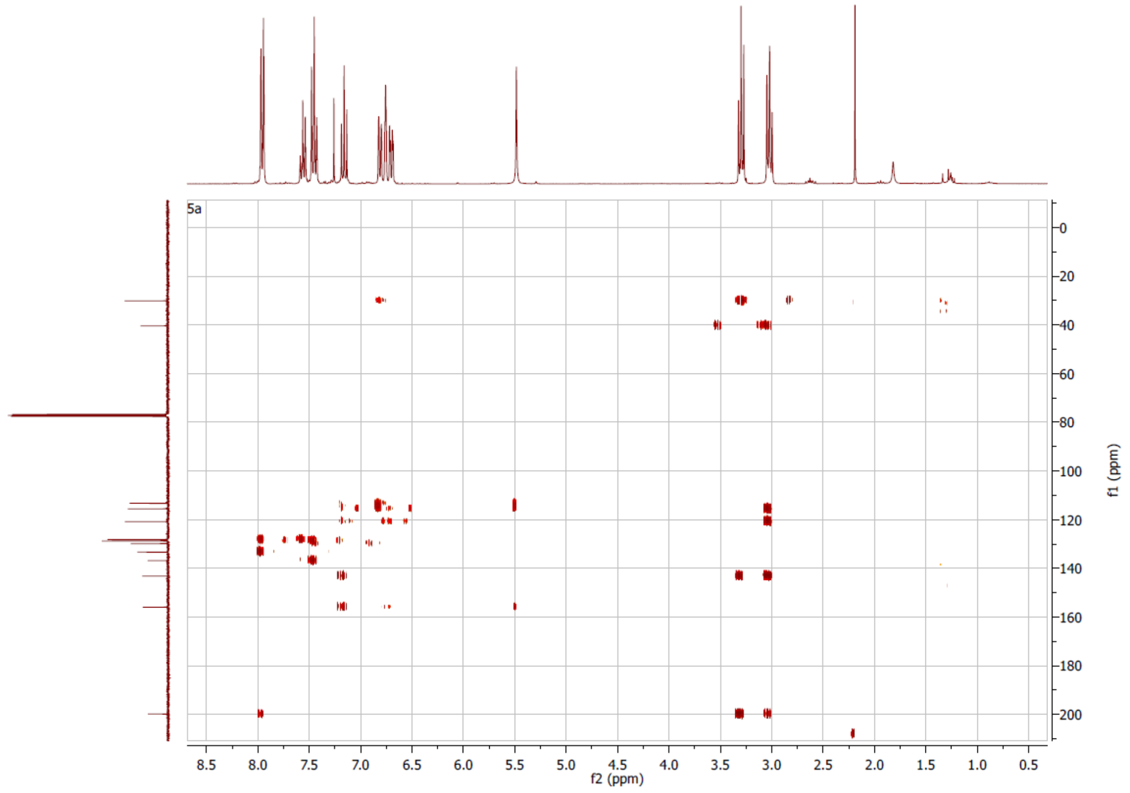

HSQC:

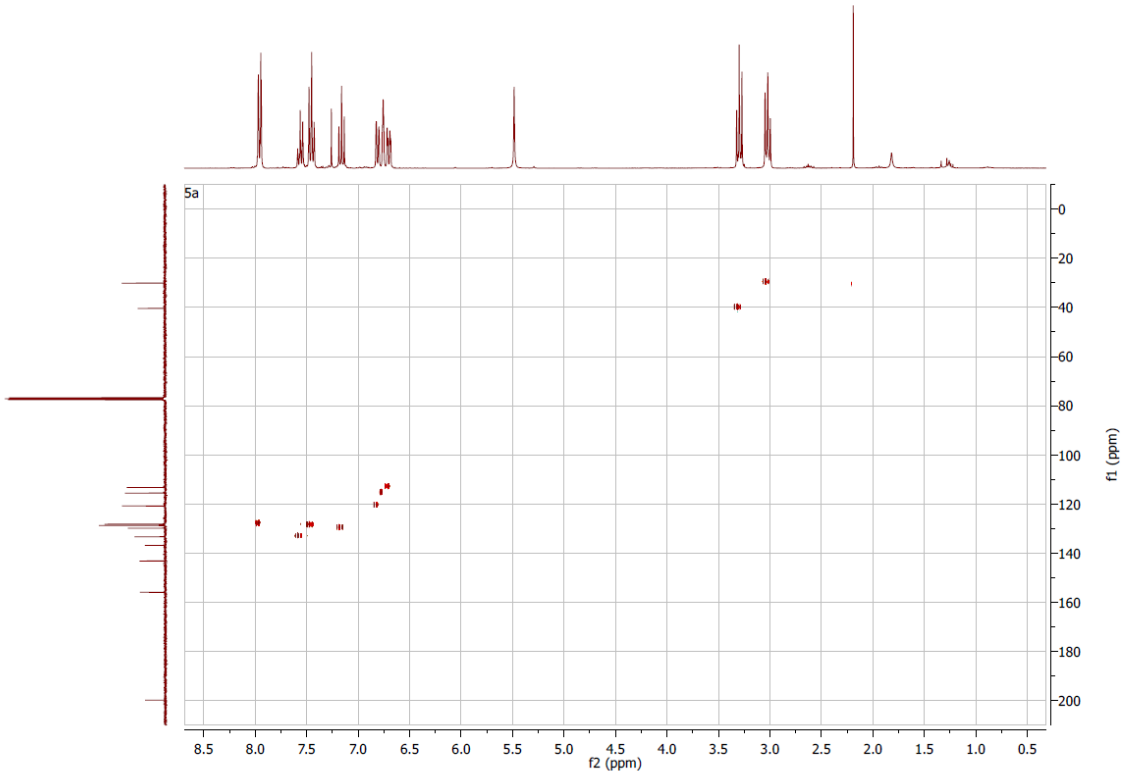

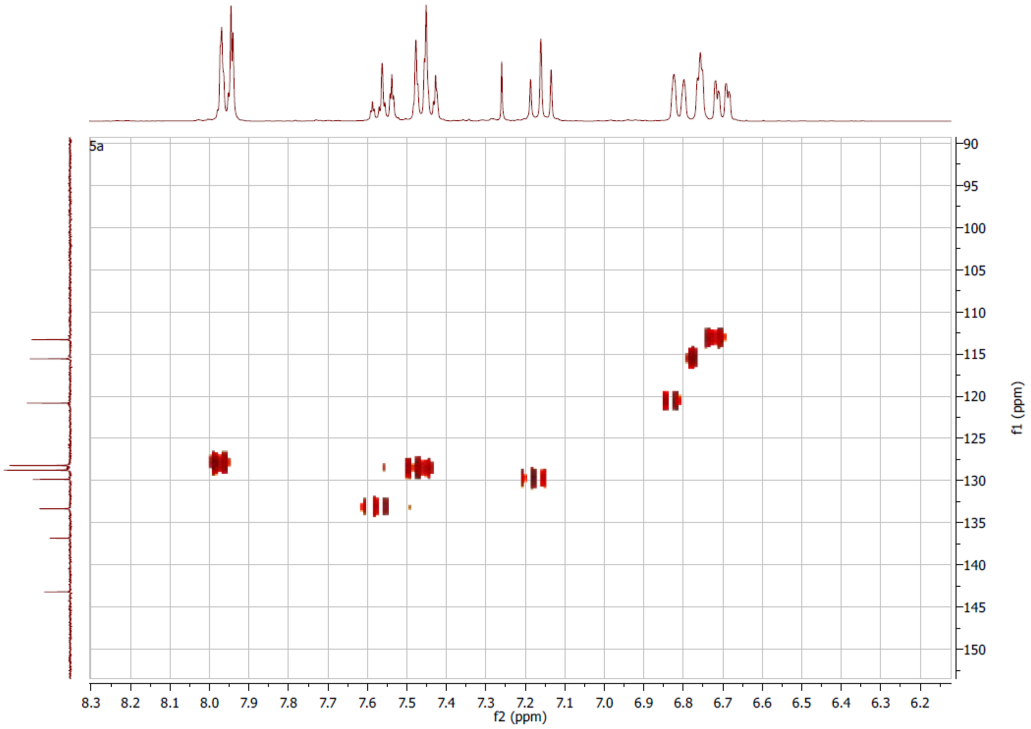
5a
f2 (ppm)
f1 (ppm)

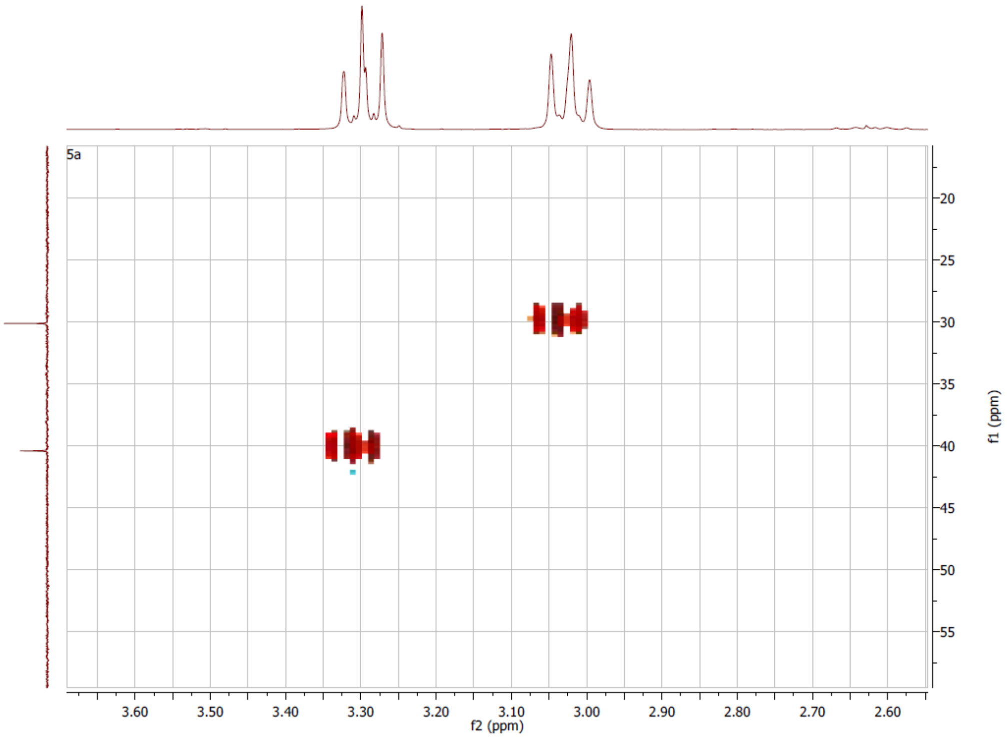
5a
f2 (ppm)
f1 (ppm)

2-Hydroxy-4-(3-oxo-3-phenylpropyl)benzoic acid **5b**

^{1}H NMR:

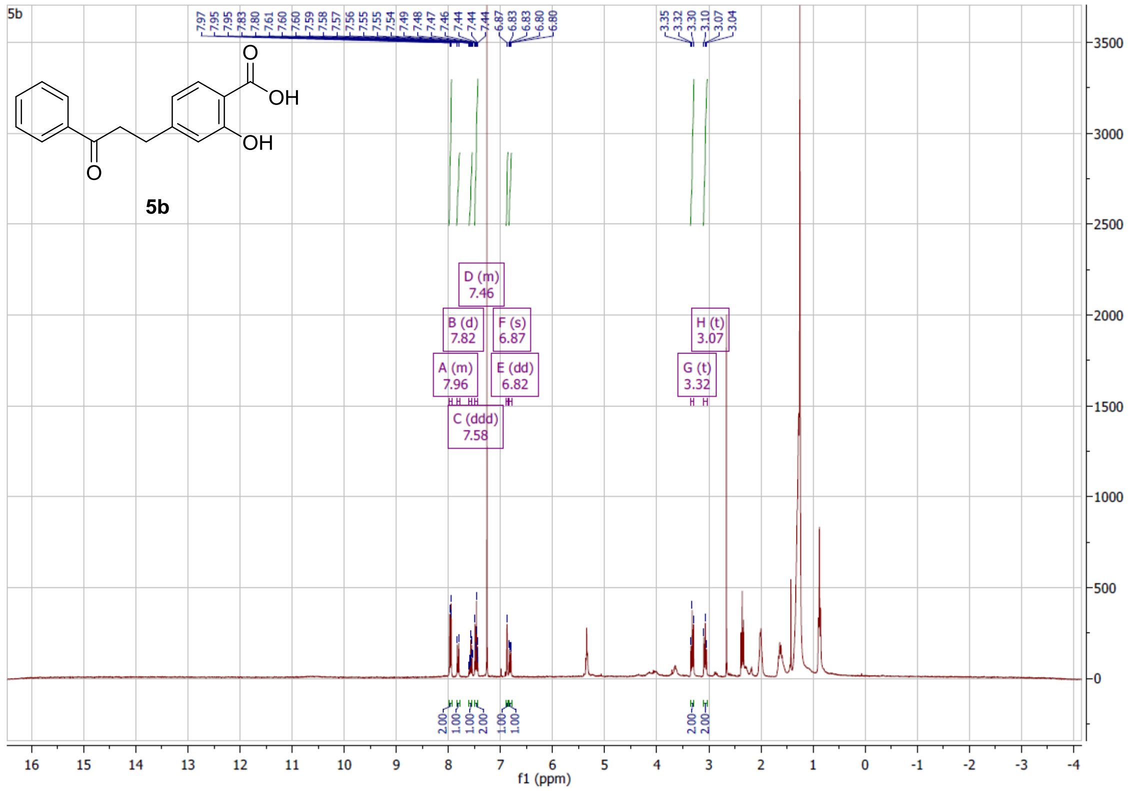

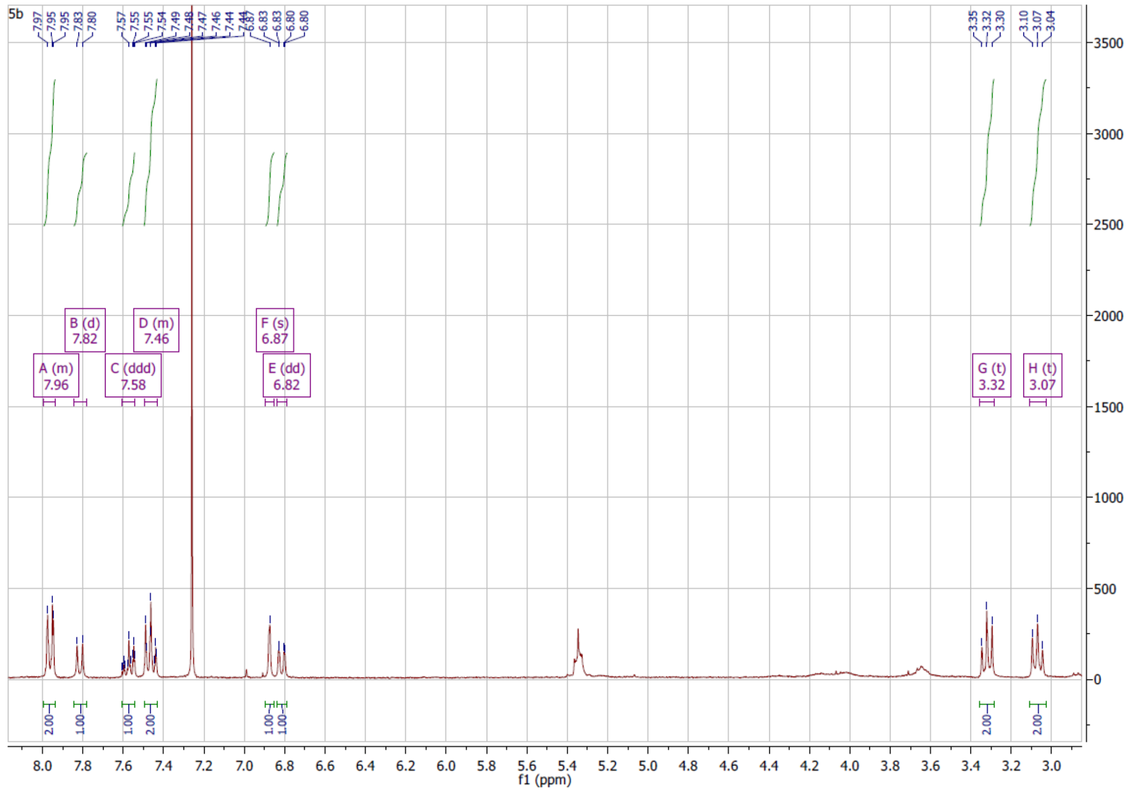

^{13}C NMR:

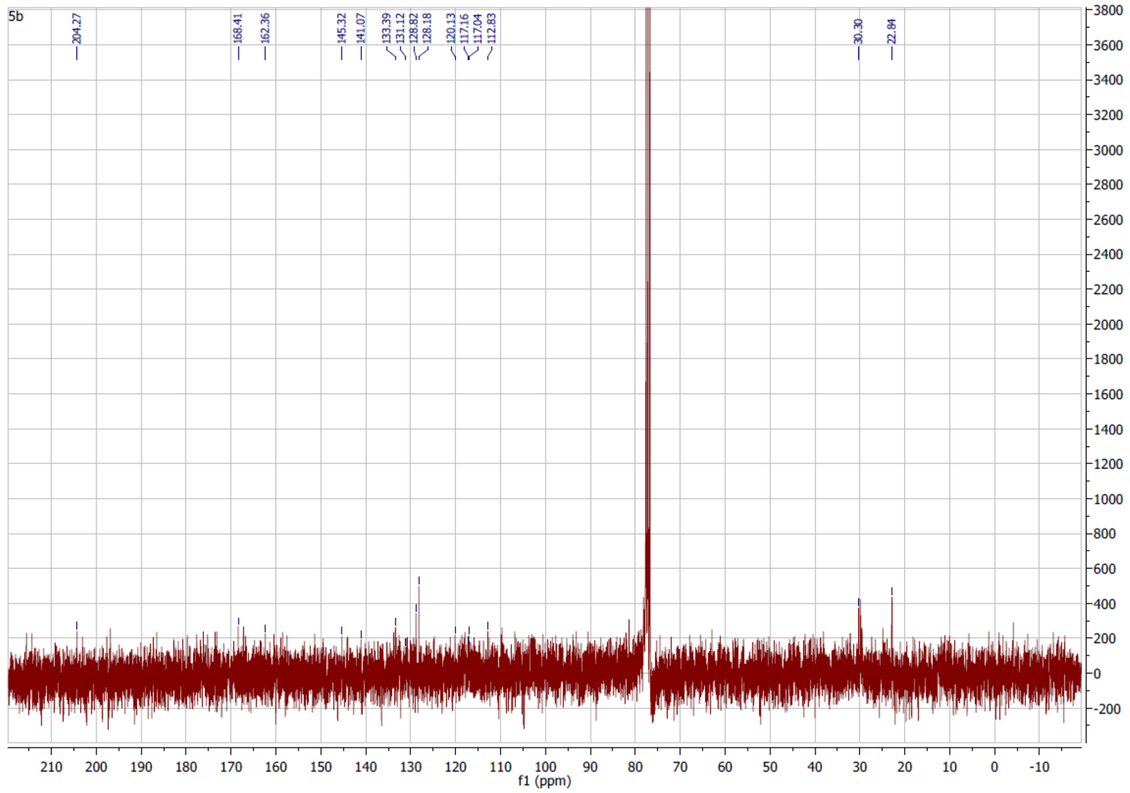

5b
204.27
168.41
162.36
145.32
141.07
133.39
131.12
128.82
128.18
120.13
117.16
117.04
112.83
30.30
22.84
f1 (ppm)

COSY:

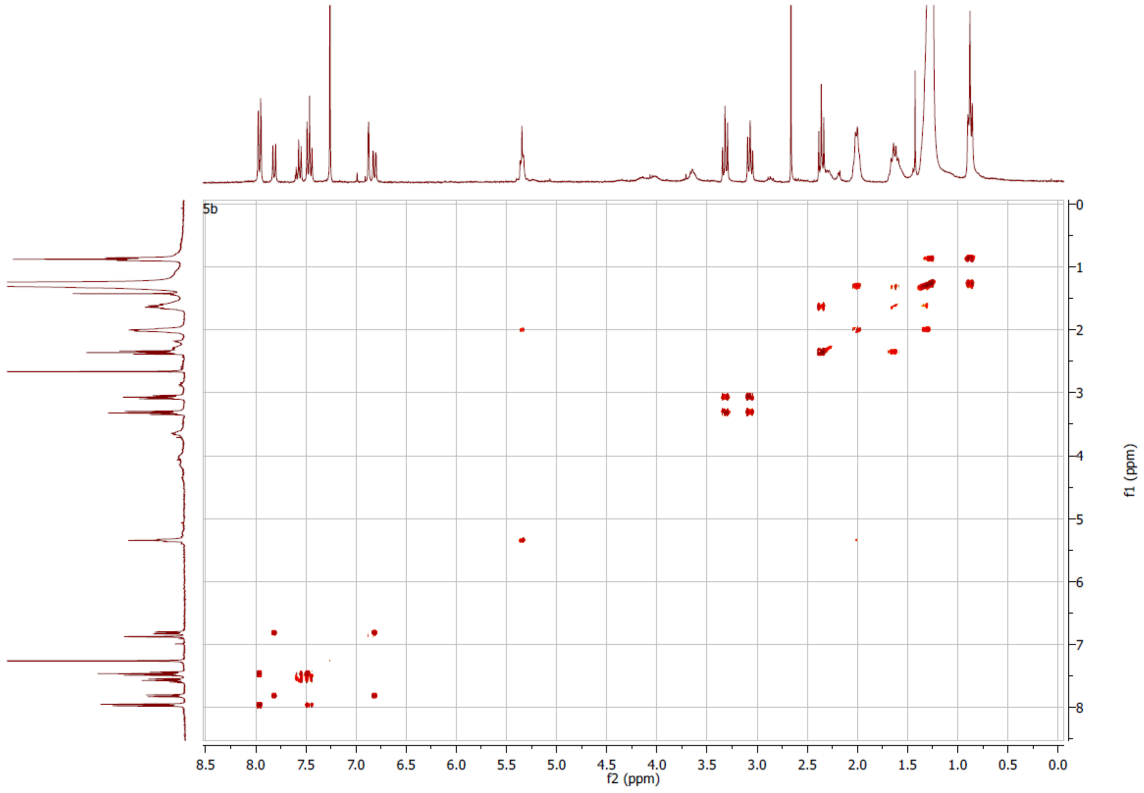

5b
f2 (ppm)
f1 (ppm)

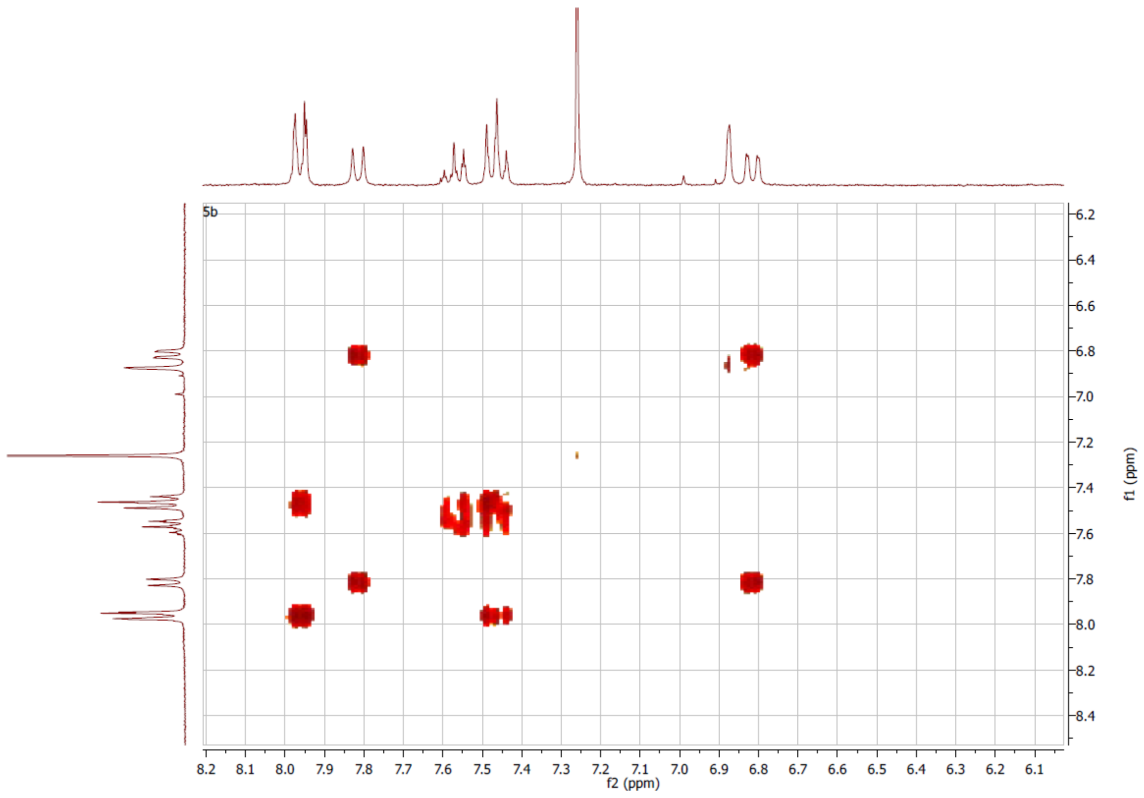
5b
6.2
6.4
6.6
6.8
7.0
7.2
7.4
7.6
7.8
8.0
8.2
8.4
f1 (ppm)
8.2 8.1 8.0 7.9 7.8 7.7 7.6 7.5 7.4 7.3 7.2 7.1 7.0 6.9 6.8 6.7 6.6 6.5 6.4 6.3 6.2 6.1
f2 (ppm)

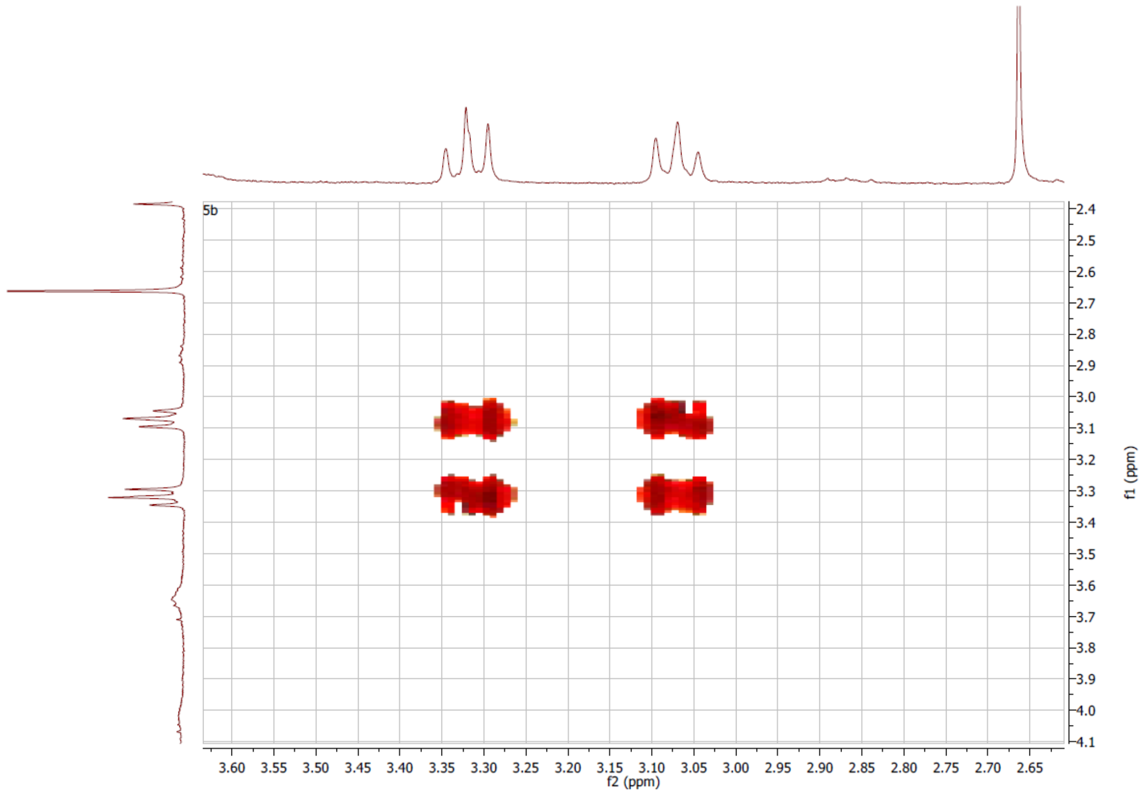
5b
2.4
2.5
2.6
2.7
2.8
2.9
3.0
3.1
3.2
3.3
3.4
3.5
3.6
3.7
3.8
3.9
4.0
4.1
f1 (ppm)
3.60 3.55 3.50 3.45 3.40 3.35 3.30 3.25 3.20 3.15 3.10 3.05 3.00 2.95 2.90 2.85 2.80 2.75 2.70 2.65
f2 (ppm)

HSQC:

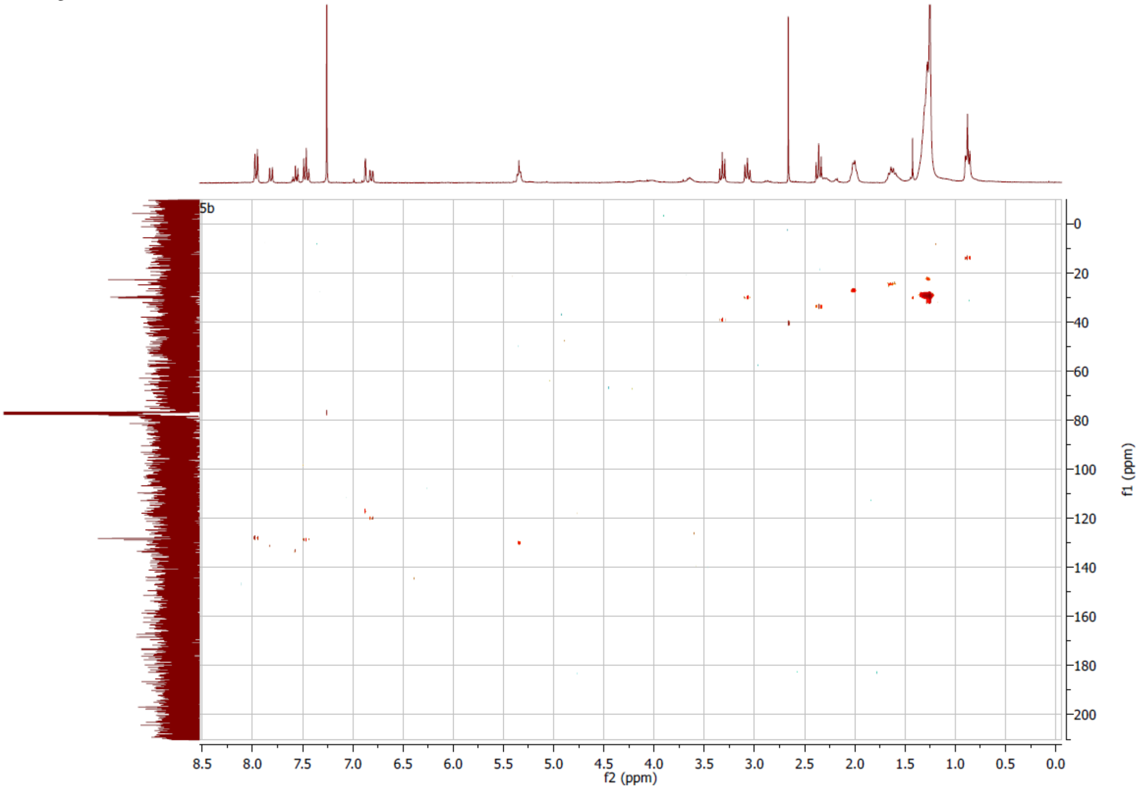

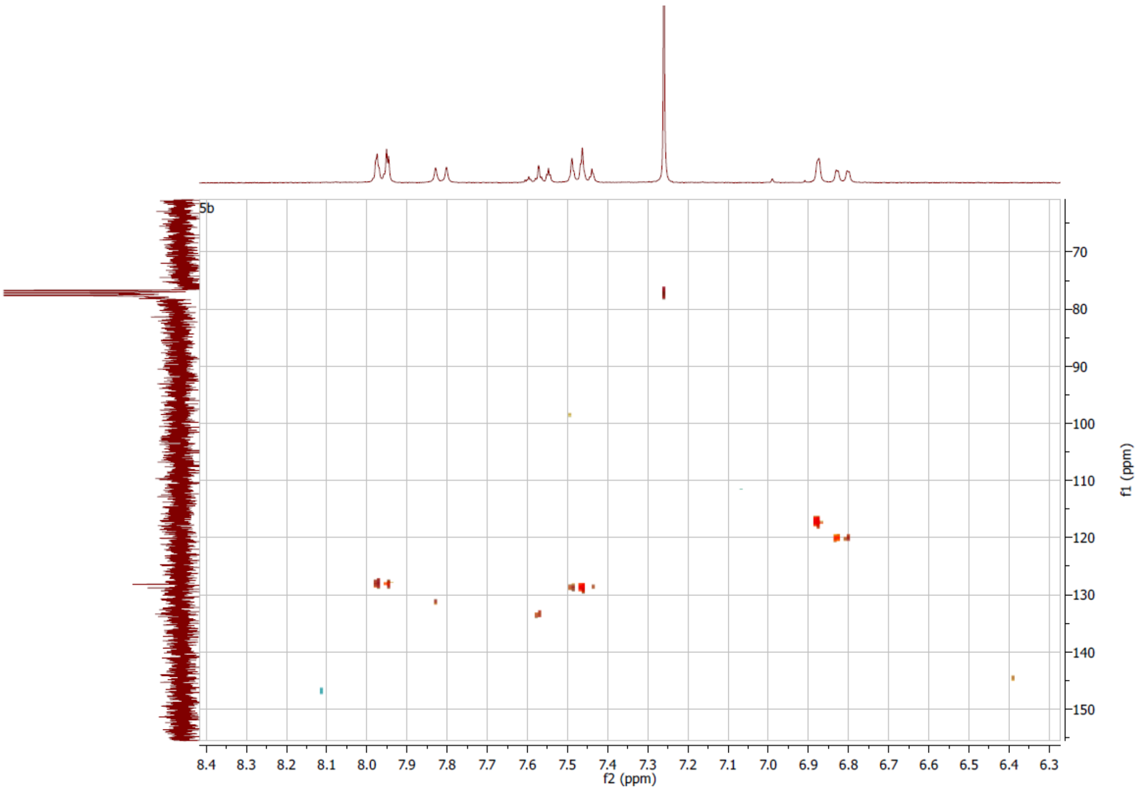

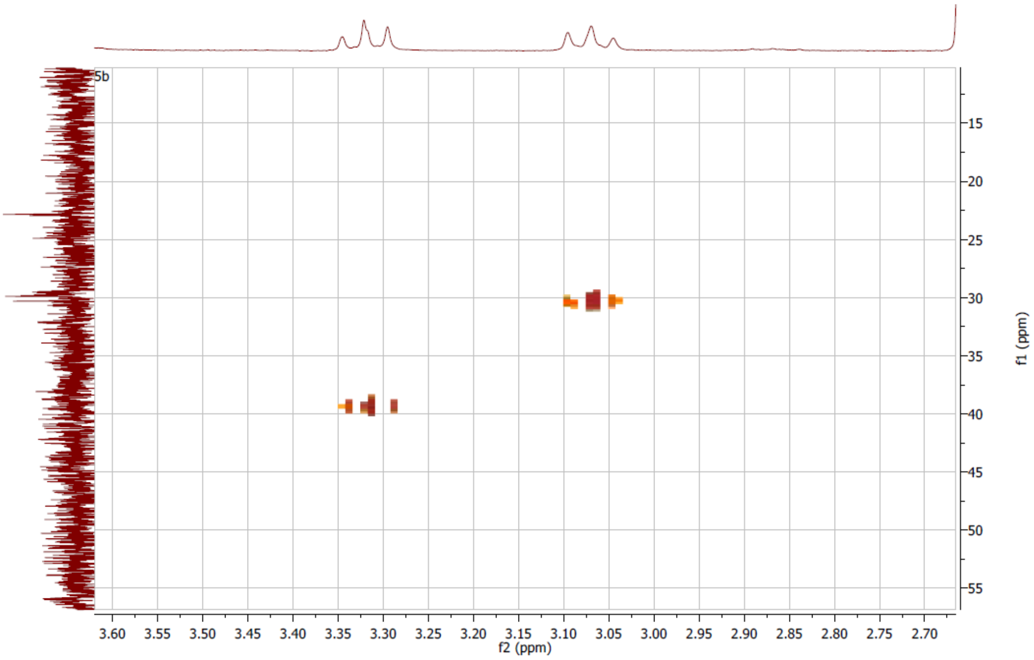

HMBC

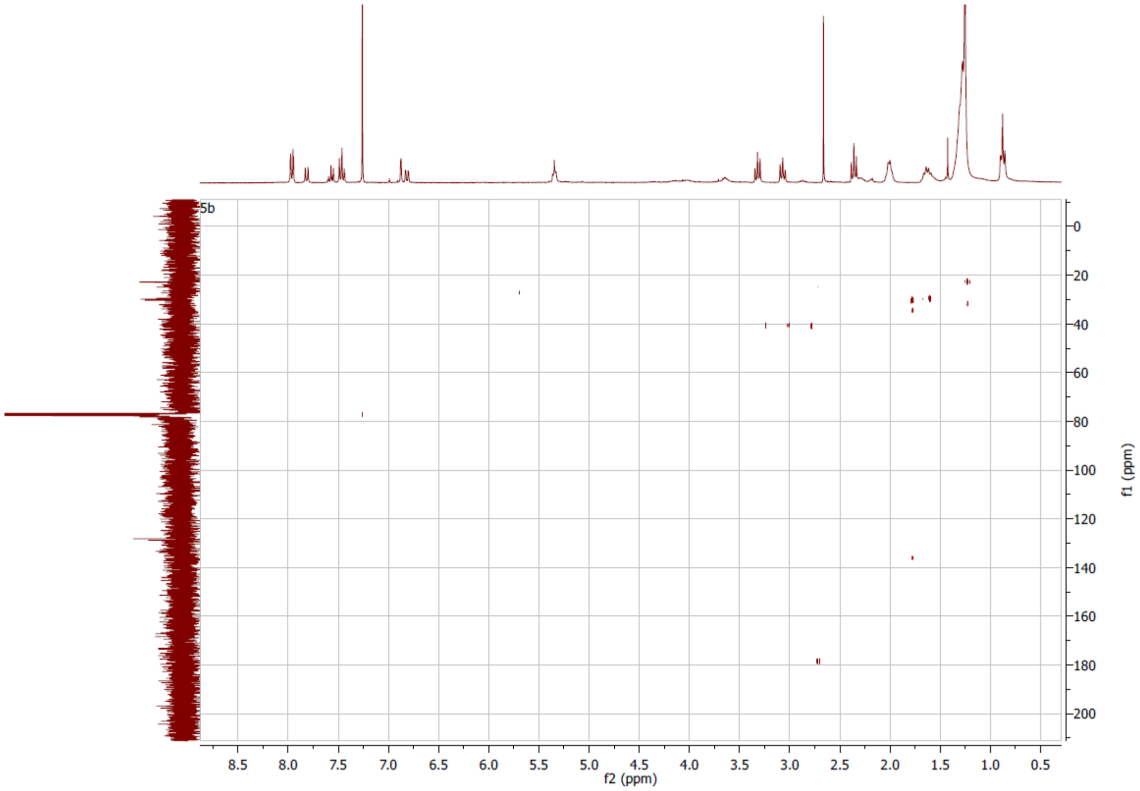

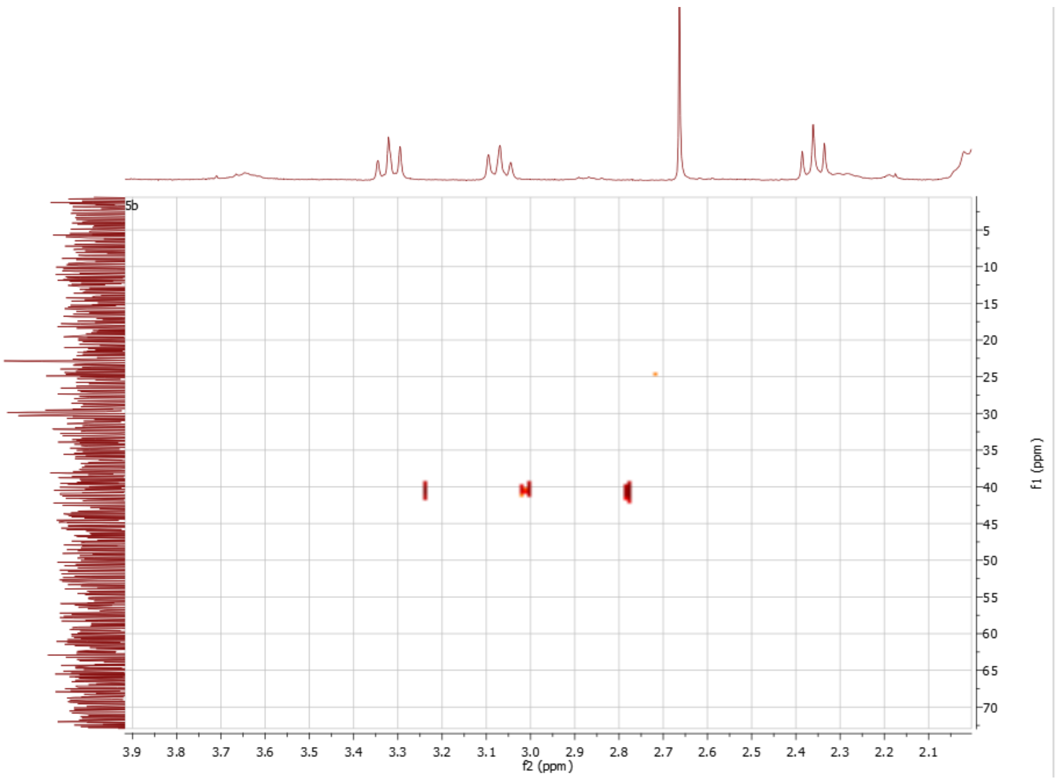

High resolution MS

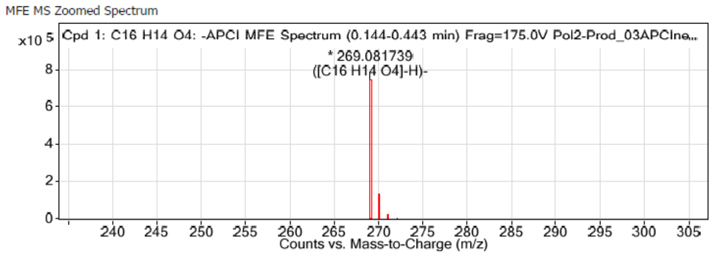

Calculated:	269,081739
Found:	269,081932
Mass accuracity	0,72 ppm

2,4,6-Trihydroxy-3-[3-(4-hydroxyphenyl)propanoyl]benzoic acid **6b**

^{1}H NMR:

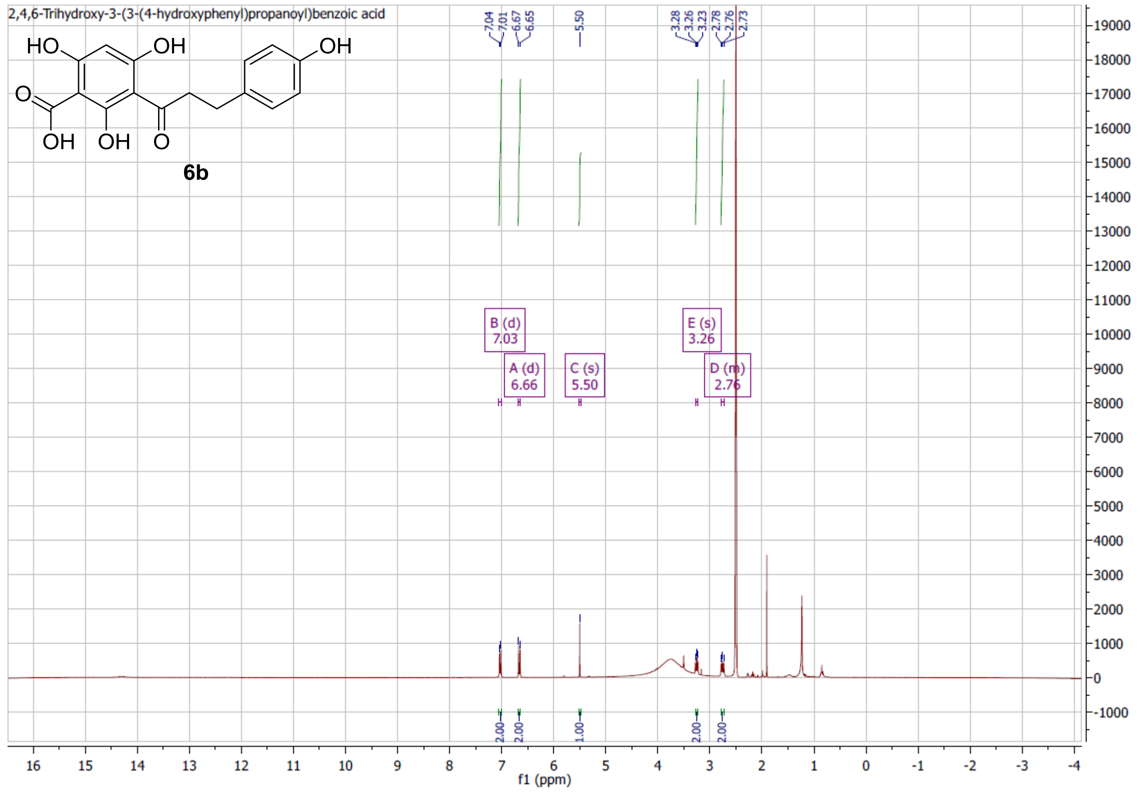

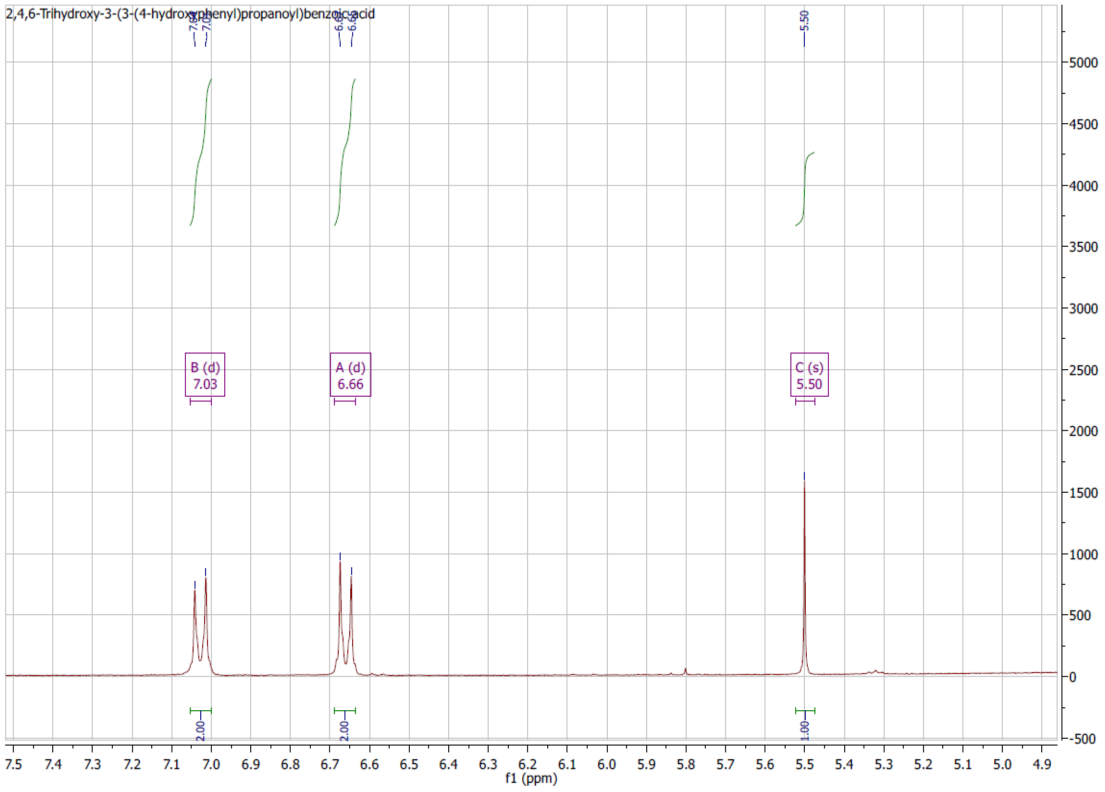

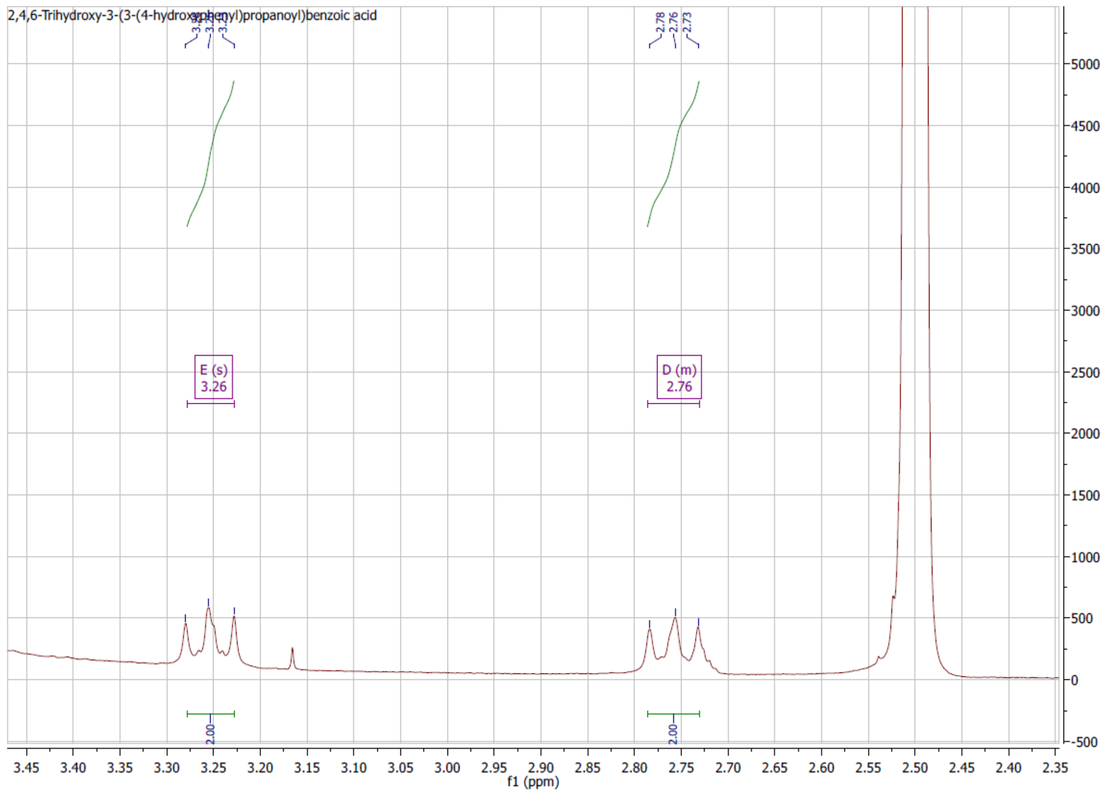
2,4,6-Trihydroxy-3-(3-(4-hydroxyphenyl)propanoyl)benzoic acid
2.78
2.76
2.73
E (s)
3.26
D (m)
2.76
2.00
2.00
f1 (ppm)

^{13}C NMR:

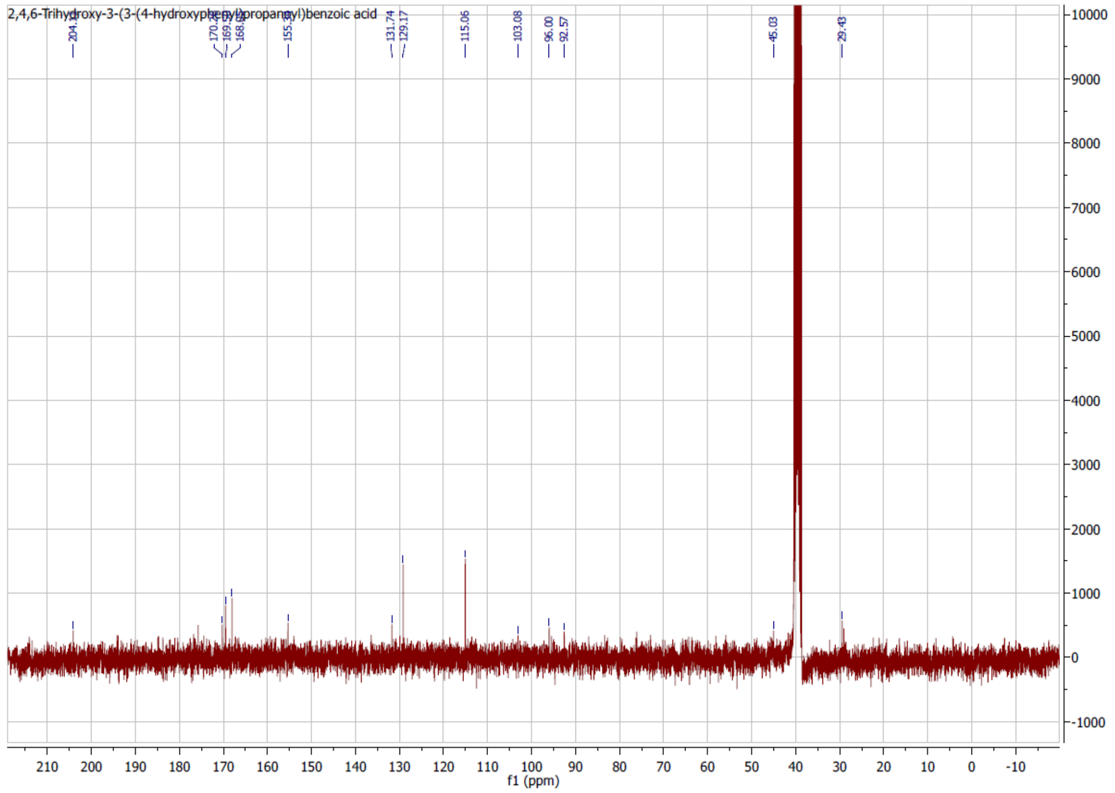
2,4,6-Trihydroxy-3-(3-(4-hydroxyphenyl)propanoyl)benzoic acid
131.74
129.17
115.06
103.08
96.00
92.57
45.03
29.43
f1 (ppm)

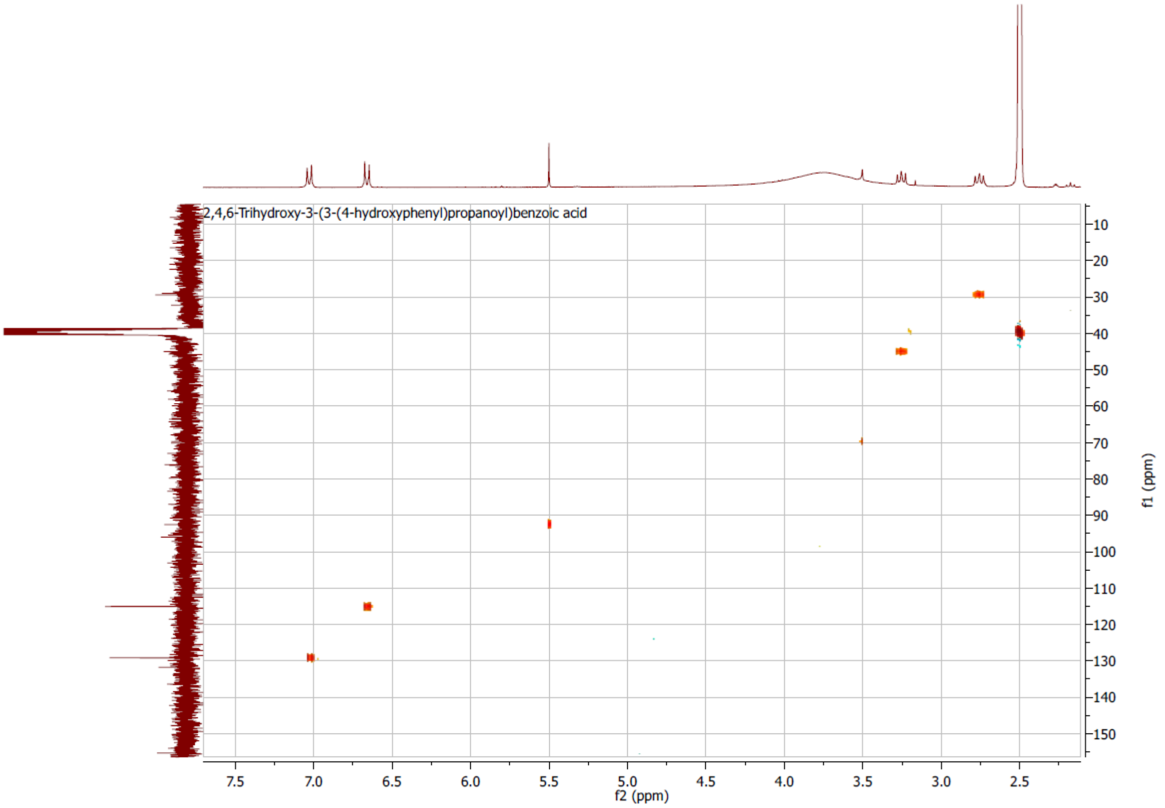
2,4,6-Trihydroxy-3-(3-(4-hydroxyphenyl)propanoyl)benzoic acid
f2 (ppm)
f1 (ppm)

HSQC:

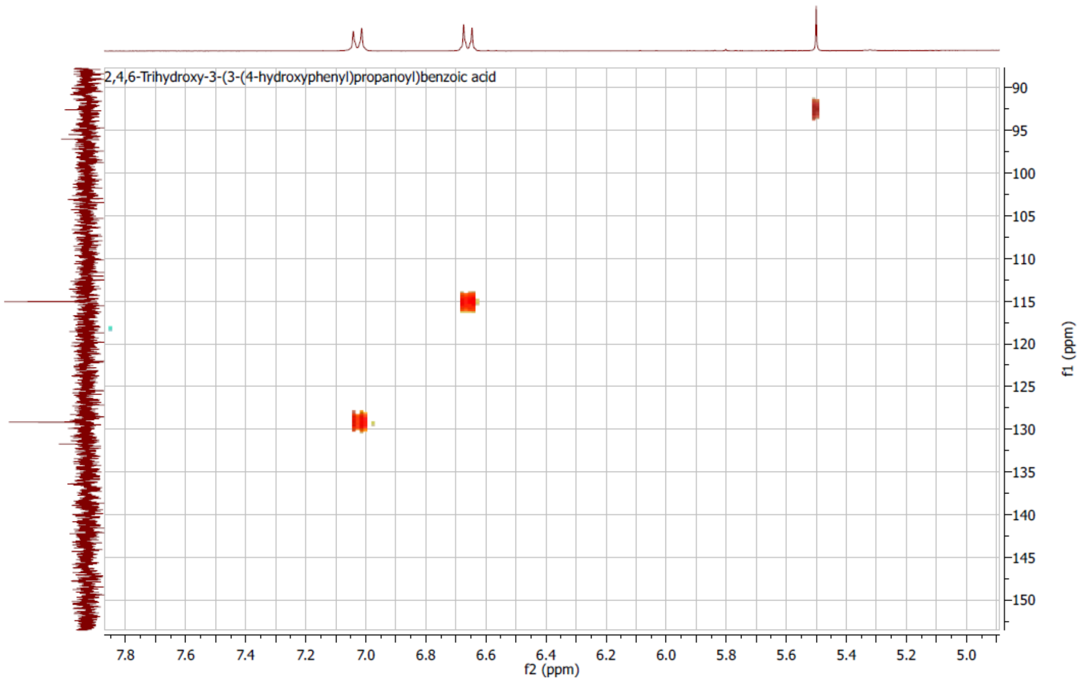
2,4,6-Trihydroxy-3-(3-(4-hydroxyphenyl)propanoyl)benzoic acid
f2 (ppm)
f1 (ppm)

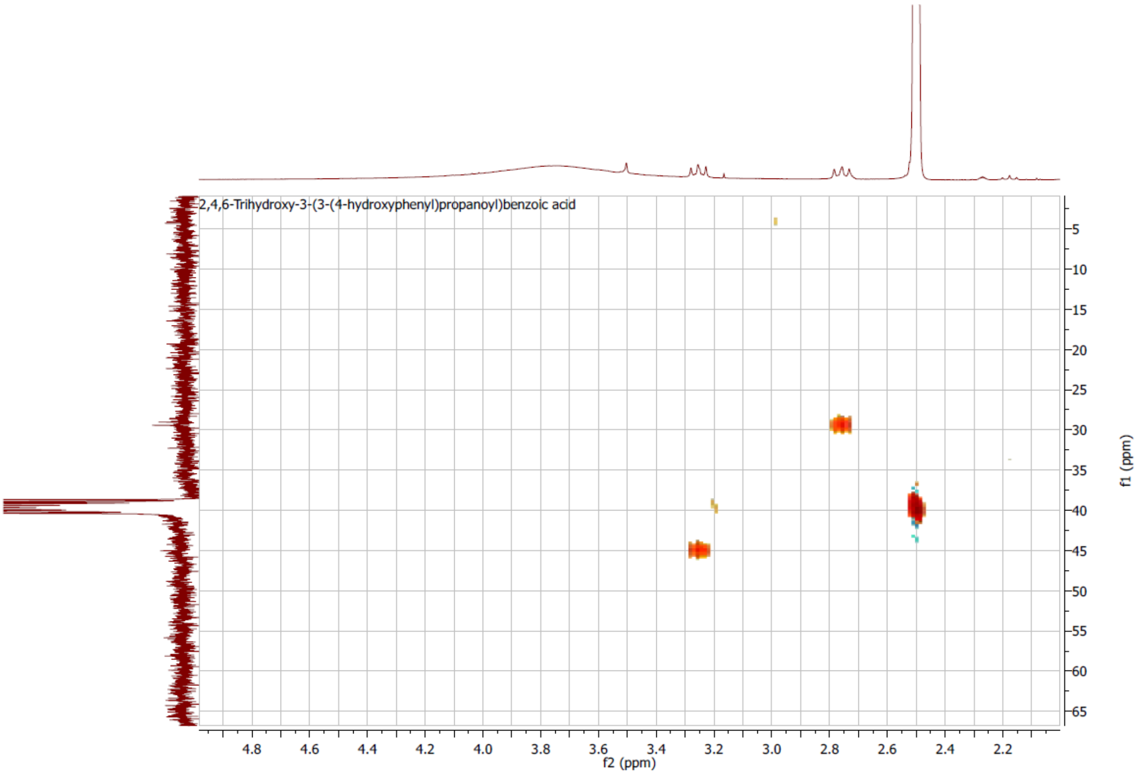
2,4,6-Trihydroxy-3-(3-(4-hydroxyphenyl)propanoyl)benzoic acid
f2 (ppm)
f1 (ppm)

COSY:

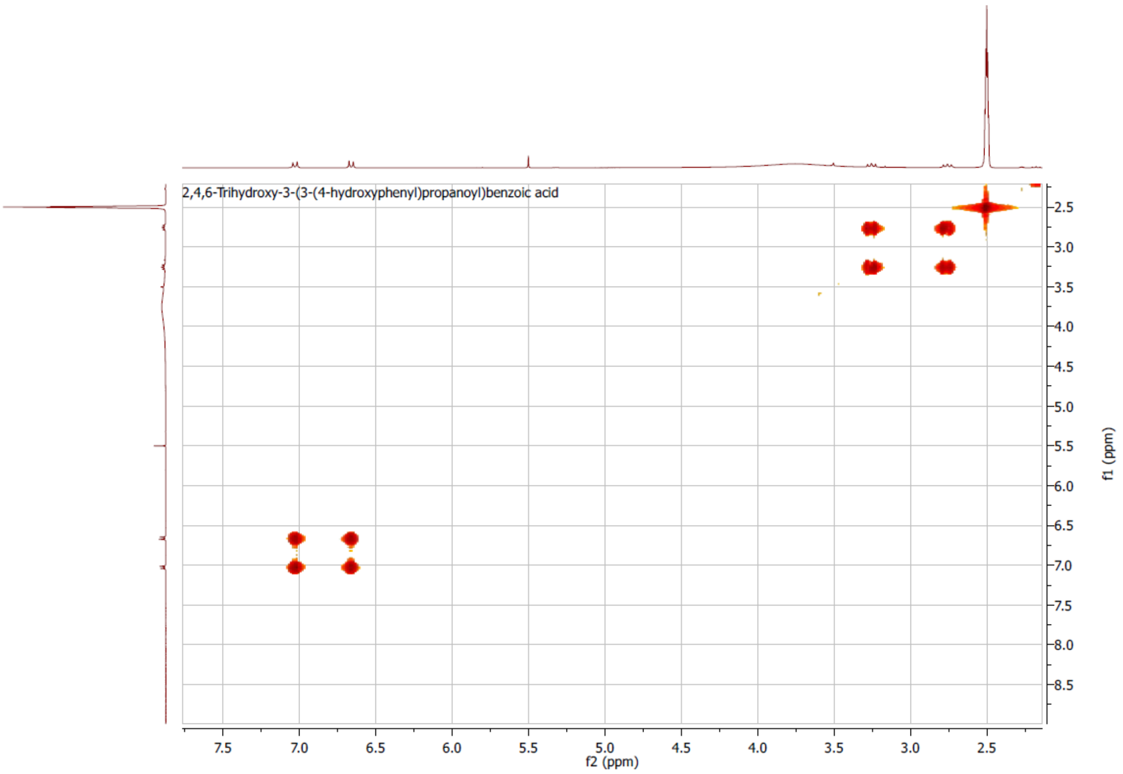
2,4,6-Trihydroxy-3-(3-(4-hydroxyphenyl)propanoyl)benzoic acid
f2 (ppm)
f1 (ppm)

HMBC:

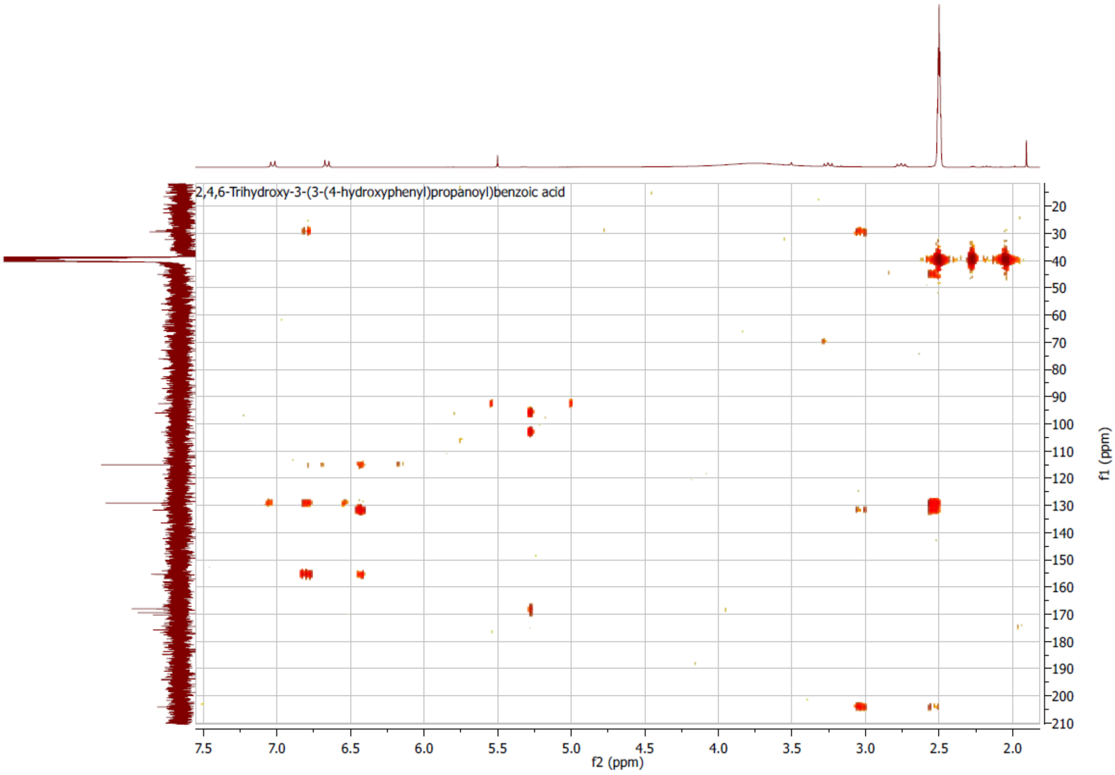

High resolution MS:

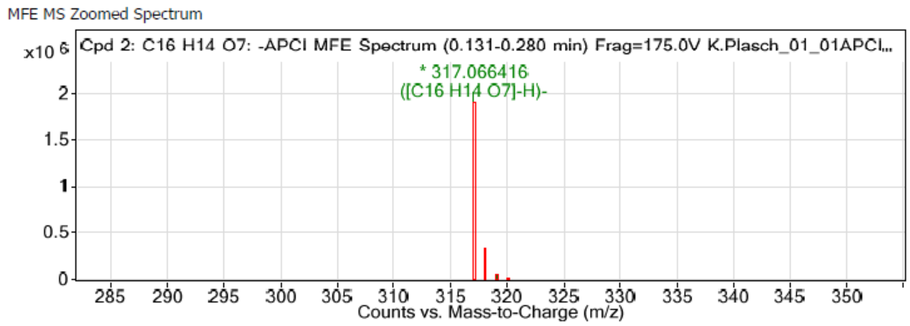

Calculated:	317.066676
Found:	317.066416
Mass accuracity:	0.82 ppm

(*E*)-2,6-Dihydroxy-4-(4-hydroxystyryl)benzoic acid **7b**

^{1}H NMR:

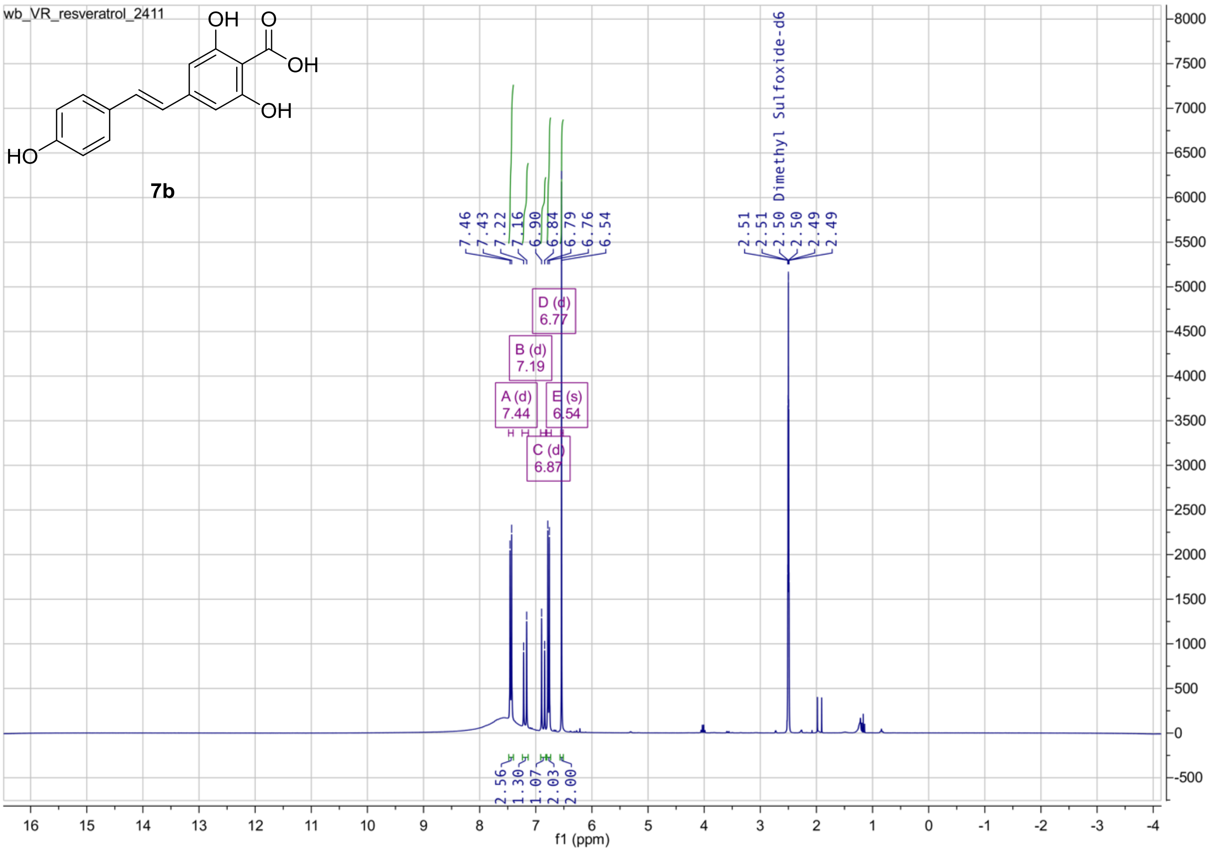

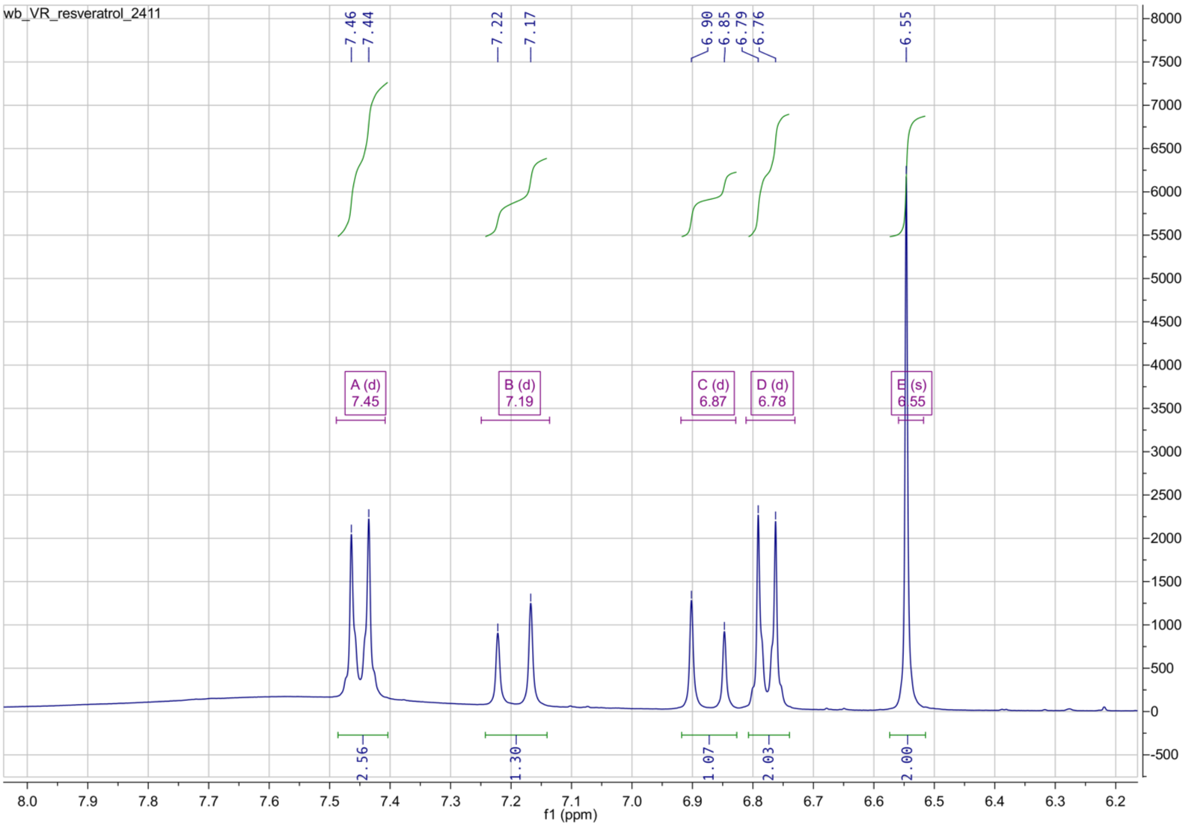

^{13}C NMR:

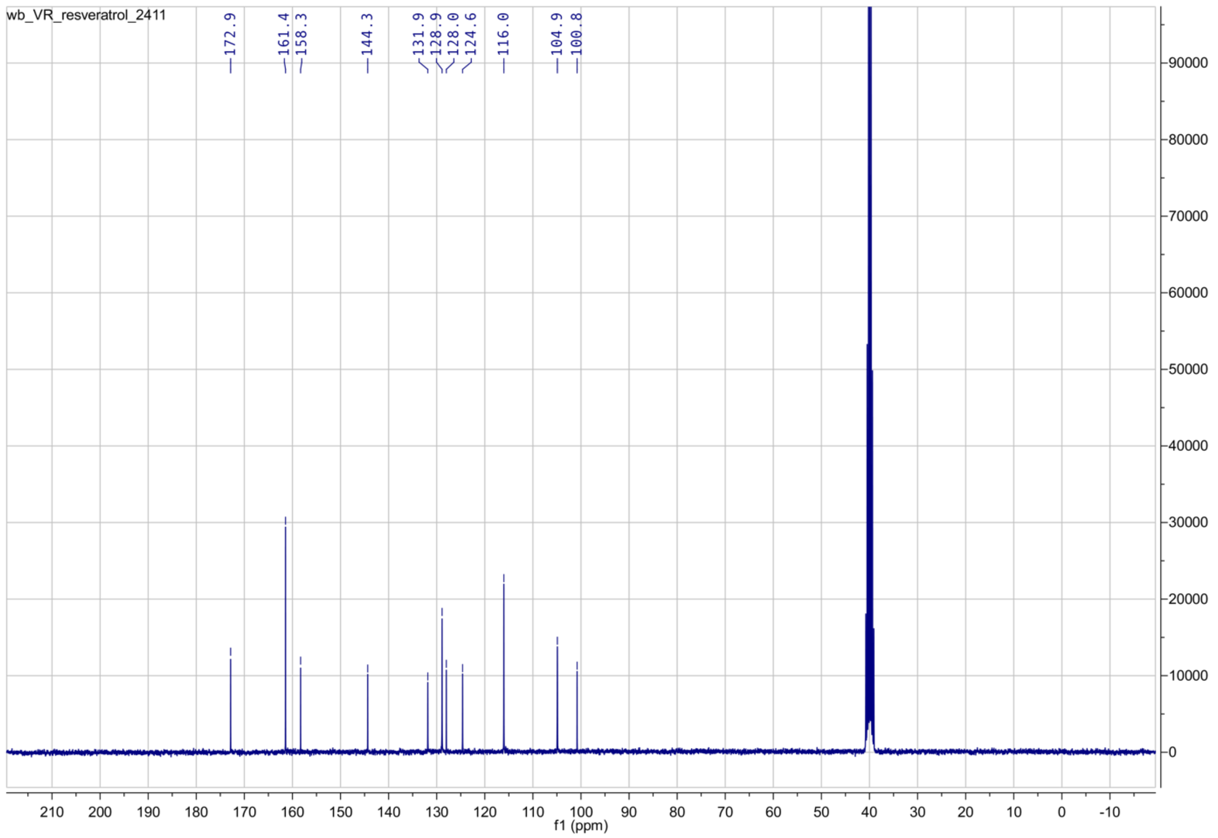

wb_VR_resveratrol_2411
172.9
161.4
158.3
144.3
131.9
128.9
128.0
124.6
116.0
104.9
100.8
f1 (ppm)

COSY:

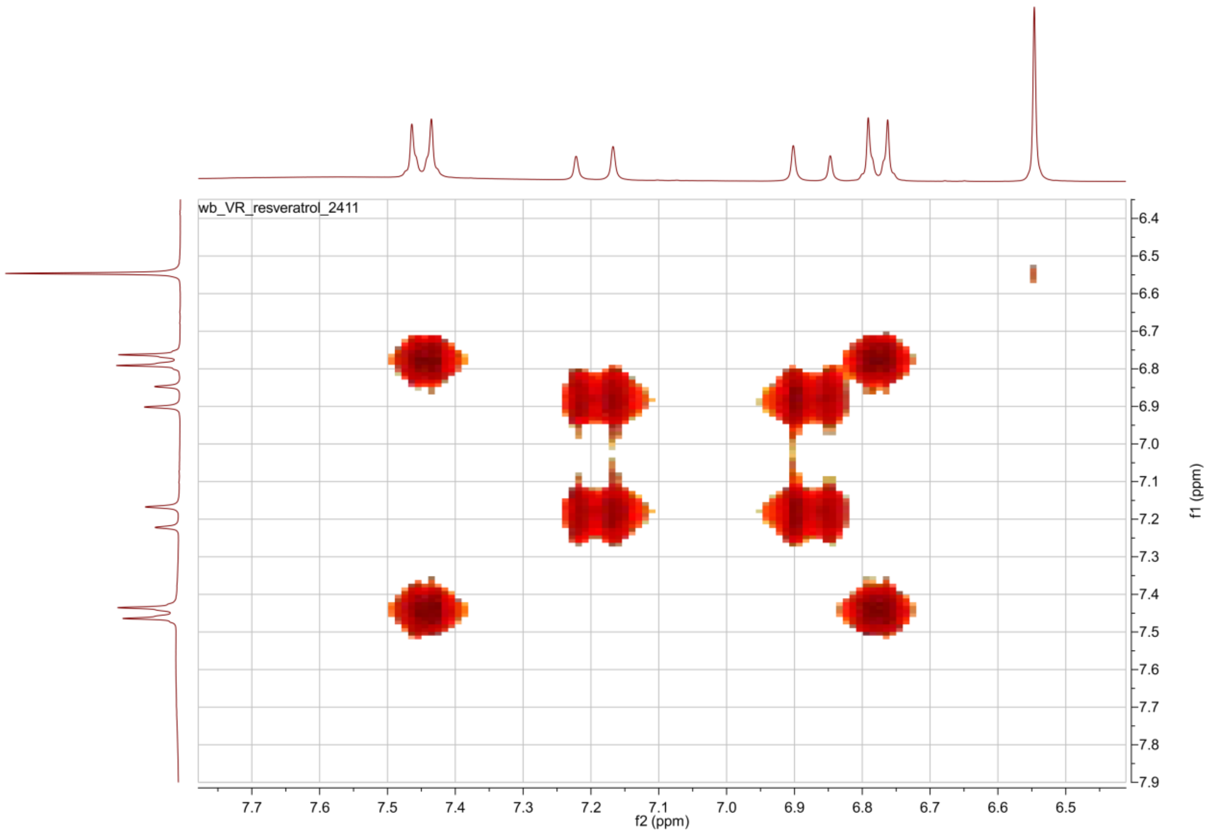

wb_VR_resveratrol_2411
f2 (ppm)
f1 (ppm)

HSQC:

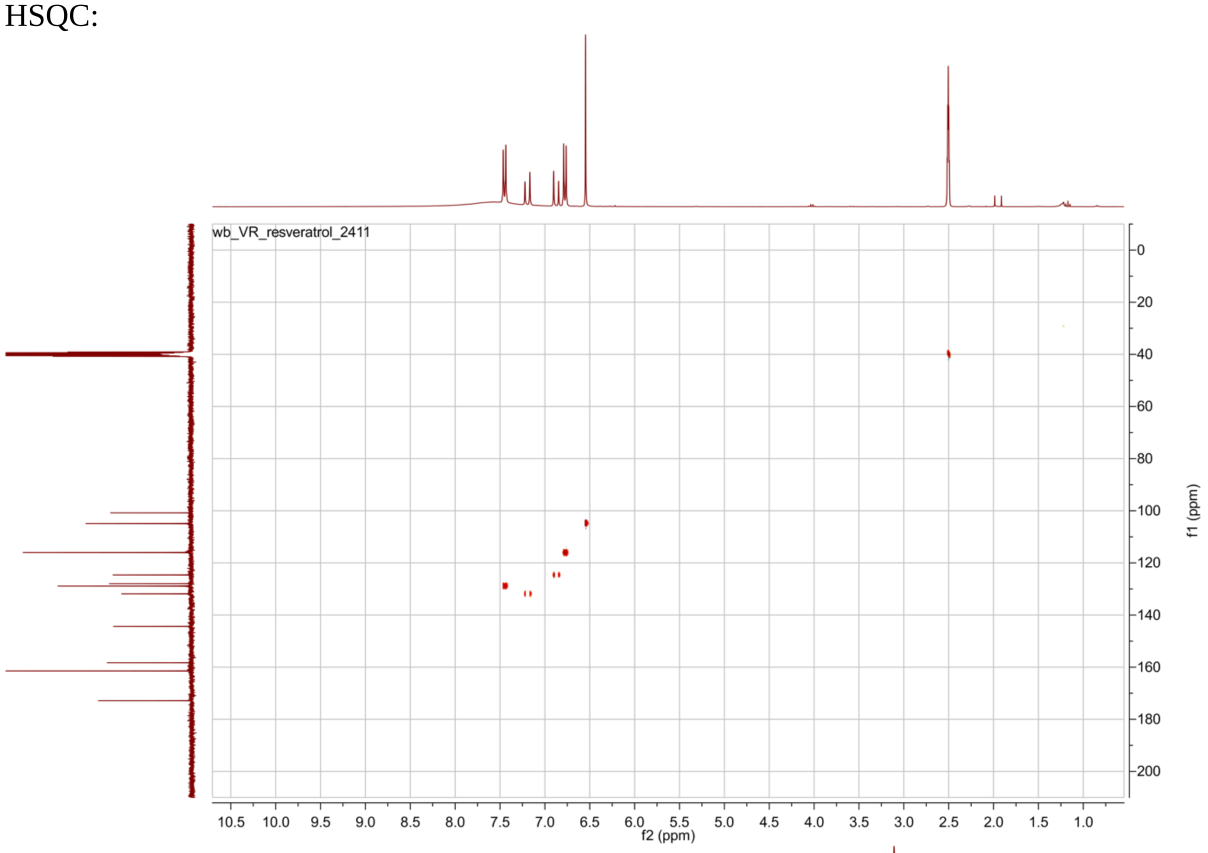

wb_VR_resveratrol_2411
f2 (ppm)
f1 (ppm)

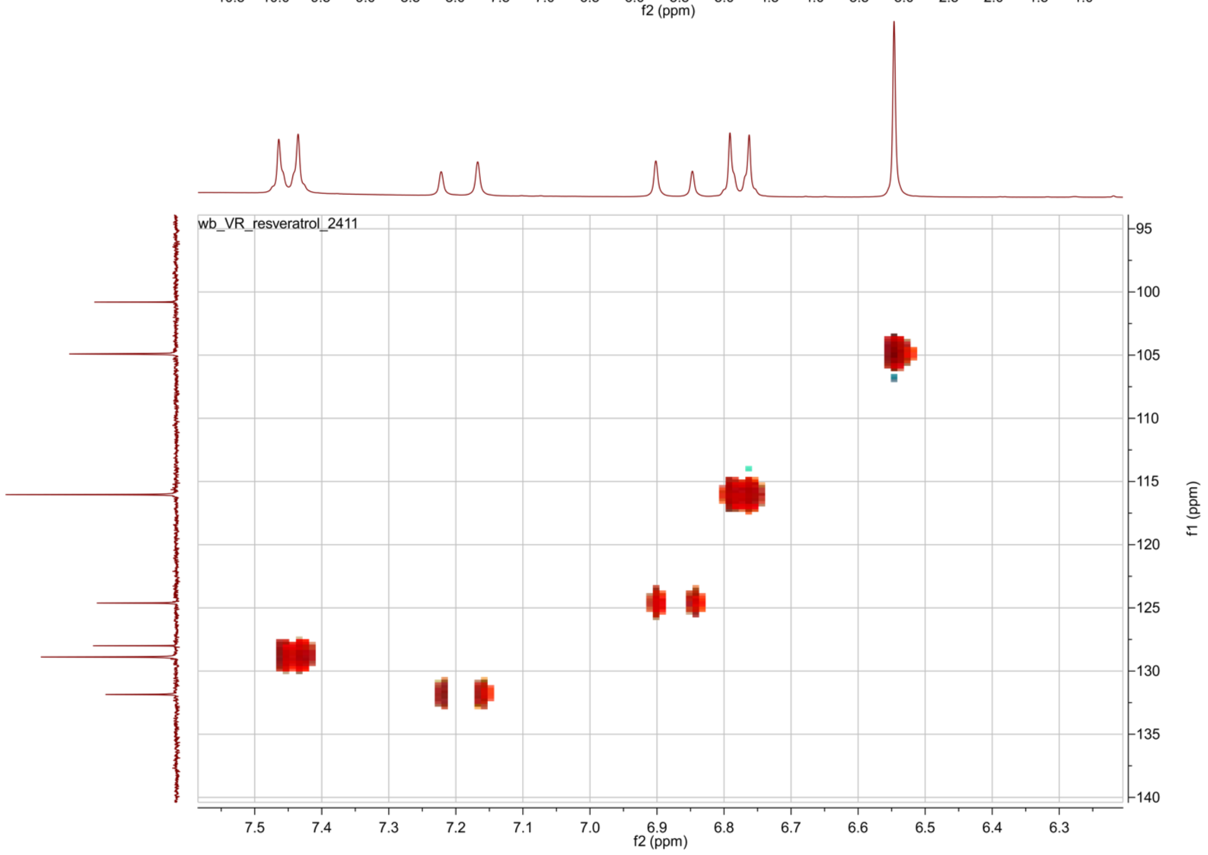

wb_VR_resveratrol_2411
f2 (ppm)
f1 (ppm)

HMBC:

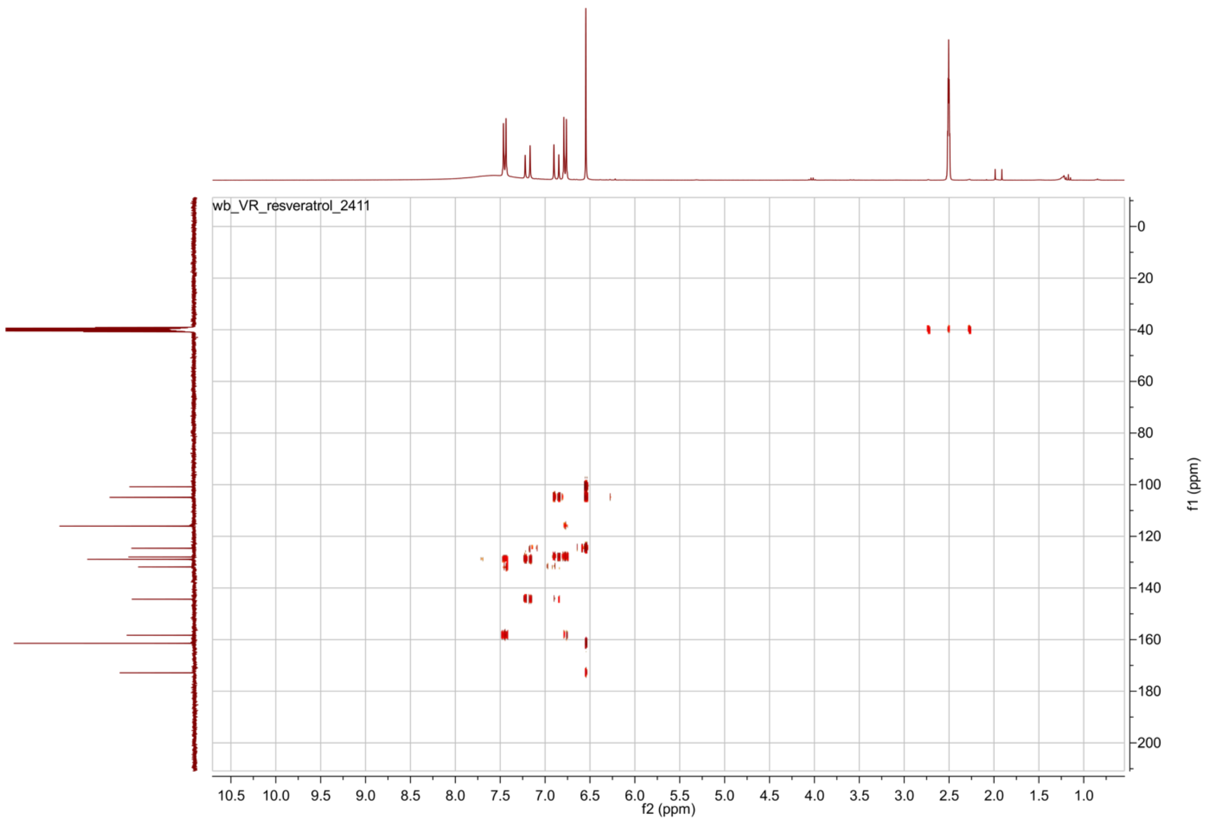

wb_VR_resveratrol_2411
f2 (ppm)
f1 (ppm)

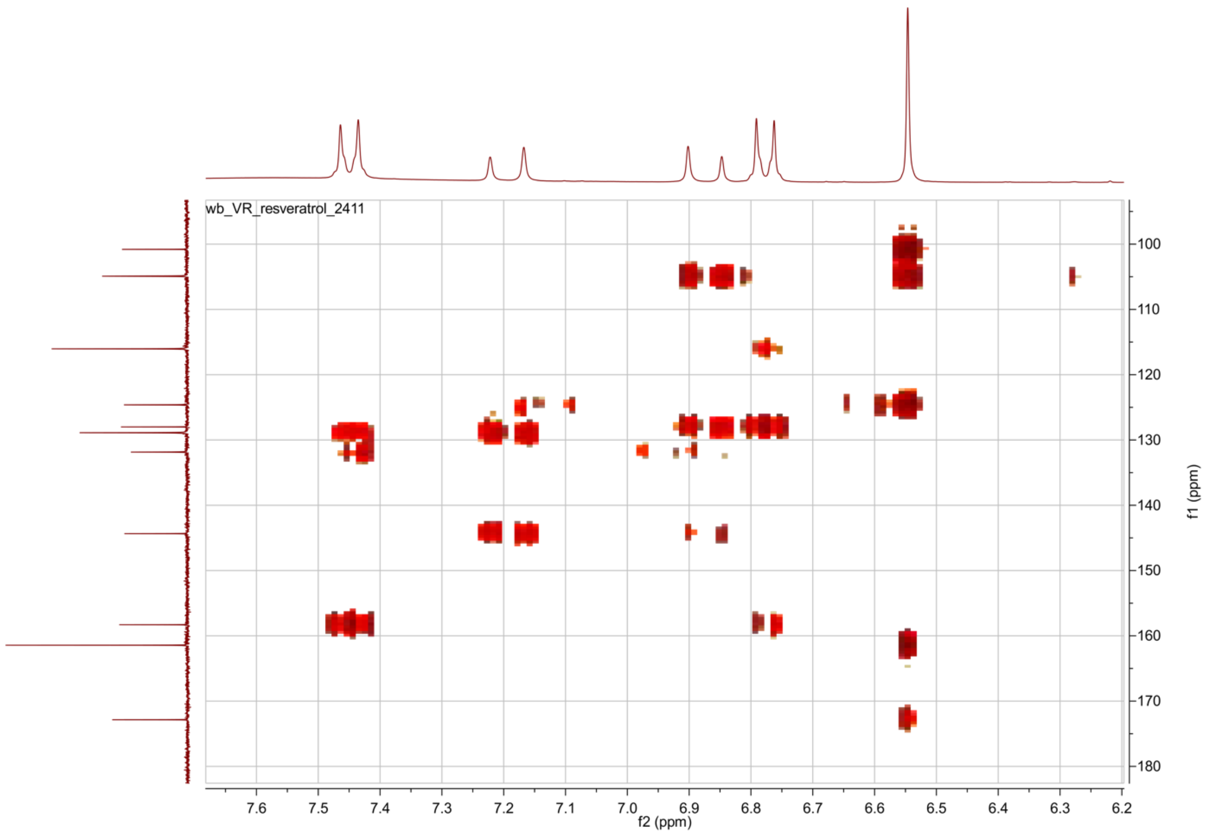

wb_VR_resveratrol_2411
f2 (ppm)
f1 (ppm)

High resolution MS:

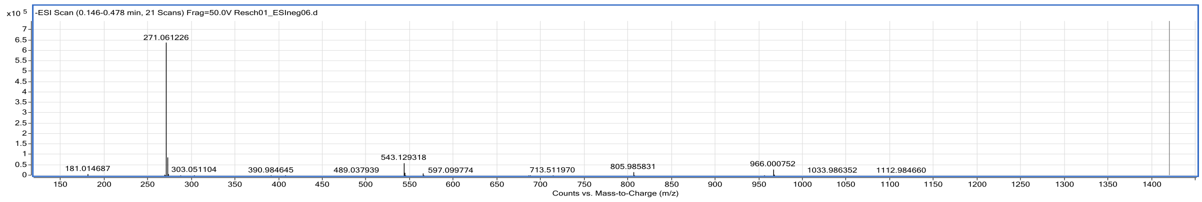

Calculated: 271.061197

Found: 271.061226

Mass accuracity -0,11 ppm

2,6-Dihydroxy-4-(4-hydroxyphenethyl)benzoic acid **8b**

^{1}H NMR:

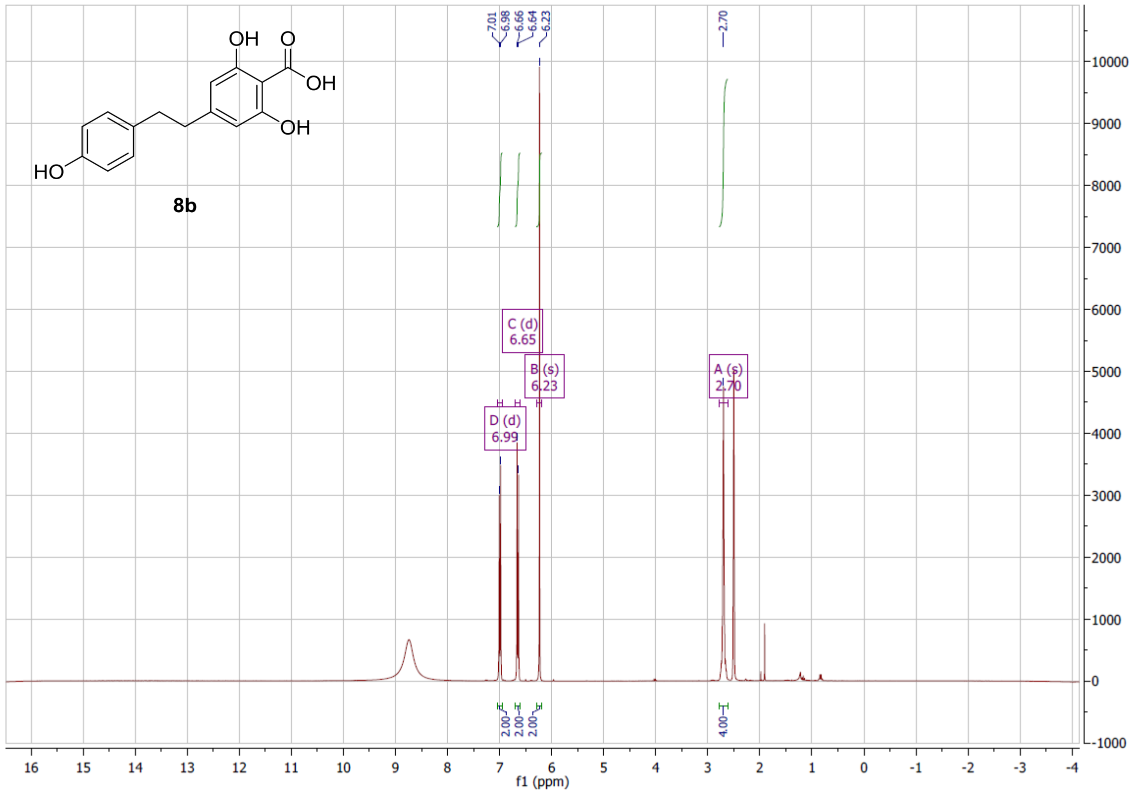

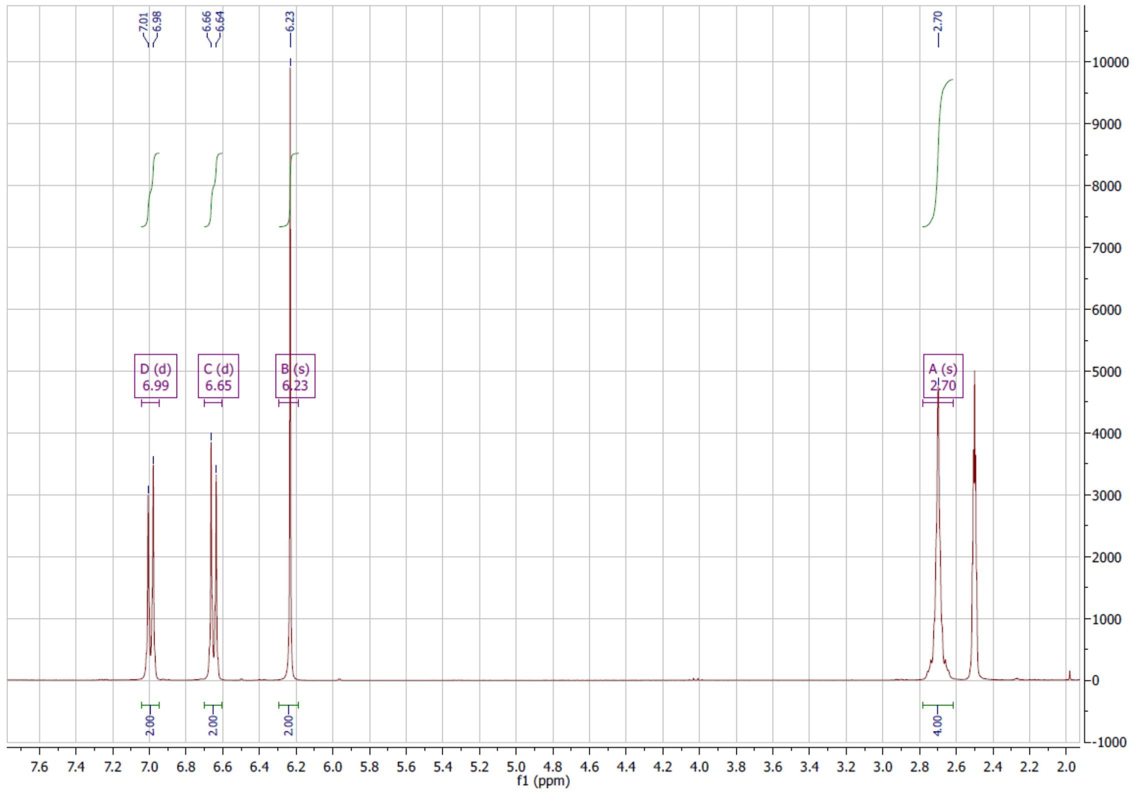

^{13}C NMR:

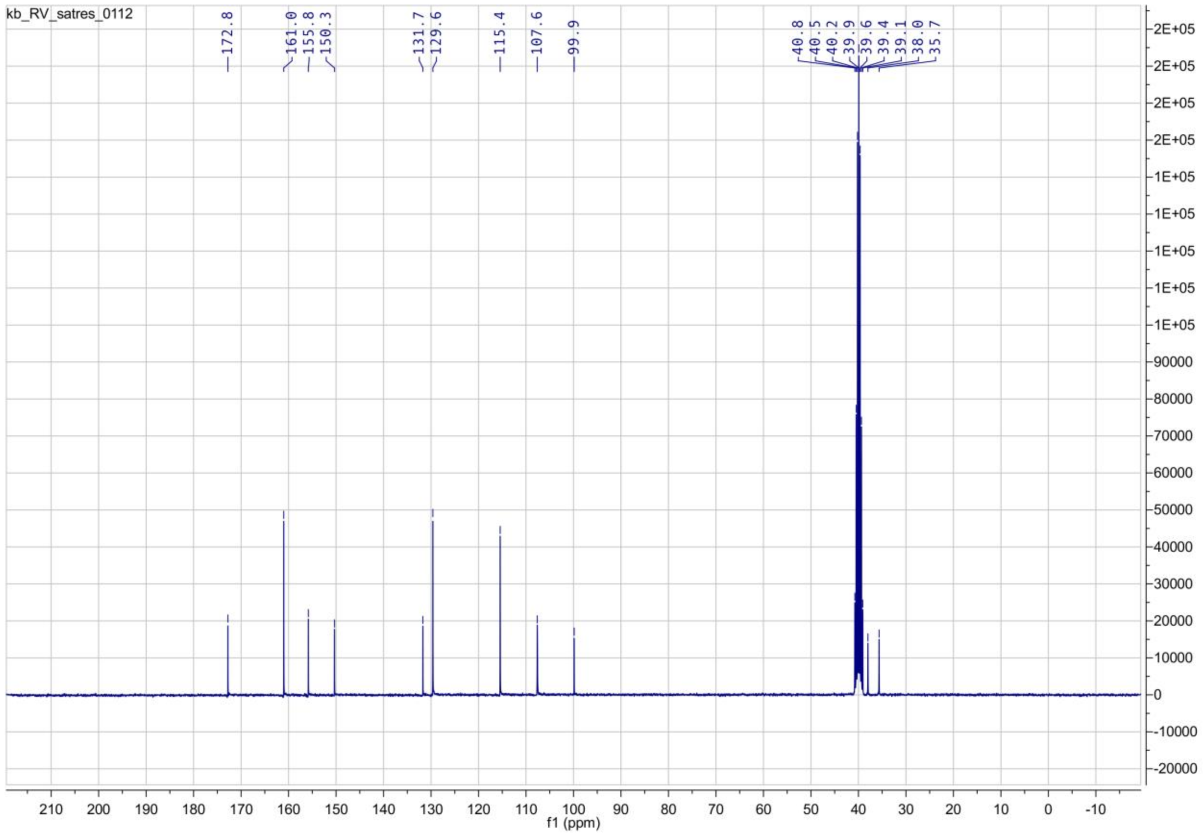
kb_RV_satres_0112
172.8
161.0
155.8
150.3
131.7
129.6
115.4
107.6
99.9
40.8
40.5
40.2
39.9
39.6
39.4
39.1
38.0
35.7
f1 (ppm)

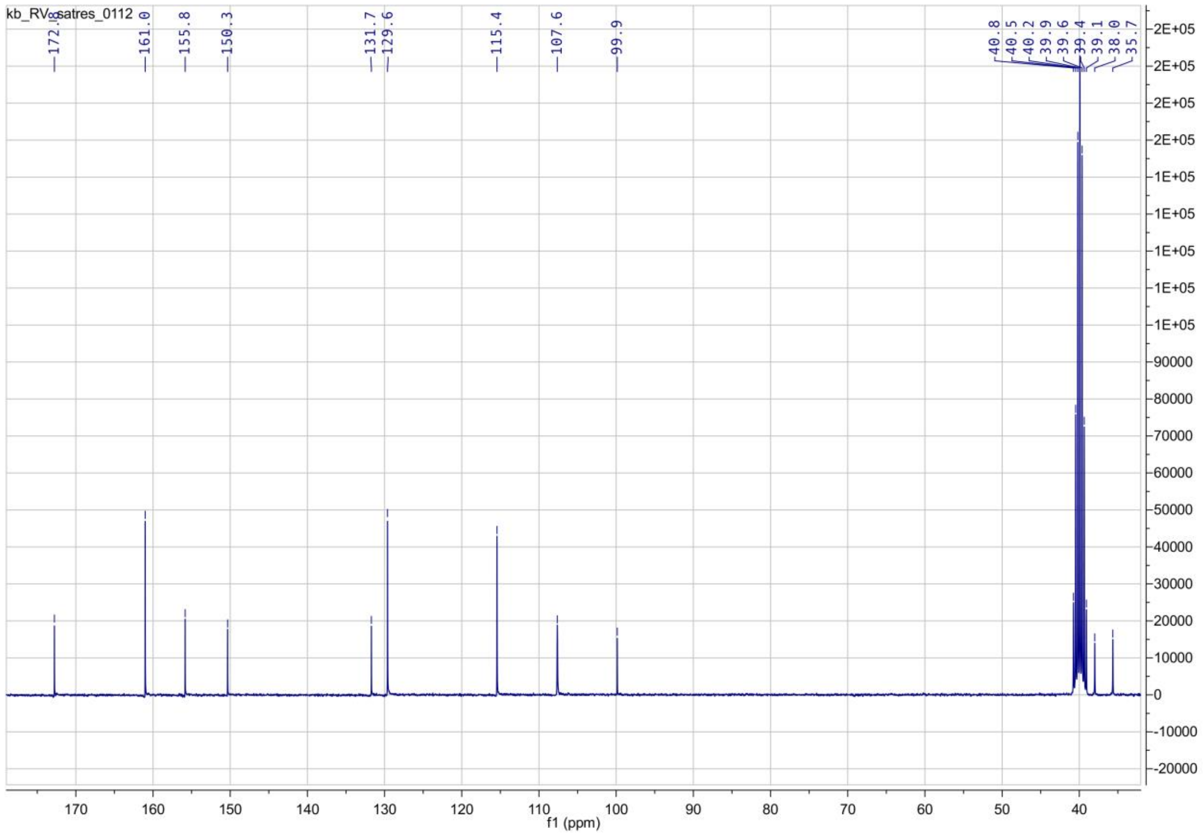
kb_RV_satres_0112
172.8
161.0
155.8
150.3
131.7
129.6
115.4
107.6
99.9
40.8
40.5
40.2
39.9
39.6
39.4
39.1
38.0
35.7
f1 (ppm)

HSQC:

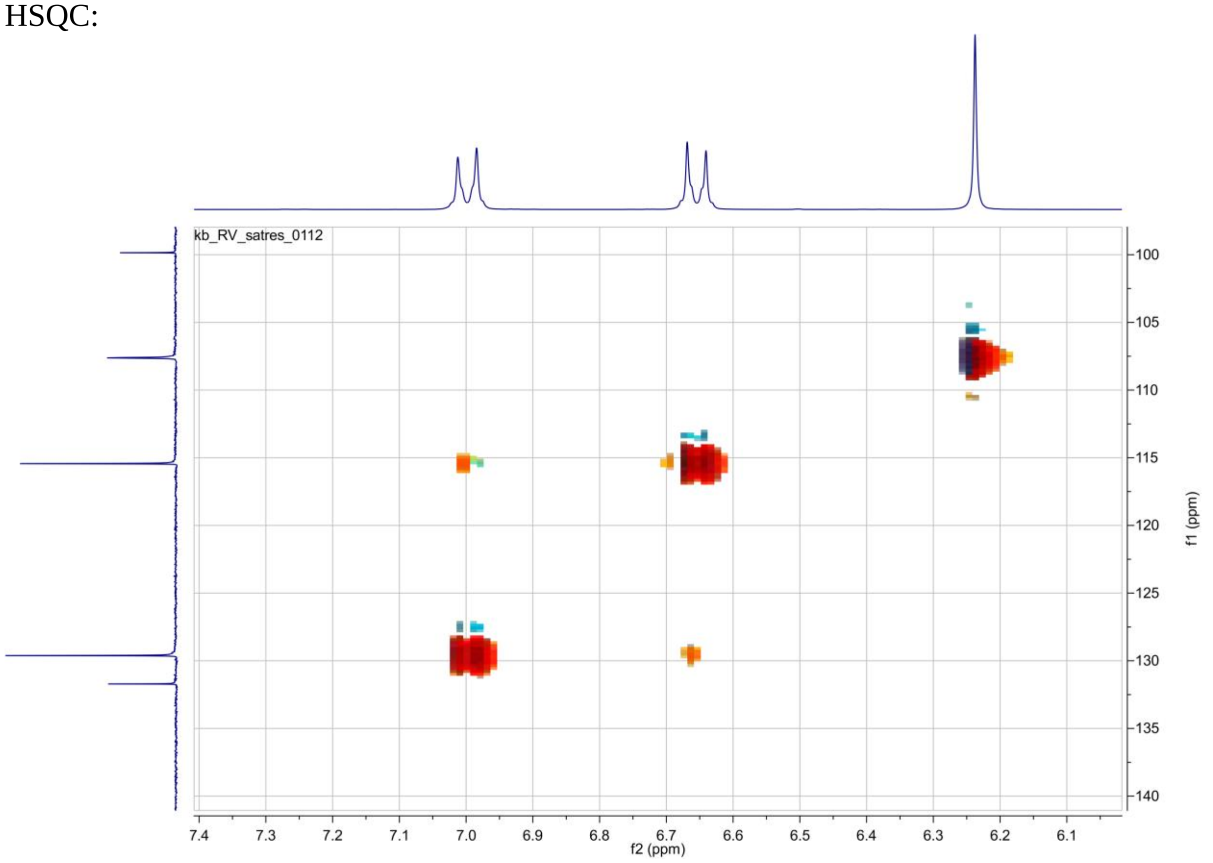

COSY:

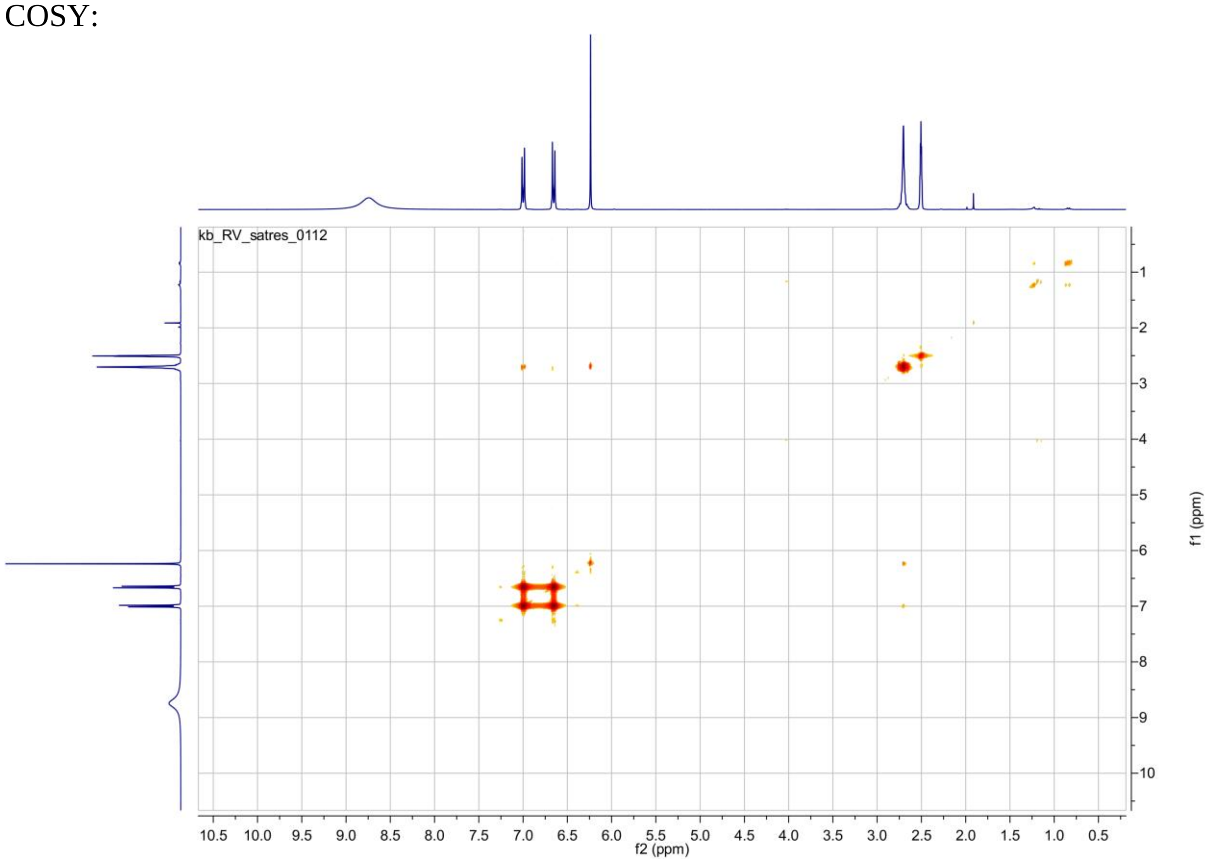

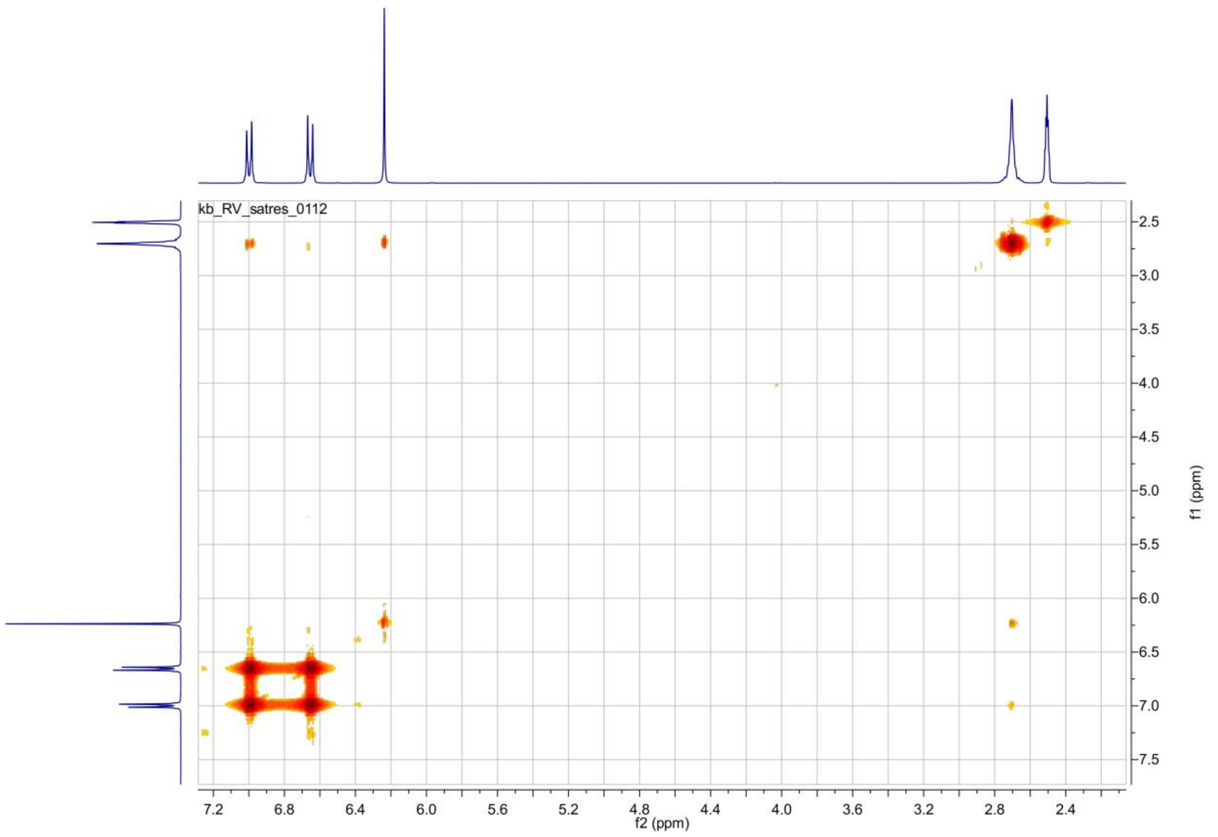

HMBC:

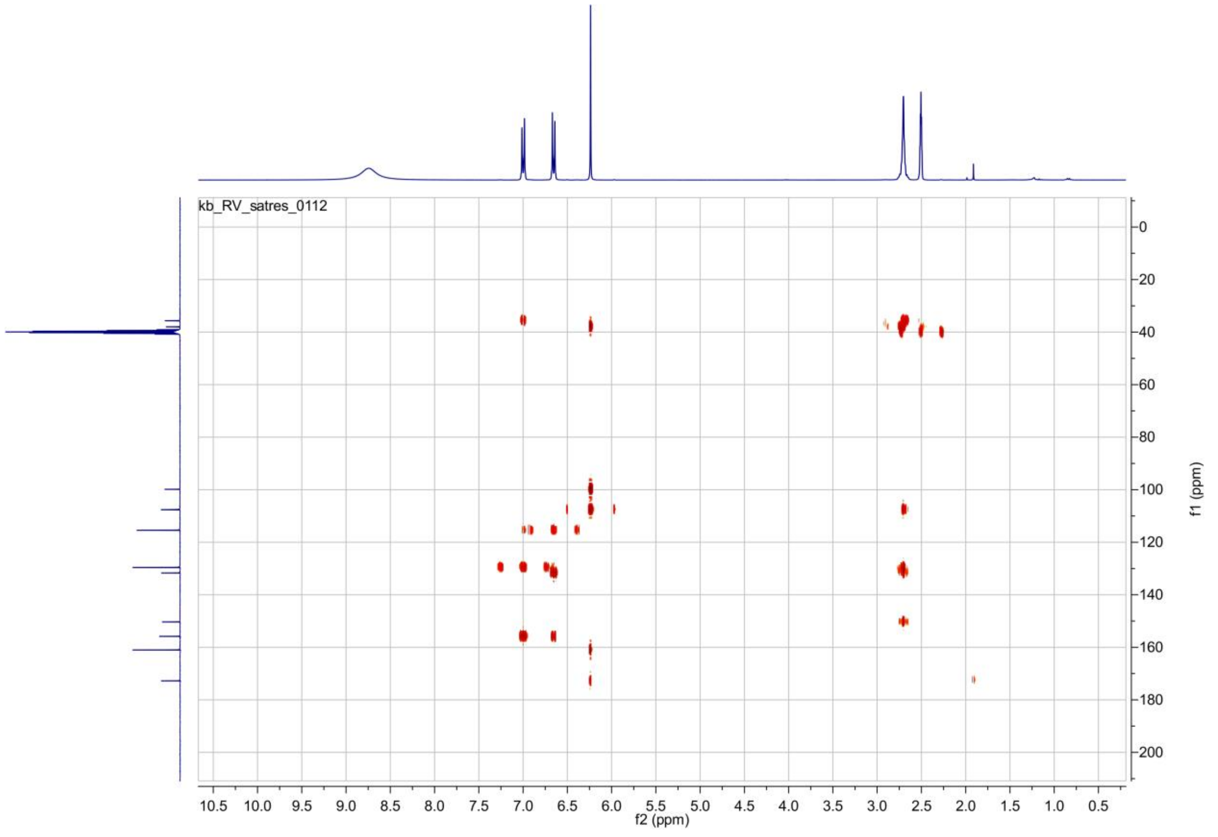

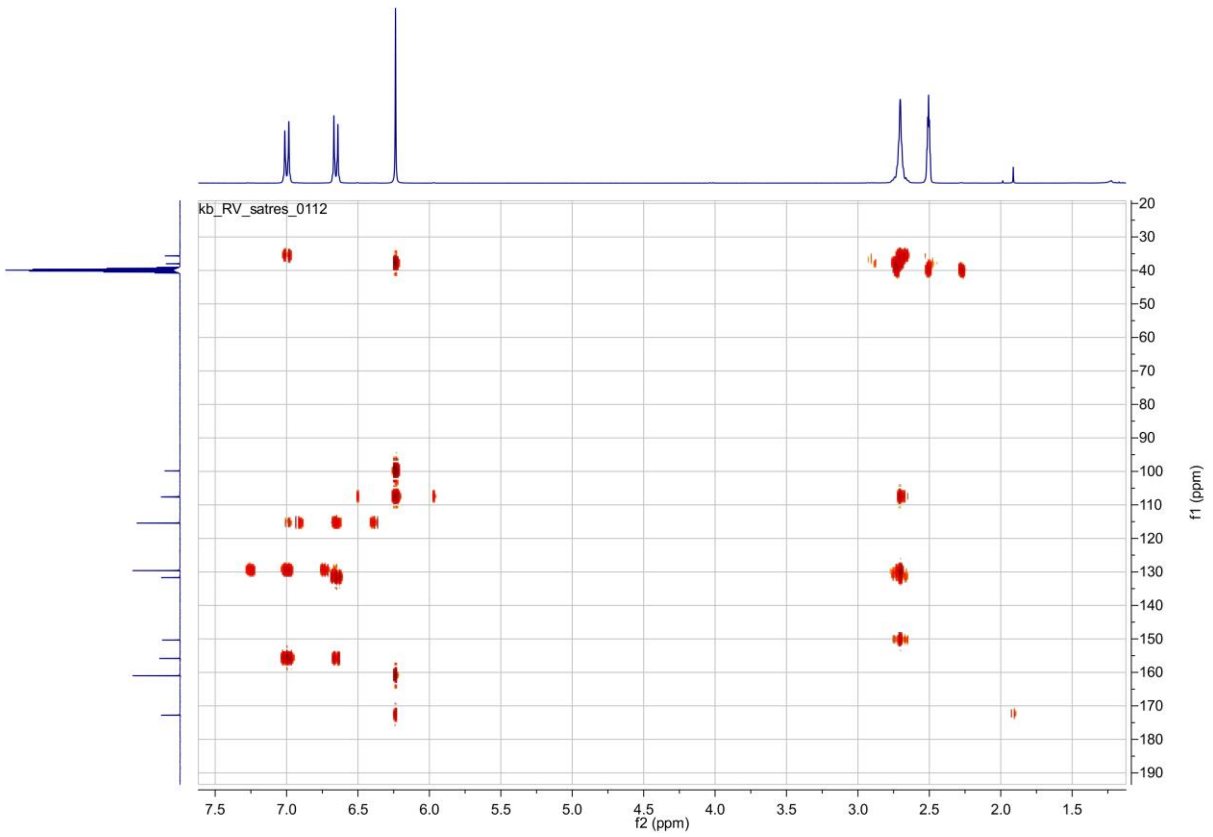

High resolution MS:

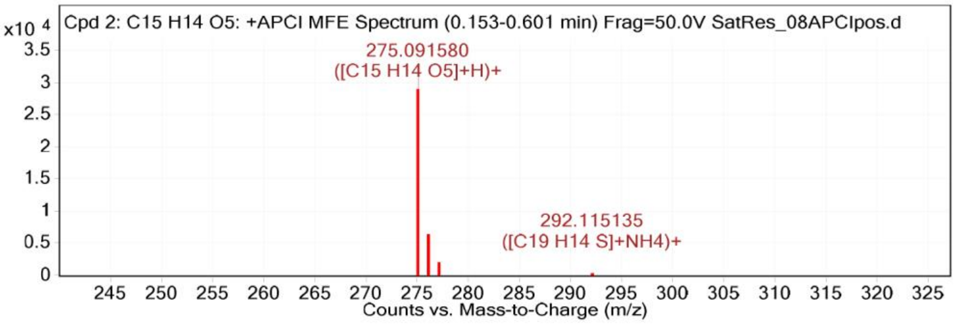

Calculated: 275.09140
Found: 275.09158
Mass accuracity -0,65ppm

(*E*)-4-(2,4-Dihydroxystyryl)-2,6-dihydroxybenzoic acid 9**b**

^{1}H NMR:

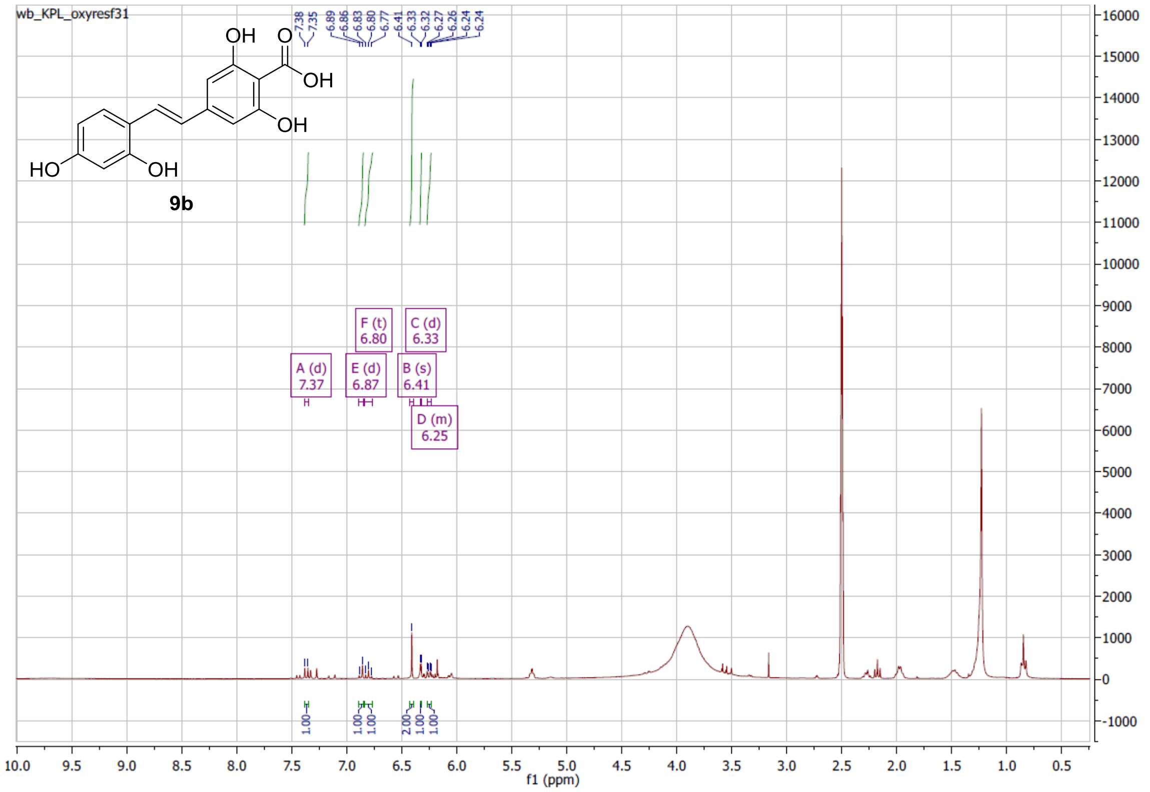

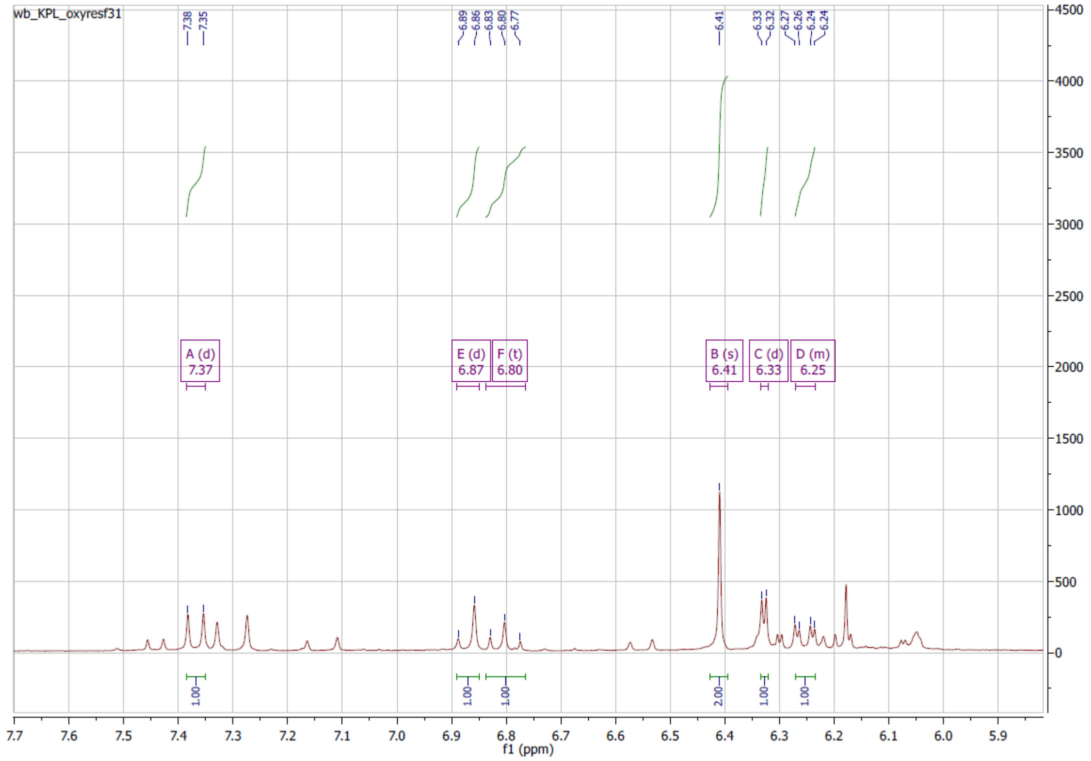

^{13}C NMR:

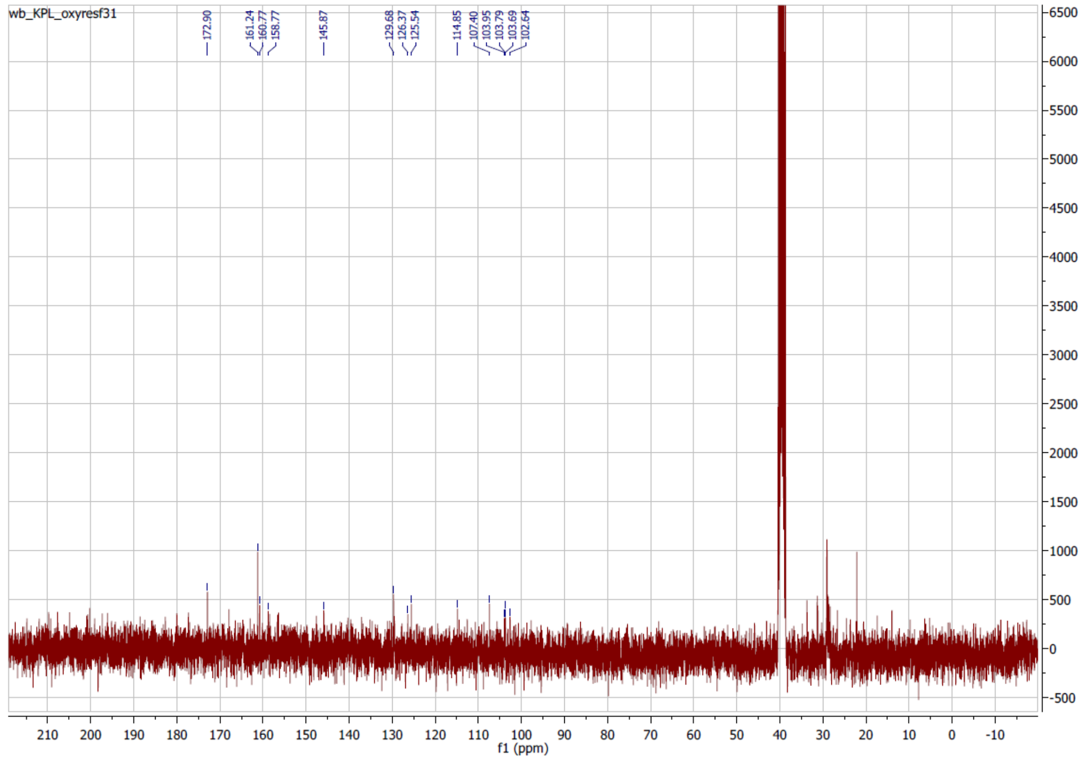
wb_KPL_oxyresf31
172.90
161.24
160.77
158.77
145.87
129.68
126.37
125.54
114.85
107.40
103.95
103.79
103.69
102.64
f1 (ppm)

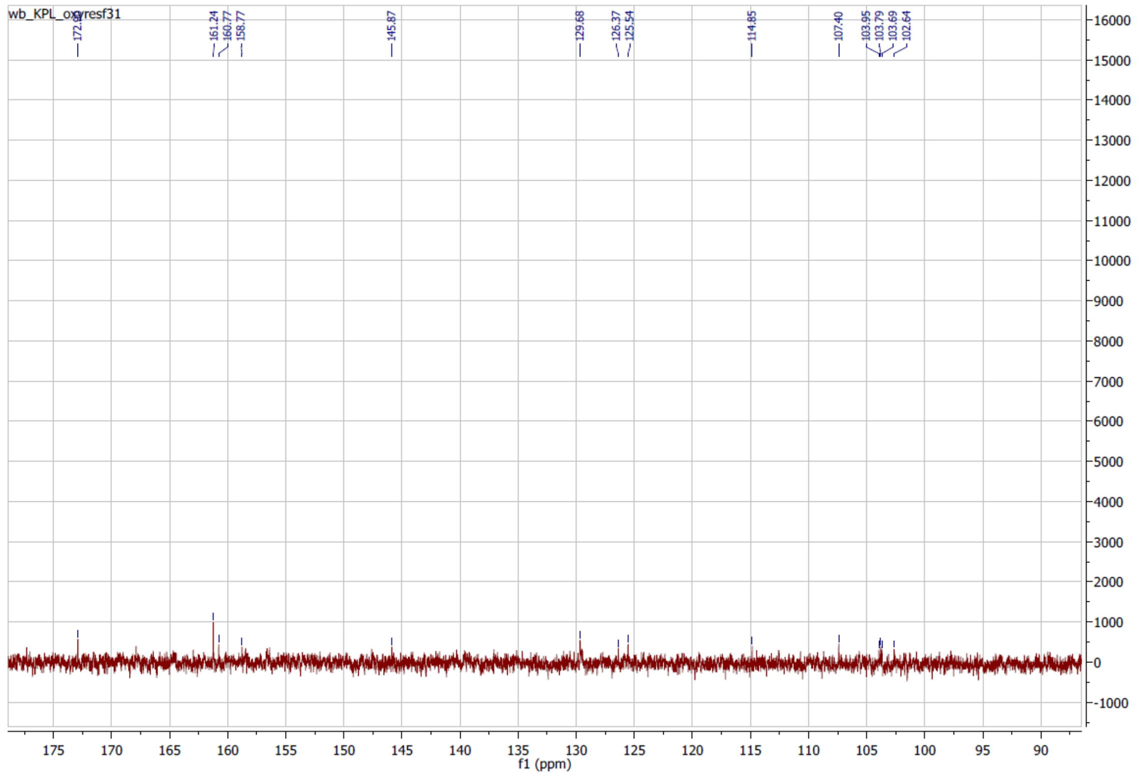
wb_KPL_oxyresf31
161.24
160.77
158.77
145.87
129.68
126.37
125.54
114.85
107.40
103.95
103.79
103.69
102.64
f1 (ppm)

(*E*)-3-(4-Carboxy-3,5-dihydroxystyryl)-2,6-dihydroxybenzoic acid **9c**

^{1}H NMR:

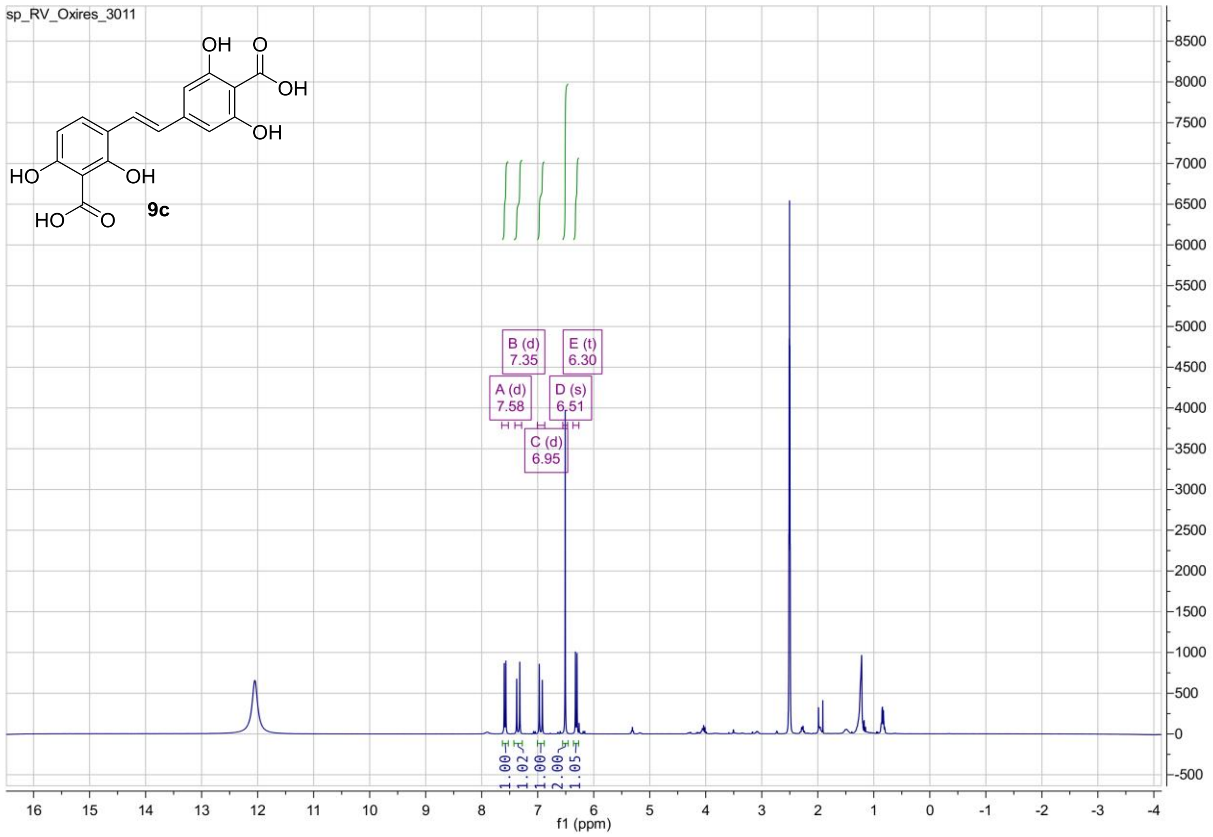

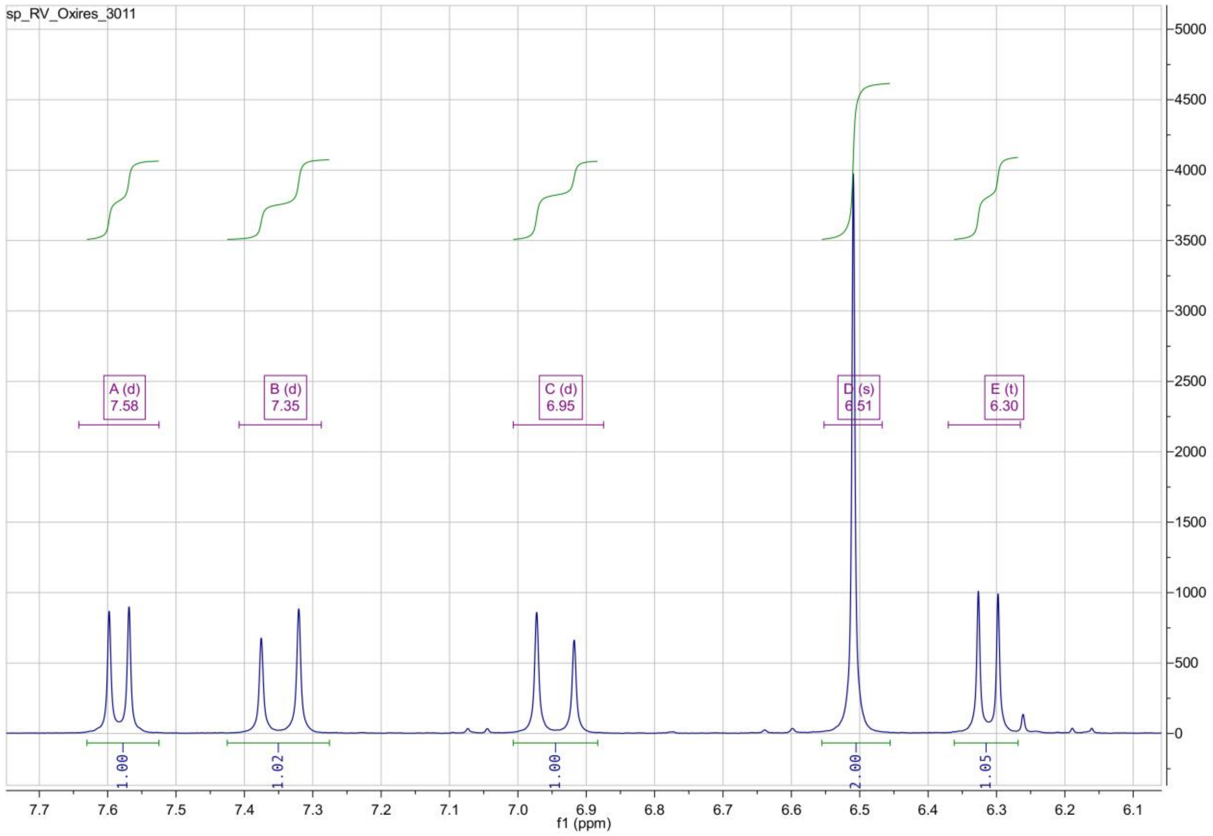

HSQC:

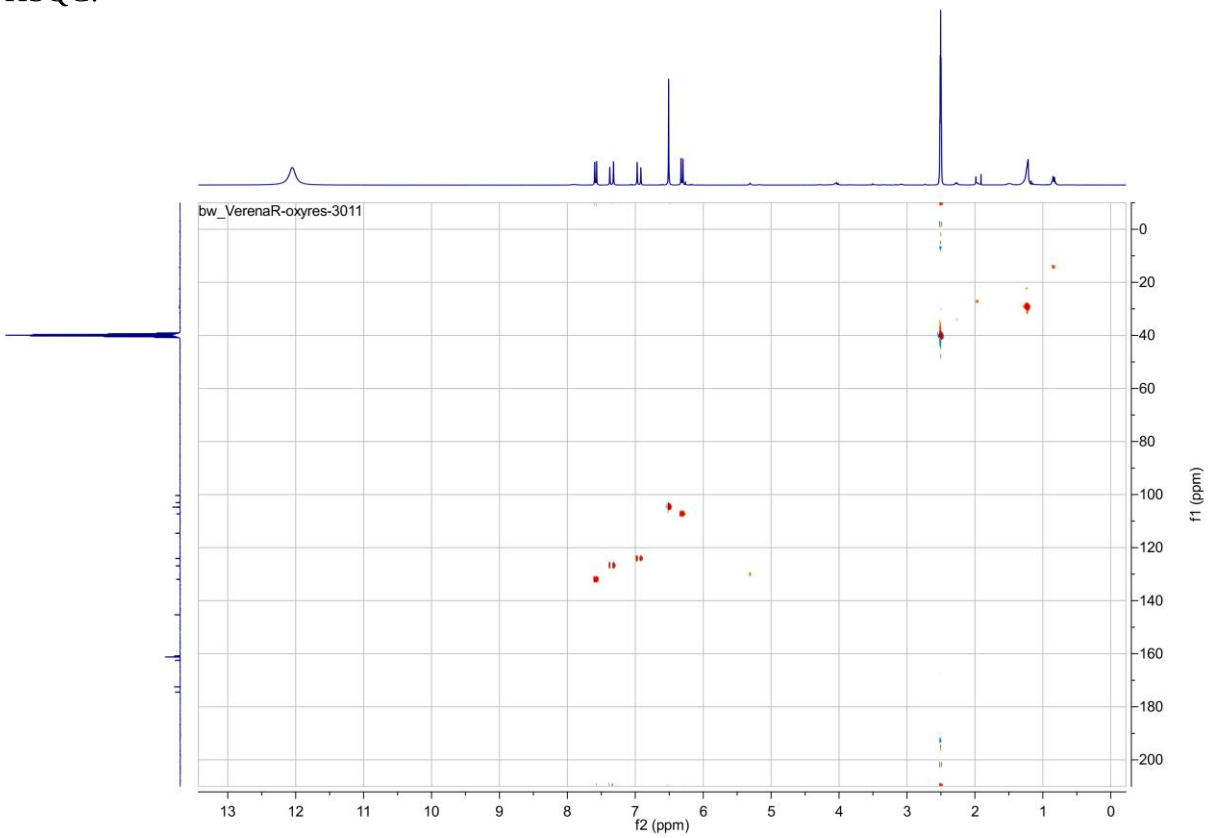
bw_VerenaR-oxyres-3011
f2 (ppm)
f1 (ppm)

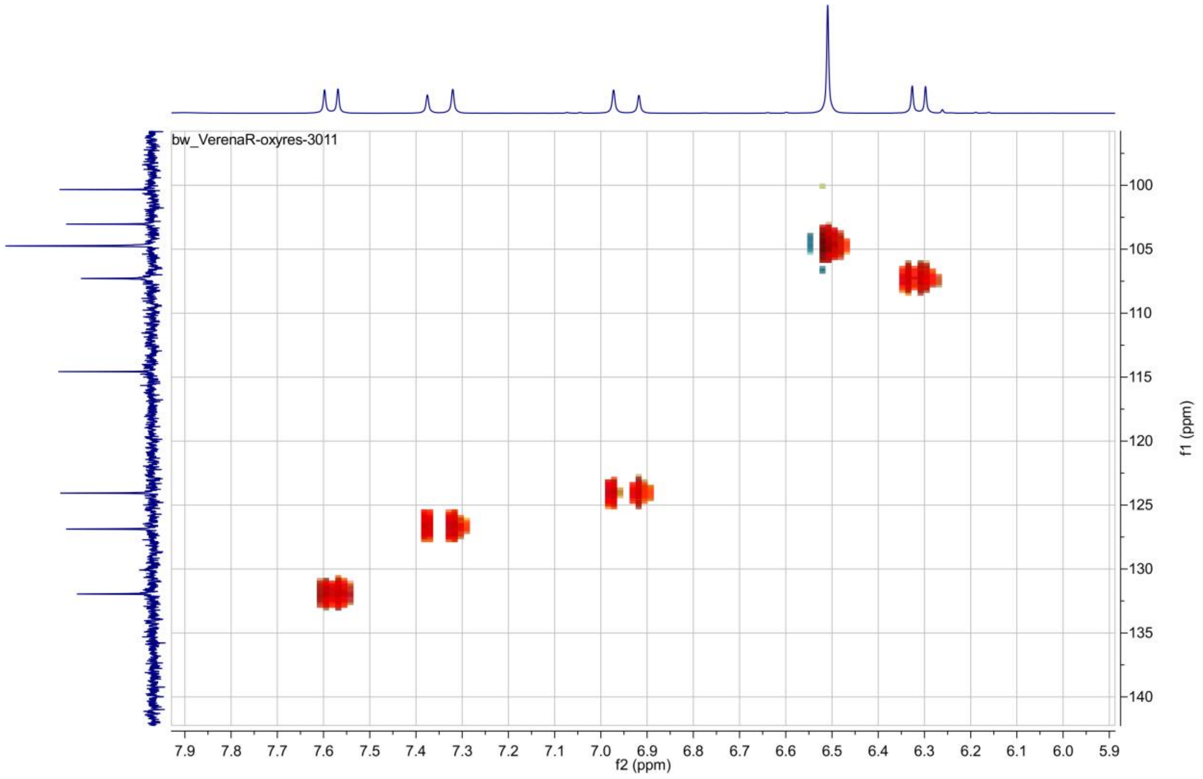
bw_VerenaR-oxyres-3011
f2 (ppm)
f1 (ppm)

COSY:

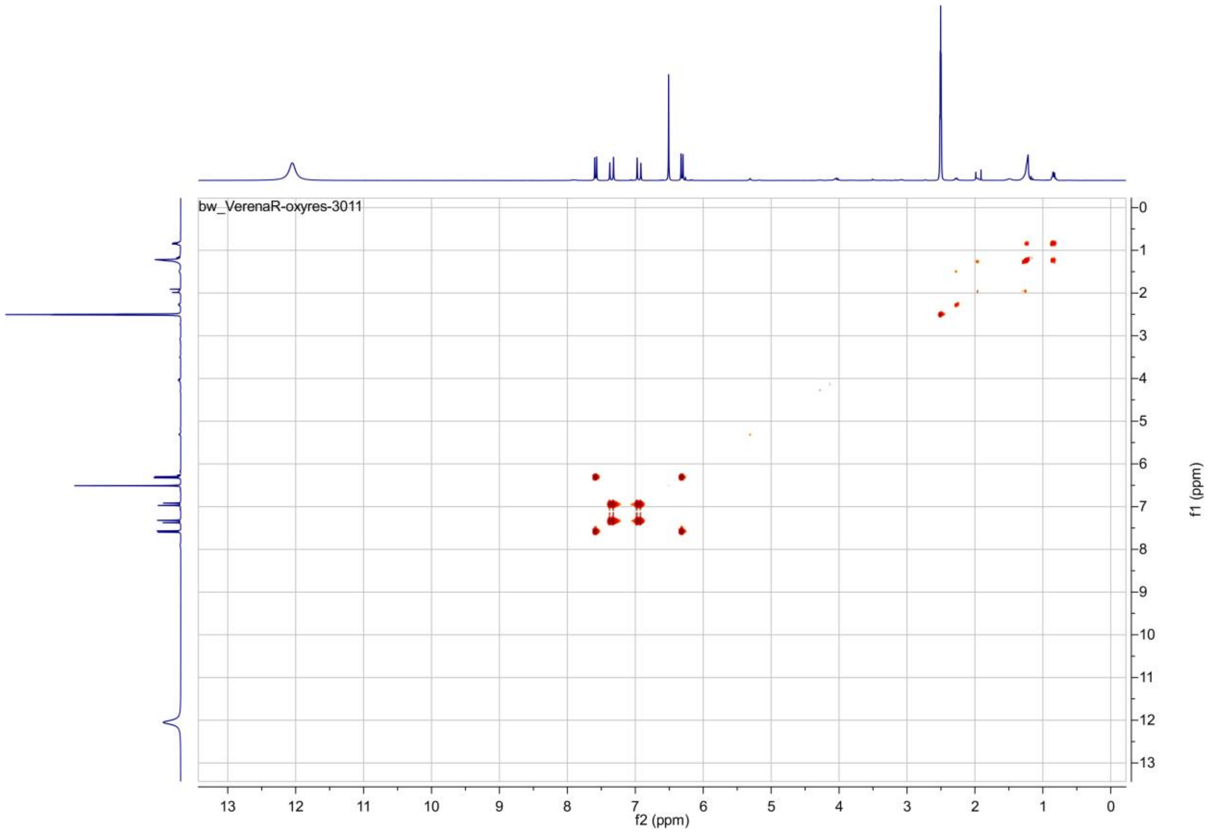
bw_VerenaR-oxyres-3011
f2 (ppm)
f1 (ppm)
13
12
11
10
9
8
7
6
5
4
3
2
1
0

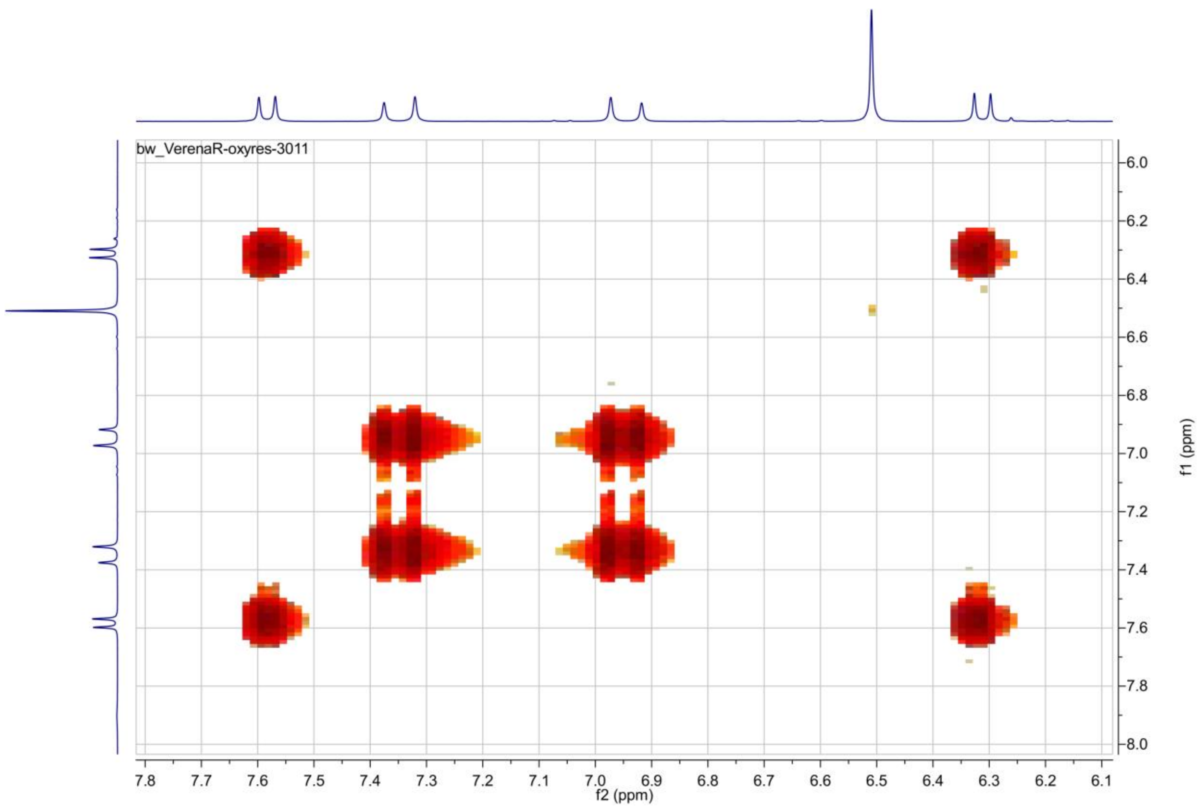
bw_VerenaR-oxyres-3011
f2 (ppm)
f1 (ppm)
7.8
7.7
7.6
7.5
7.4
7.3
7.2
7.1
7.0
6.9
6.8
6.7
6.6
6.5
6.4
6.3
6.2
6.1
6.0
8.0

HMBC:

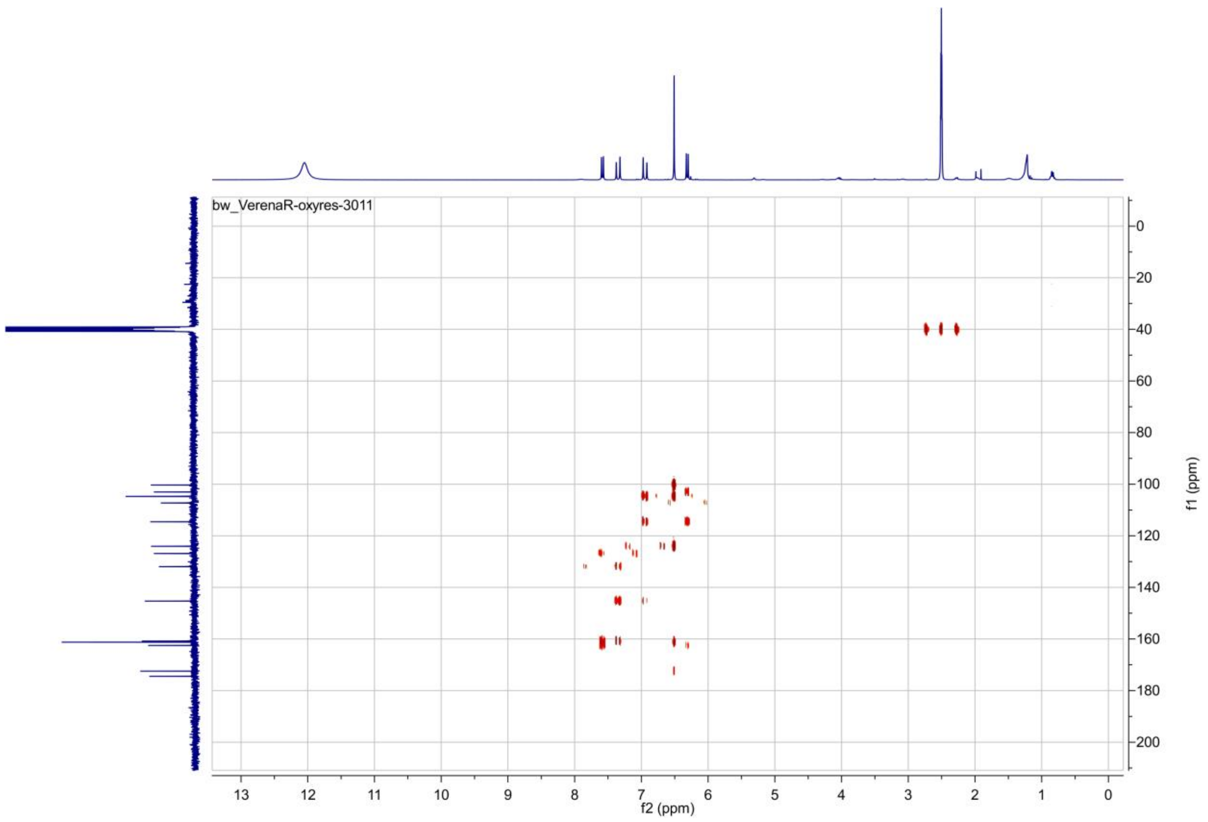
bw_VerenaR-oxyres-3011
f2 (ppm)
f1 (ppm)

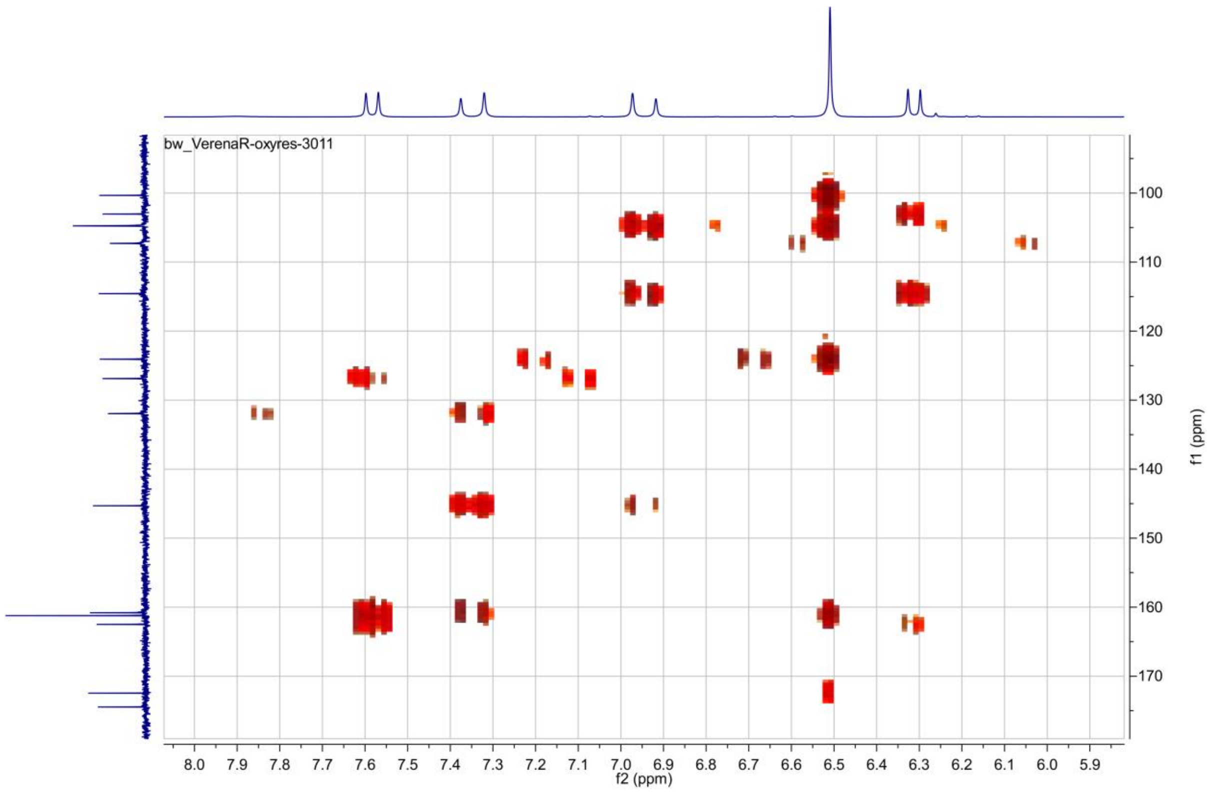
bw_VerenaR-oxyres-3011
f2 (ppm)
f1 (ppm)

High resolution MS:

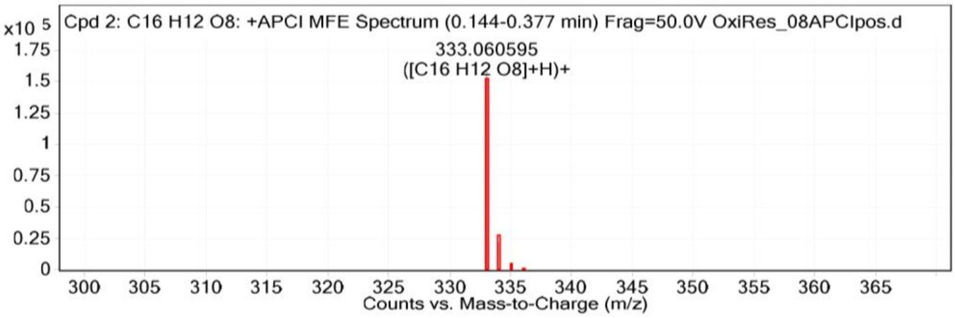

Calculated:	333.060494
Found:	333.060595
Mass accuracity	-0,30 ppm

8. References

[1] C. Wuensch, S. M. Glueck, J. Gross, D. Koszelewski, M. Schober, K. Faber, *Org. Lett.* **2012**, *14*, 1974–1977.

[2] G. Allegretta, E. Weidel, M. Empting, R. W. Hartmann, *Eur. J. Med. Chem.* **2015**, *90*, 351-359.

[3] B. Forman, D. Yu, Synthetic ligands selective for LXRbeta over LXRalpha, identification and methods of use thereof, US2009/30082 A1, 2009-01-29; CAN: *150*:167983.

[4] S. Iwata, T. Nishino, N. Nagata, Y. Satomi, H. Nishino, S. Shibata, *Biol. Pharm. Bull.* **1995**, *18*, 1710 – 1713.

[5] W. S. Murphy, S. Wattanasin, *J. Chem. Soc., Perkin Trans. 1*, **1980**, 1567-1577.

[6] Y. Ishii, Y. Narimatsu, Y. Iwasaki, N. Arai, K. Kino, K. Kirimura, *Biochem. Biophys. Res. Commun.* **2004**, *324*, 611-620.

[7] M. Yoshida, N. Fukuhara, T. Oikawa, *J. Bacteriol.* **2004**, *186*, 6855-6863

[8] T. Yoshida, Y. Hayakawa, T. Matsui, T. Nagasawa, *Arch. Microbiol.* **2004**, *181*, 391-397

[9] M. Sato, N. Sakurai, H. Suzuki, D. Shibata, K. Kino, *J. Mol. Catal. B: Enzym.* **2015**, *122*, 348-352.

[10] B. Schmidt, F. Hölter, R. Berger, S. Jessel, *Adv. Synth. Catal.* **2010**, *352*, 2463-2473.

[11] H.-L. Wang, C. Balan, E. M. Doherty, J. R. Falsey, V. K. Gore, J. Katon, M. H. Norman, Vanilloid receptor ligands and their use in treatments, US2005/176726 A1, 2005-08-11; CAN: *143*:211934.
